# Systematic Characterization of In Vitro and In Vivo Metabolic Pathways and Identification of Novel Biomarkers of 26 Synthetic Cannabinoids

**DOI:** 10.3390/molecules30132682

**Published:** 2025-06-21

**Authors:** Yudie Ning, Tao Wang, Xiao Yang, Fang Guo, Yingwen Xu, Yuan Zhang, Kaile Wang, Meng Hu, Zhe Chen, Zhiwen Wei, Keming Yun

**Affiliations:** 1 School of Forensic Medicine, Shanxi Medical University, Jinzhong 030600, China; ningyudie2022@163.com (Y.N.); wangtao@sxmu.edu.cn (T.W.); yangxiao982022@163.com (X.Y.); 15234874838@163.com (F.G.); xuyingwen2023@163.com (Y.X.); 15333455842@163.com (Y.Z.); 13694741139@163.com (K.W.); hu.meng@sxmu.edu.cn (M.H.); chenzhe0322@163.com (Z.C.); 2 Shanxi Key Laboratory of Forensic Medicine, Jinzhong 030600, China; 3 Key Laboratory of Forensic Toxicology of Ministry of Public Security, Jinzhong 030600, China; 4 Key Laboratory of Forensic Medicine in Shanxi Province, Jinzhong 030600, China

**Keywords:** drug metabolism, metabolites, synthetic cannabinoids (SCs), new psychoactive substances (NPSs), toxicological analysis

## Abstract

In recent years, the harms and abuse of synthetic cannabinoids (SCs) have attracted extensive attention in society. Their structures have been updated rapidly, which brings great challenges for continuous detection and drug identification. The aim of this study was to elucidate the metabolites of 26 kinds of abused SCs produced in human liver microsomes (HLMs) and rats and to explore the metabolism of indole amides, indazole amides, azaindoles, naphthyl indoles, cyclopropylindoles, naphthyl benzimidazole, and naphthyl pyrrole SCs in vivo and in vitro. Human liver microsomes were incubated with SCs to simulate human metabolic processes, and the in vitro metabolic model of liver microsomes was established. After the SD rats were randomized into groups, 26 kinds of SCs and normal saline were injected respectively to establish the rat model after exposure. The metabolites were identified one by one using a UHPLC-Q-Exactive Orbitrap MS method to explore the metabolic law. A total of 609 metabolites were identified, involving 30 metabolic pathways. The metabolism of SCs was summarized from the parent nuclear group, the head group, the linking group, and the tail side chain, and the mass spectral fragmentation pattern of the metabolites was analyzed in order to provide reference for the examination and identification of SCs-related cases.

## 1. Introduction

A new psychoactive substance (NPS) is a drug analog obtained by criminals who modify the chemical structure of listed drugs to avoid being attacked. They offer advantages such as simple synthesis, high concealment, and significant hazards [1]. Synthetic cannabinoids (SCs) are new psychoactive substances with many kinds of substances, serious abuse [2,3], and huge social harm, mainly in the form of e-cigarettes, herbal mixtures, and other hidden [4,5,6]. It escalates rapidly and has a diverse chemical structure to avoid accusations of breaking the law [7,8]. The structure of SCs has been dynamically developing, but all of them follow certain principles and can be broadly dissected into a parent nuclear group, a neck link, a head group, and a tail side chain [9]. These four parts are crossed and combined to form different types of SCs. SCs mainly work by combining with cannabinoid receptors [10], and their pharmacological effect is stronger than that of natural cannabinoids [11]. After abuse, the common adverse reactions include anxiety, numbness, epilepsy, hallucinations, slurred speech, decreased consciousness level, excitement, aggressiveness, vomiting, dizziness, and increased blood pressure, which have greatly endangered human health [12,13]. The whole class of SCs has been regulated in China since 1 July 2021, which is the first batch of regulated new psychoactive substances [14,15].

As synthetic cannabinoids undergo rapid metabolism after entering the human body, drug prototypes can hardly be detected in the body, and the detection method for drug prototypes can easily lead to missed detection of such drugs by mistake [16,17], resulting in false-negative results, and thus metabolites need to be studied. Countries around the world attach great importance to the problem of SCs abuse and actively carry out relevant research. Mogler et al. used pooled human liver microsomal incubation sample analysis and real urine sample analysis to identify the stage I metabolites of 5F-MDMB-PICA and found that the ester hydrolysis metabolites were the most abundant [18]. Walle et al. found that in vitro (pooled human liver S9, human liver microsomes, and porcine liver microsomes) and in vivo (rat and pig) systems, the azaindole derivative CUMYL-5F-P7ICA underwent oxidative defluorination, monohydroxylation, ketone formation, and carboxylation as the most common Phase I reactions and detected the formation of sulfated and glucuronidated Phase II metabolites [19]. Although domestic and foreign scholars have conducted in-depth studies on the in vivo or in vitro metabolism of individual synthetic cannabinoids, these studies only include the metabolism and pharmacological action analysis of a few individual synthetic cannabinoids circulating in the market [20,21,22,23,24,25,26,27,28,29,30,31,32], and there is no systematic classification study to cope with the forensic identification of potential and constantly updated synthetic cannabinoids. Therefore, it is necessary to classify and summarize SCs, study the metabolic mechanisms of different SCs, and confirm the metabolic laws of these substances by inferring the metabolic processes of different SCs so as to provide technical support for the metabolism research of potential new SCs.

The aim of this study was to identify the metabolites from 26 SCs in human liver microsomes (HLM) and in rat experiments and to compare these results with those obtained from closely related analogs using an identical experimental setup. We conducted a thorough analysis of the metabolic pathways and rules governing SCs metabolism. The combination of in vitro and in vivo models was intended to provide complementary and/or supportive data. The samples were analyzed using liquid chromatography (LC)–high-resolution mass spectrometry, which enabled the differentiation of various metabolites with the same nominal mass but different exact masses. The results could provide an identification method and serve as a prediction screening tool for monitoring and controlling SCs.

## 2. Results and Discussion

### 2.1. Somatic Structure and Mass Spectrometric Fragmentation Pattern of SCs

**Figure 1:**
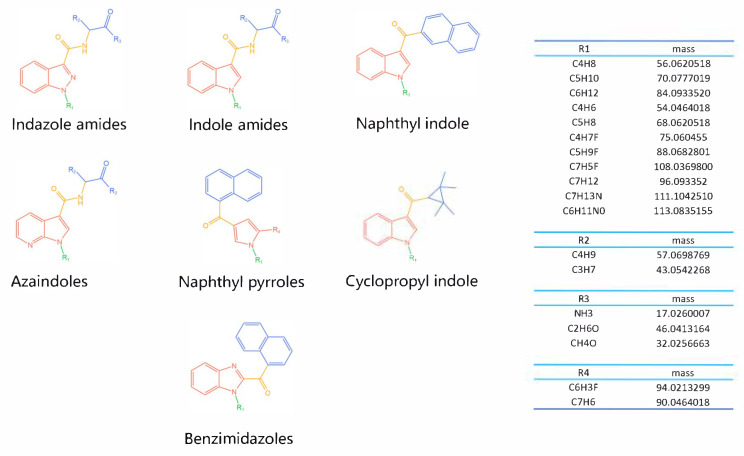
Structures of the 7 categories of synthetic cannabinoids: Red is the parent nucleus structure, orange is the neck link, blue is the head group, and green is the tail side chain; R represents substituents at different sites, and R4 and the pyrrole ring are connected to form the parent nucleus structure of o-pyrrole.

**Figure 2:**
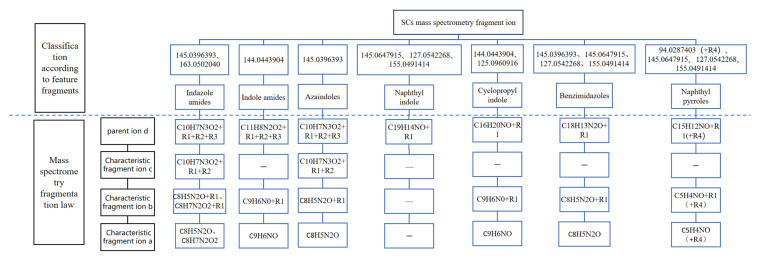
Fragmentation pattern of SCs precursor mass spectrum. “—”means that such substances do not have such characteristic fragment ions. The upper part of the figure shows the characteristic fragment ions unique to each type of SCs, which can be used for preliminary classification; the lower part of the figure illustrates the fragmentation patterns of the parent bodies of each type of SCs.

The 26 SCs were classified according to the structure of four parts into indole amide, indazole amide, azaindole amide, naphthyl indole, cyclopropylindole, naphthyl benzimidazole, and naphthyl pyrrole (o-pyrrole is also included in this category), as shown in Figure 1 (the specific structures of 26 types of SCs are shown in Table 1). The product ion spectra and fragmentation of various SCs are shown in Supplementary Materials—Figure S1. After the protonated molecular ions were formed in the mass spectrum of indazolamide-based SCs, the terminal amino group or ester group, carbonyl group, neck link, and tail side chain were gradually fragmented to obtain four characteristic fragment ions. The latter two are prone to amide hydrolysis, resulting in the formation of six characteristic fragment ions. Among them, the characteristic fragment ion 145.0396393 obtained by finally removing the tail side chain and its hydrolysate 163.050240 were the core structures of indazolamide-based SCs. After forming protonated molecular ions in the mass spectrum, indole amide-based SCs gradually broke the neck link and the tail side chain and obtained two characteristic fragment ions, which were less than those of indazole amide-based SCs, and generally did not undergo hydrolysis. Finally, the characteristic fragment ion 144.0443904 obtained by removing the tail side chain is the core structure of indole amide-type SCs. The fragmentation pattern of azaindole amide-based SCs was similar to that of indazole amide-based SCs. Four characteristic fragment ions were formed after the terminal amino group or ester group, carbonyl group, neck link, and tail side chain were gradually broken, and the characteristic fragment ion obtained by removing the tail side chain at last was 145.0396393. The core structure of azaindole amide-based SCs. Generally, the naphthyl indole-like SCs only had three naphthyl-related characteristic fragment ions, namely, a naphthyl plus formyl fragment ion 145.0647915, a naphthyl fragment ion 127.0542268, and a naphthyl hydrolyzed fragment ion 155.0491414, which were formed after the neck link was broken, all of which were the characteristic structures of the naphthyl indole-like SCs. Cyclopropyl indole-type SCs were similar to indole amide-type SCs. After protonated molecular ions were formed in the mass spectrum, the neck link and the tail side chain were gradually broken to obtain two characteristic fragment ions. In addition, the characteristic fragment ions of cyclopropyl plus formyl at the head were formed from different fracture positions at the neck. The characteristic fragment ion 144.0443904 and the characteristic fragment ion of cyclopropyl plus formyl 125.096096 obtained by removing the tail side chain were the characteristic structures of cyclopropyl indole-type SCs. After forming protonated molecular ions in a mass spectrum, the naphthyl benzimidazole SCs gradually break the neck link and the tail side chain to obtain two characteristic fragment ions, which are easy to hydrolyze and form four characteristic fragment ions in total, wherein the characteristic fragment ion 145.0396393 obtained by removing the tail side chain at last is the core structure of the naphthyl benzimidazole SCs, and the characteristic fragment ion 273.1022396 can also be formed by removing the tail side chain at first. In addition, naphthyl benzimidazole SCs can form 145.0647915, 127.0542268, and 155.0491414 naphthyl-related characteristic fragment ions like naphthyl indole SCs. After forming protonated molecular ions in the mass spectrum, the naphthyl pyrrole SCs gradually fragmented the neck link and the tail side chain to obtain two characteristic fragment ions, of which the characteristic fragment ion obtained by finally removing the tail side chain was the core structure of the naphthyl pyrrole SCs, and in addition, three naphthyl-related characteristic fragment ions of 145.0647915, 127.0542268, and 155.0491414 could be formed. The fragment structure information is shown in Figure 2. In addition, AB-FUBINACA, EMB-FUBINACA, and AMB-FUBICA were different from other indazoles or indole amide SCs in that the ionization of the three was terminated after the neck link was broken and the tail side chain was not broken, which was deduced from the fact that the benzene ring of the tail substituent was relatively stable and it was closely connected with the parent nucleus group so that it was not easy to be broken.

Based on the MS fragmentation patterns of precursors of various SCs, the protonated molecular ions, also known as parent ions, were designated with the letter ‘d’. The characteristic fragment ions resulting from the removal of the terminal amino or ester group were labeled as ‘c’. Meanwhile, the fragment ions formed by the elimination of both the head group and the tail side chain, along with their hydrolysis products, were represented by the letters ‘b’ and ‘a’, respectively. A tissue structure diagram was developed to illustrate and understand the MS fragmentation rules of SC precursors, as shown in Figure 2.

### 2.2. Conversion Rate of Human Liver Microsomal Incubation

Human liver microsome incubation serves as a critical preclinical tool for modeling Phase I drug metabolism and assessing metabolic stability. By comparing the peak area of the prodrug in the incubated experimental group to that in the degradation control group, the conversion rate of the prodrug can be ascertained. As shown in Table 2, the indole amide-type SCs and naphthyl benzimidazole-type SCs exhibit higher conversion rates, both exceeding 80%. Conversely, the conversion rates of indazole amide-type SCs display significant variation, with ADB-4en-PINACA demonstrating the lowest rate at only 18.2%. In contrast, 5F-EMB-PINACA, AB-4en-PINACA, ADB-3en-BUTINACA, and EMB-FUBINACA exhibit the most optimal conversion rates, all surpassing 95%. These findings indicate that SC drugs undergo rapid metabolism, resulting in a limited window for detecting their precursors. At the same time, they validate the successful establishment of an in vitro human liver microsomal incubation system in this study, which could provide a certain theoretical basis for the research on novel drug metabolites within the forensic science domain.

### 2.3. Clinical Symptoms of Rats in Metabolic Model

Animal manifestations after SC drug injection include gait instability, convulsion, tachypnea and irritability, decreased body temperature, listlessness and even syncope, and hematuria after partial drug injection. This was demonstrated by the syncope in rats given 5F-CYPPICA and JWH-370. Rats injected with ADB-CHMINACA, EMB-FUBINACA, and 5F-EMB-PINACA were irritable, with unstable gait and tachypnea. Rats injected with AMB-FUBICA, FUB-144, 5F-MBMB-PINACA, and 5F-EMB-PINACA had hematuria. For the rats in the other experimental groups, most of them maintained a depressed state and ate independently for about 10 min. Rats in the blank control group showed mild behaviors and normal behaviors after receiving a normal saline injection.

After entering the body, the synthetic cannabinoid drugs tightly combine with plasma proteins and quickly enter various tissues and organs in the body and specifically combine with cannabinoid receptors to exert toxicological effects. Cannabinoid receptors are G protein-coupled receptors with two major subtypes: cannabinoid receptor type 1 (CB1R) and cannabinoid receptor type 2 (CB2R). The CB1R receptor has a certain regulatory effect and strong stimulation on the excitability and inhibitory effect of dopaminergic neurons in the cerebral cortex pathway. Drugs that bind to the receptor interfere with hippocampal synaptic transmission, widely affect physiological functions (such as cardiovascular, gastrointestinal, and urinary systems), and have strong hallucinogenic, tranquilizing, and inhibitory effects on the central system [11]. Therefore, after drug injection, reactions such as gait instability, convulsion, tachypnea, irritability, decreased body temperature, listlessness, syncope, and hematuria may occur. In addition, the combination of synthetic cannabinoid drugs with CB1R and CB2R produces the typical quadruple effects in rodents, namely, analgesia, hypothermia, motor inhibition, and immobilization [8]. 

### 2.4. Metabolite Analysis

In this study, we developed both in vitro and in vivo metabolic models for 26 SCs, encompassing four indoleamides, eleven indazolamides, one azaindole, two naphthyl indoles, three cyclopropylindoles, two naphthyl benzimidazoles, and three naphthyl pyrroles. These SCs represent the most prevalent types with high detection rates in recent years, each possessing unique structural features. We systematically identified the metabolites of these 26 SCs, resulting in a comprehensive list of 609 metabolites, including 420 Phase I metabolites and 189 Phase II metabolites, across 30 metabolic pathways. Notably, 458 metabolites were newly identified compared with the previous studies [20,21,22,23,24,25,26,27,28,29,30,31,32,33,34,35,36,37,38]. The measurement accuracy of these metabolites was within 5 × 10^−6^, and crucially, no parent compounds or metabolites from any SCs were detected in the blank control group. To prioritize forensically relevant biomarkers, we employed rigorous screening criteria based on four key aspects: i. shared metabolites between in vitro and in vivo models; ii. metabolites generated at SCs-specific structural motifs (e.g., dihydrodiol formation at olefin side chain and ester hydrolysis products following ester group cleavage in the head group); and iii. structurally unique metabolites distinguishing individual SCs. The information on various SCs progenitors, the count of metabolites, and the characteristic metabolites is summarized in Table 1. Detailed metabolite identification workflows are demonstrated using 5F-MDMB-PICA and 5F-EMB-PINACA as representative examples. Parallel analytical procedures were applied to the remaining SCs, with full spectral data and fragmentation patterns provided in Supplementary Materials—Table S1 and Supplementary Materials—Figure S2.

#### 2.4.1. Metabolite Profiling of 5F-MDMB-PICA

Following human liver microsomal incubation, fourteen metabolites were detected, including twelve Phase I and two Phase II metabolites. These metabolites were involved in eight metabolic pathways, encompassing deamidation, amide hydrolysis, dealkylation, dehydrogenation, hydroxylation, oxidative defluorination, ester hydrolysis, and glucuronidation. In the rat metabolic model, twelve metabolites were identified, with seven Phase I metabolites and five Phase II metabolites. These metabolites were associated with seven metabolic pathways, including acidification, dehydrogenation, hydroxylation, oxidative defluorination, dihydrodiol reaction, sulfation, and glycoside binding. The measurement errors of all 25 metabolites across both models remained within 5 × 10^−6^.

**Figure 3:**
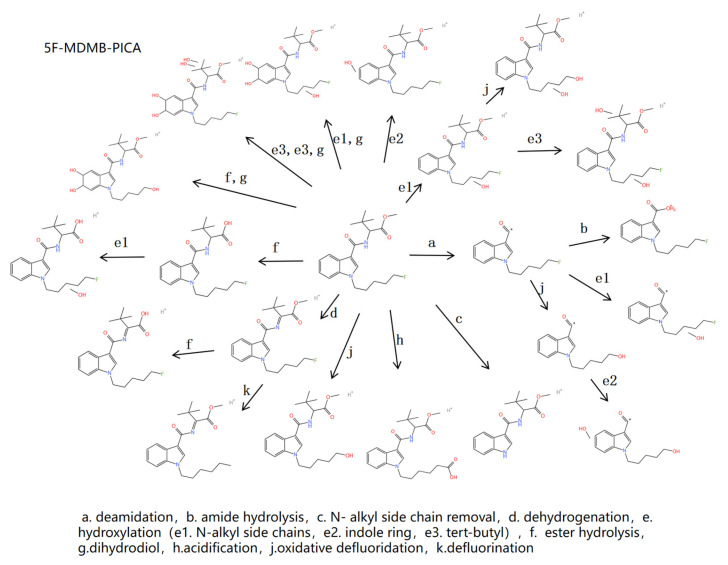
Metabolic response diagram for 5F-MDMB-PICA.

Table 3 information for parent and metabolites of 5F-MDMB-PICA. Figure 3 is a metabolic map of 5F-MDMB-PICA.

L2 has an *m*/*z* of 232.11276, and the mass difference is 145 Da compared with that of the parent drug. Moreover, the characteristic fragment ion *m*/*z* 144.04414 is consistent with that of the parent drug, so it can be deduced that L2 is caused by the deamination of the dimethyl butyryl methyl ester group, that is, the removal of the head group of the parent drug. The *m*/*z* of L1 is 230.11729, and *m*/*z* for fragment ions are reduced by 2 Da for L2 compared to L1, presumably due to oxidative defluorination of L1. L3 has an *m*/*z* of 246.97934, +16 Da compared to L1, and characteristic fragment ions *m*/*z* of 161.05960 and 233.58668, suggesting that the hydroxylation site occurred on the indazole ring. L4 has an m/z of 248.10809, +16 Da compared to L2, and characteristic fragment ions *m*/*z* of 161.05961 and 233.58666, suggesting that L4 is a metabolite of L2 hydroxylation on the indazole ring.

L5 has an *m*/*z* of 250.12396, which is 127 Da less than the parent drug and 18 Da more than L2, thus inferring that L5 is a metabolite resulting from amide hydrolysis of the parent drug.

L6, with an *m*/*z* of 289.15466, is a de-N-alkyl metabolite formed by removing the pentanyl side chain from the N atom on the indazole ring of the parent drug. The characteristic fragment ion does not form the characteristic fragment ion with an N-alkyl side chain 232.57333 compared with the parent drug.

L8 has an *m*/*z* of 363.20784, which is −14 Da compared to the parent drug but consistent with the fragment ions, suggesting that L8 is an ester hydrolysis metabolite. L10 has an m/z of 379.22274, +16 Da compared to L8, and characteristic fragment ions *m*/*z* of 161.05960 and 233.58668, suggesting that the hydroxylation site occurred on the indazole ring. L7 has an *m*/*z* of 361.19219, which is 2 Da less than L8, and the other characteristic fragment ions are consistent with L8, suggesting that a dehydrogenation reaction occurred.

The fluoroalkyl group is formed by connecting halogen fluorine at the end of an alkyl side chain, and the C-F bond is easily broken to defluorinate. The mass number of metabolites is −18 Da(−F) compared with that of the parent compound. And after defluorination, the metabolites are oxidized into alcohol, namely, oxidized defluorinated metabolites, with the mass number of −2 Da(−F+O+H) compared with that of the parent drug. The *m*/*z* of L9 is 375.22709, which was −2 Da compared with that of the parent drug, but the fragment ions were consistent, so it could be deduced that L9 was an oxidative defluorination metabolite. L9 was further oxidized to an acid, the acidified metabolite L11, at an *m*/*z* of 389.20617, relative to the parent drug −12 Da by mass. L12 has an *m*/*z* of 391.22165, which is 16 Da more than that of L9. It is deduced that L12 is formed by hydroxylation of L9, with the characteristic fragment ions *m*/*z* of 144.04404 and 248.66737. It can be deduced that the hydroxylation site occurs on the N-alkyl side chain. The *m*/*z* of L15 is 409.23453, representing an increase of 34 Da in mass number compared with L9 and the increase of 34 Da in mass number of fragment ions compared with L9. It could be deduced that L15 was a dihydrodiol metabolite of L9.

The mass numbers of L13 and L14 are similar, *m*/*z* is 393.21861 and 393.21867, and that mass number is increased by 18 Da compared with the parent drug, but the characteristic fragment ions of L13 are different from the two; the characteristic fragment ions of L13 are 160.05960 and 248.58668, compared with the characteristic fragment ions of 144.04424 and 232.11288 of the parent drug. A mass shift of 16 Da occurred from the parent nucleus structure. It was deduced that the hydroxylation site occurred on the indole ring. The characteristic fragment ions of L14 were 144.04425 and 248.58688, and the fragment ion 144.04425 representing the linkage of the parent nucleus structure with the neck was unchanged. It indicated that the fragment ion representing the linkage of the parent nucleus structure with the neck and the N-alkyl side chain underwent mass shift, indicating that the hydroxylation site occurred on the N-alkyl side chain. The *m*/*z* of L16 was 427.22397, with an increase in mass of 34 Da compared with that of L14 and an increase in mass of both fragment ions by 34 Da compared with that of L14. It can be deduced that L16 is a metabolite of L14 in the dihydrodiol reaction. L17 had an *m*/*z* of 443.21719, a 16 Da increase in mass relative to L16, and fragment ions consistent with only 178.0480741 relative to L16, suggesting that L17 was a dihydroxylated and dihydrodiol metabolite with the hydroxylation site occurring on t-butyl.

L25 has an *m*/*z* of 569.25097, and compared to L14 mass +176 Da, the characteristic fragment ions were consistent with L14, identifying L25 as the Phase II metabolite of L14 combined with glucuronide.

The glucuronic acid reaction was formed by the combination of the original drug and glucuronic acid group, with the *m*/*z* of L23 being 553.25525 and +176 Da compared with the parent drug. The characteristic fragment ions were consistent with the parent drug, suggesting that the glucuronic acid reaction occurred.

The sulfation reaction was formed by the combination of the parent drug and sulfate radical. The *m*/*z* of L20 is 455.16275, which was +78 Da compared with the parent drug. The characteristic fragment ions were consistent with the parent drug, suggesting that sulfation and dehydrogenation reactions occurred. L19 has an *m*/*z* of 453.16676, which is −2 Da by mass compared to L20, but consistent with the fragment ions, suggesting that L19 is an oxidative defluorination metabolite of L20. L21 +112 Da compared to the parent drug, suggesting that sulfation and dihydroxylation occurred, with characteristic fragment ions consistent with the parent drug, and that the hydroxylation site occurred on the N-alkyl side chain.

The glycosidic binding reaction was formed by the combination of the parent drug and glycosidic bond. The *m*/*z* value of L22 is 539.27594, which was +162 Da compared with the parent drug. The characteristic fragment ions were consistent with the parent drug, suggesting that the glycosidic binding reaction occurred. L24 is +16 Da compared to L22, with consistent characteristic fragment ions, suggesting hydroxylation on the N-alkyl side chain.

From the above results, we observed that the predominant Phase I metabolic reactions in human liver microsomal incubations involved ester hydrolysis and hydroxylation, whereas glucuronidation emerged as the most prevalent Phase II metabolic reaction. Conversely, in the rat metabolic model, the key Phase I metabolic reactions encompassed oxidative defluorination and hydroxylation, with sulfation and glycoside binding reactions being the most significant Phase II metabolic processes. The oxidative defluorination metabolite was a common outcome in both models. The structural features of 5F-MDMB-PICA include an ester group at the head and fluorine substitution at the tail, and the de-N-alkyl side chain metabolite can be distinguished from the metabolites of other SCs and exhibits a substantial peak area. In conclusion, the ester hydrolysis, de-N-alkylation, and oxidative defluorination metabolites were identified as the defining metabolites of 5F-MDMB-PICA.

#### 2.4.2. Metabolite Profiling of 5F-EMB-PINACA

Following human liver microsomal incubation, eleven metabolites were detected, including 10 Phase I and one Phase II metabolite. These metabolites were involved in 10 metabolic pathways, encompassing deamidation, amide hydrolysis, acidification, demethylation, dehydrogenation, hydroxylation, ketone formation, oxidative defluorination, ester hydrolysis, dihydrodiol reaction, and glucuronidation. In the rat metabolic model, 13 metabolites were identified, with 10 Phase I metabolites and 3 Phase II metabolites. These metabolites were associated with eight metabolic pathways, including deamidation, ester hydrolysis, ketone formation, hydroxylation, oxidative defluorination, dihydrodiol reaction, sulfation, and arginine binding. The measurement errors of all 23 metabolites across both models remained within 5 × 10^−6^.

**Figure 4:**
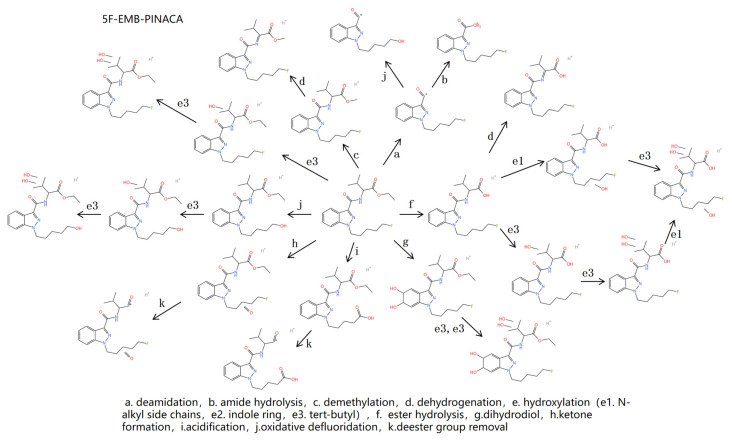
Metabolic response diagram of 5F-EMB-PINACA.

Table 4 information for parent compound and metabolites of 5F-EMB-PINACA. Figure 4 is a metabolic profile of 5F-EMB-PINACA.

B2 has an *m*/*z* of 233.10822, a mass number difference of 145 Da compared with that of the parent drug, and the characteristic fragment ions of 145.03920 and 163.05035 are consistent with that of the parent drug. It can be deduced that B1 is caused by the deamination of the dimethyl butyryl methyl ester group, that is, the removal of the head group of the parent drug. B2 has an *m*/*z* of 231.11260, and that of B2 was 2 Da less than that of B1, presumably due to oxidative defluorination of B1. B3 has an *m*/*z* of 251.11884, which is 127 Da less than the parent and 18 Da more than B2, so B3 is presumed to be a metabolite resulting from amide hydrolysis of the parent drug.

B5 has an *m*/*z* of 350.18716, which was −14 Da compared to the parent drug, but the fragment ions were consistent, suggesting that B5 was an ester hydrolysis metabolite. B4 has an *m*/*z* of 348.17145, which is 2 Da less than B5, and the other characteristic fragment ions are consistent with B5, suggesting that dehydrogenation occurred at the amide linkage. B8 and B9 have similar mass numbers, with *m*/*z* of 366.18201 and 366.18219, which are 18 Da higher than that of B5, but their characteristic fragment ions are different. The characteristic fragment ions of B8 are 145.03920, 163.05035, 249.10310, and 267.11368. The fragment ions 145.03920 and 163.05035 representing the linkage of the parent structure to the neck remained unchanged, indicating that the fragment ion representing the linkage of the parent structure to the neck plus the N-alkyl side chain underwent mass shift, suggesting that the hydroxylation site occurred on the N-alkyl side chain. The characteristic fragment ions for B9 are 145.03920, 163.05035, 233.10825, and 251.11879, and the fragment ion is consistent with B5, suggesting that the hydroxylation site occurs on t-butyl. The *m*/*z* of B11 is 382.17831, which was 16 Da more than that of B9. The hydroxylation reaction was deduced, and the characteristic fragment ion of B11 was consistent with that of B9, indicating that the hydroxylation site occurred on the tert-butyl group. B21 has an *m*/*z* of 526.21936, +176 Da compared to B5, and characteristic fragment ions consistent with B5, suggesting that a glucuronic acid reaction occurred.

B7 has an *m*/*z* of 364.20267, a mass of −14 Da compared to the parent drug, but consistent fragment ions, suggesting that B8 was a demethylated metabolite. B6 has an m/z of 362.18704, which is 2 Da less than B7, and the other characteristic fragment ions are consistent with B7, suggesting that a dehydrogenation reaction occurred.

The *m*/*z* of B10 is 376.22266, which was −2 Da compared to the parent drug, but the fragment ions remained consistent, suggesting that B10 was an oxidative defluorination metabolite. B10 is further oxidized to acid, the acidified metabolite B12, with a *m*/*z* of 390.20187, relative to the parent drug −12 Da. B13 has an *m*/*z* of 394.21341, an increase of 16 Da compared to B10, which is deduced to be formed by hydroxylation of B10, with characteristic fragment ions *m*/*z* of 145.03921, 163.05034, 233.10824, and 251.11879, and the hydroxylation site can be deduced to occur on t-butyl. The *m*/*z* of B15 is 408.21231, which was 16 Da more than that of B13. It was deduced that B15 was formed by hydroxylation of B9. The characteristic fragment ions were consistent with those of B13. It could be deduced that the hydroxylation site also occurred on the tert-butyl group.

The *m*/*z* of B14 is 394.21341, and the characteristic fragment ions are 145.03957, 163.05009, 233.10836, 251.11887, 320.16677, and 348.17010. The fragment ions 233 and 251, representing the linkage of the parent nucleus structure to the neck and the N-alkyl side chain, have not changed, and the subsequent fragment ions have undergone mass shift, suggesting that the hydroxylation site occurred on t-butyl. B16 has an *m*/*z* of 410.20978, an increase of 16 Da compared to B14, and is presumed to be formed by hydroxylation of B14 with characteristic fragment ions 145.03957, 163.05009, 233.10836, and 251.11887, consistent with B14, and it can be deduced that the hydroxylation site also occurs on t-butyl. The *m*/*z* of B19 is 444.21551, which indicates that the mass number of B19 is increased by 34 Da compared with that of B16, and the mass number of fragment ions is increased by 34 Da compared with that of B16. It can be deduced that B17 is formed by the dihydrodiol reaction of B16. B18 has an *m*/*z* of 426.20187, an increase of 16 Da compared to B16, and is presumed to be formed by hydroxylation of B16 with characteristic fragment ions of 145.03957, 163.05009, 249.10311, and 267.11369, suggesting that the hydroxylation site occurs in the N-alkyl side chain.

The *m*/*z* of B17 was 412.22226, which indicated that the mass number of B17 was increased by 34 Da compared with that of the parent drug, and the mass number of fragment ions was increased by 34 Da compared with that of the parent drug. It could be deduced that B17 was a dihydrodiol metabolite.

The sulfation reaction was formed by the combination of the parent drug and sulfate radical. The *m*/*z* of B20 was 472.1567, which was +94 Da compared with the parent drug. The characteristic fragment ions were consistent with the parent drug, suggesting that sulfation and ketone formation occurred.

The arginine binding reaction was formed by the combination of the parent drug and arginine. The m/z of B22 was 534.31790, which was +156 Da compared with the parent drug. The characteristic fragment ions were consistent with the parent drug, suggesting that the arginine binding reaction occurred. B23 compared to B24 +14 Da, suggesting that a ketogenic reaction occurred.

From the above results, we observed that the predominant Phase I metabolic reactions in human liver microsomal incubations involved ester hydrolysis and hydroxylation, whereas glucuronidation emerged as the most prevalent Phase II metabolic reaction. Conversely, in the rat metabolic model, the key Phase I metabolic reactions encompassed oxidative defluorination and hydroxylation, with sulfation and arginine binding reactions being the most significant Phase II metabolic processes. The ester hydrolysis metabolite was a common outcome in both models. The characteristic structural features of 5F-EMB-PINACA include an ethyl group at the head and fluorine substitution at the tail, and the ester hydrolysis and hydroxylated metabolite can be distinguished from the metabolites of other SCs and exhibit a substantial peak area. In conclusion, the ester hydrolysis, ester hydrolysis, hydroxylated, and oxidative defluorination metabolites were identified as the defining metabolites of 5F-EMB-PINACA.

### 2.5. Metabolic Pathway Analysis

A total of 30 metabolic pathways were involved in both human liver microsomal incubation and rat metabolic models. Phase I metabolic reactions included a diverse array of processes such as deamination, transesterification, hydrolysis (including ester hydrolysis), decarbonylation, dehydrogenation, cyclopropyl group cleavage, deamidation (including amide hydrolysis), formyl group removal, deamidation, N-alkyl and N-phenyl side chain cleavage, desethyl morpholine oxidation, demethylation, hydration, acidification, ketone formation, defluorination, oxidation defluorination, hydroxylation, dihydroxylation, and dihydrodiol reaction. Phase II reactions, however, primarily involved glucuronidation, glycosidic binding, sulfation, acetylation, and the conjugation with various amino acids, including arginine and dihydrodiol reactions.

Deamination and de-esterification occur on SCs featuring acyclic head side chains. Specifically, deamination typically involves the cleavage of the terminal amino group from head-like methylbutyramide and dimethylbutyramide, resulting in the breakdown of the C-N bond. This produces a metabolite with a mass reduction of 17.0260007 Da (−NH3) compared to the parent compound (all the following values are theoretical). The de-esterification usually entails the cleavage of the terminal ester group from compounds such as methyl butyrate or ethyl butyrate, causing the breakdown of the C-O bond. This results in metabolites that are lighter by 32.0256663 Da (−CH4O) or 46.0413164 Da (−C2H6O) compared to the parent compound.

Hydrolysis occurs when the amino or ester groups at the termini of methylbutyramide, dimethylbutyramide, methyl butyrate, and ethyl butyrate undergo cleavage. Hydrolysis (amino hydrolysis) specifically results in metabolites with a mass increase of 0.9840156 Da (+O−N−H) relative to the parent drug. In the case of ethyl ester, hydrolysis leads to a metabolite with a mass reduction of 28.0307517 Da (−C2H4) compared to the parent drug. For methyl ester, hydrolysis results in a metabolite that is lighter by 14.0151017 Da (−CH2) than the parent drug.

Decarbonylation also occurs on SCs featuring acyclic head side chains, where the C-C bond linking to the carbonyl group is cleaved. The resulting mass difference between the metabolite and its parent compound hinges on the mass of the terminal ester group or amino group. Dehydrogenation, however, readily occurs at the C-N bond connecting the tert-butyl group and the amide group, leading to the formation of a C=N bond. It can also occur at the tail side chain and the cyclopropyl group, giving rise to a C=C bond. The specific site of dehydrogenation depends on where the characteristic fragment ions of the metabolite commence their mass shift relative to those of the parent compound. The metabolite exhibits a mass loss of 2.0151017 Da (−2H) when compared with the parent compound. The dehydrogenation site depends on the position from which the mass displacement of the characteristic fragment ion of the metabolite began compared with that of the parent substance. If the characteristic fragment ion ‘a’ remains consistent with the parent substance, while the ions ‘b’, ‘c’, and ‘d’ exhibit an increase of 2.0151017 Da. It indicated that the dehydrogenation reaction occurred within the tail side chain. When the characteristic fragment ions ‘a’ and ‘b’ were consistent with the precursor, while both ‘c’ and ‘d’ showed an increase of 2.0151017 Da, dehydrogenation occurred at the amide group. However, when the characteristic fragment ions ‘a’ and ‘b’ were consistent with the precursor, and ‘d’ demonstrated an increase of 2.0151017 Da, the dehydrogenation reaction occurred on the cyclopropyl group.

The cyclopropyl group is present in the head side chain of 5F-CYPPICA following the cleavage of the C-N bond that connects the cyclopropyl group to the neck amide group. This results in metabolite N8, which exhibits a mass difference of 54.0464018 Da (−C4H6) relative to parent compound N0. Additionally, the characteristic fragment ion at 144.04425Da is consistent with the parent compound.

Deamidation, amide hydrolysis, deformylation, and other modifications took place at the cervical junction of the SCs. Deamidation is formed by breaking a C-N bond on a neck amide group, while a deformyl group is formed by breaking a C-C bond on a neck formyl group; both modifications involve the removal of head groups from SCs. The mass differences between the resulting metabolites and their parent compounds depend on the mass of the respective head group. Amide hydrolysis is the hydrolysis of the amide group subsequent to deamidation, which leads to a metabolite with a mass increase of 18.0100163 Da (+H2O) compared to the deamidated metabolite. Similarly, the mass difference from the parent compound also depends on the mass of the head group involved.

The N-alkyl side chains and N-phenyl side chains involve the elimination of the tail side chains from SCs, evident through the cleavage of the N-C bond linking the parent nuclear group to the tail side chain. The resultant difference in mass number, when compared to the parent compound, corresponds to the mass of the removed tail side chains.

The desethyl morpholine and demethylation reactions serve to eliminate portions of the tail side chain of SC. The desethyl morpholine occurs within the tail side chain of JWH-200, resulting from the cleavage of the C-C bond at the ethyl morpholine-alkyl juncture. Compared with the parent compound, this leads to a mass difference of 85.0522154 Da (−C4H7NO), with characteristic fragment ions that are consistent. Following the desethyl morpholine cleavage, further oxidation reactions may take place, yielding metabolites with a mass difference of 69.0573008 Da (−C4H7N) relative to the parent compound. Moreover, demethylation occurs within the caudal side chain of AB-005, involving the cleavage of the N-C bond in methylpiperidinyl. This results in a mass reduction of 14.0151017 Da (−CH2) compared to the parent compound, accompanied by characteristic fragment ions that remain consistent.

The acidification reaction typically occurs on alkyl, alkenyl, or fluoroalkyl groups at the terminus of the tail side chain, predominantly involving butyrization or pentylation. When alkyl side chains undergo oxidation to form acids, the metabolites exhibit a mass increase of 29.9741791 Da (+2O−2H). The alkenyl side chain was prone to hydration reaction, where the C=C bond was cleaved to form an alcohol, leading to an increase of 18.0100163 Da (+2H+O) compared to the parent compound. Subsequent oxidation of these hydrated metabolites resulted in acids, with a mass increase of 31.9892808 Da (+2O) relative to the parent compound.

The fluoroalkyl groups are formed by connecting halogen fluorine at the end of an alkyl side chain. These groups are susceptible to C-F bond cleavage and defluorination, resulting in metabolites with a mass decrease of 18.9978548 Da (−F) compared with the parent compound. Following defluorination, the metabolites are further oxidized to form alcohol, known as oxidized defluorinated metabolites, with a mass change of 1.9956636 Da (−F+O+H) relative to the parent compound. These oxidized defluorinated metabolites can be further oxidized to form acids, referred to as acidified metabolites, with a mass change of 11.983601 Da (−F+2O-H).

The ketogenic reaction also takes place on the alkyl, alkenyl, or fluoroalkyl groups at the terminal end of the tail side chain. The hydrogen atom attached to the carbon group within the alkyl moiety undergoes deoxygenation, resulting in the formation of a C=O double bond. This transformation leads to a mass increase of 13.9792645 Da (+O−2H) when compared to the parent compound.

Hydroxylation is a prevalent reaction in drug metabolism and also constitutes the most common metabolic pathway for the synthesis of cannabinoids [39,40]. It occurs at various sites, including the parent nuclear structure (such as the indole ring and indazole ring), the head side chain (tert-butyl and naphthyl groups, etc.), and the tail side chain (alkyl, alkenyl, fluoroalkyl, etc.). Hydroxylation can also occur at multiple sites concurrently, resulting in dihydroxylation and trihydroxylation reactions. Compared to the parent compound, the mass of the metabolites is increased by n × 15.9943662 Da (n × O). The specific location of hydroxylation depended on the starting position of mass displacement in the characteristic fragment ions of the metabolites relative to the parent compound. When the mass of characteristic fragment ions ‘a’, ‘b’, ‘c’, and ‘d’ was increased by 15.9943662 Da, it indicated the presence of a hydroxylation site within the parent nucleus structure. If the characteristic fragment ion ‘a’ remained unchanged while the masses of ions ‘b’, ‘c’, and ‘d’ were increased by 15.9943662 Da, the hydroxylation site was located in the tail side chain. If the characteristic fragment ions ‘a’ and ‘b’ were consistent with the precursor, while the fragment ions ‘c’ and ‘d’ showed an increase of 15.9943662 Da, the hydroxylation site existed in the head group.

The dihydrodiol reaction usually occurs on the alkenyl side chain of the parent nucleus structure and naphthyl group at the head. In mammalian drug metabolism, a common mechanism involves the epoxidation (+O) of the double bond, followed by hydrolysis (+H2O) to form the corresponding trans-dihydrodiol [39,41]. This results in a metabolite with a mass increase of 34.0049309 Da (+2O+2H) compared with the parent compound. When the characteristic fragment ions ‘a’, ‘b’, ‘c’, and ‘d’ exhibited an increase in mass of 34.0049309 Da compared with the precursor, it indicated that the dihydrodiol reaction occurred on the parent nucleus structure. Furthermore, the presence of the characteristic fragment ion at 189.0540723 Da indicates that the dihydrodiol reaction has occurred on the naphthyl group.

In contrast, Phase II reactions involve binding reactions, where the original drug or a Phase I metabolite combines with a corresponding group. The resulting fragment ions are largely consistent with the reaction precursor. Glucuronidation is the most significant and prevalent mode of Phase II binding. In liver microsome incubation, glucuronosyltransferase enzymes transfer glucuronosyl groups to SCs, forming glucuronides, which have a mass number that is 176.0315394 Da (+C6H8O6) greater than the parent compound. The sulfation reaction involved the conjugation of the original drug with a sulfate group (+SO3), resulting in a mass increase of 79.9562661 Da. Acetylation occurs when the original drug combines with an acetyl group, leading to an increase in the mass number by 42.0100163 Da (+C2H2O). The glycosidic bond was formed when the original drug combined with a glycosidic moiety, resulting in a mass increase of 162.0522749 Da (+C6H10O5). The in vivo amino acid binding reactions involving arginine, ornithine, glycine, and glutamine exhibit respective mass increases of 156.1005626 Da, 114.0787645 Da, 57.0209153 Da, and 128.0580290 Da following ligand conjugation.

### 2.6. Metabolism Analysis of SCs

**Figure 5:**
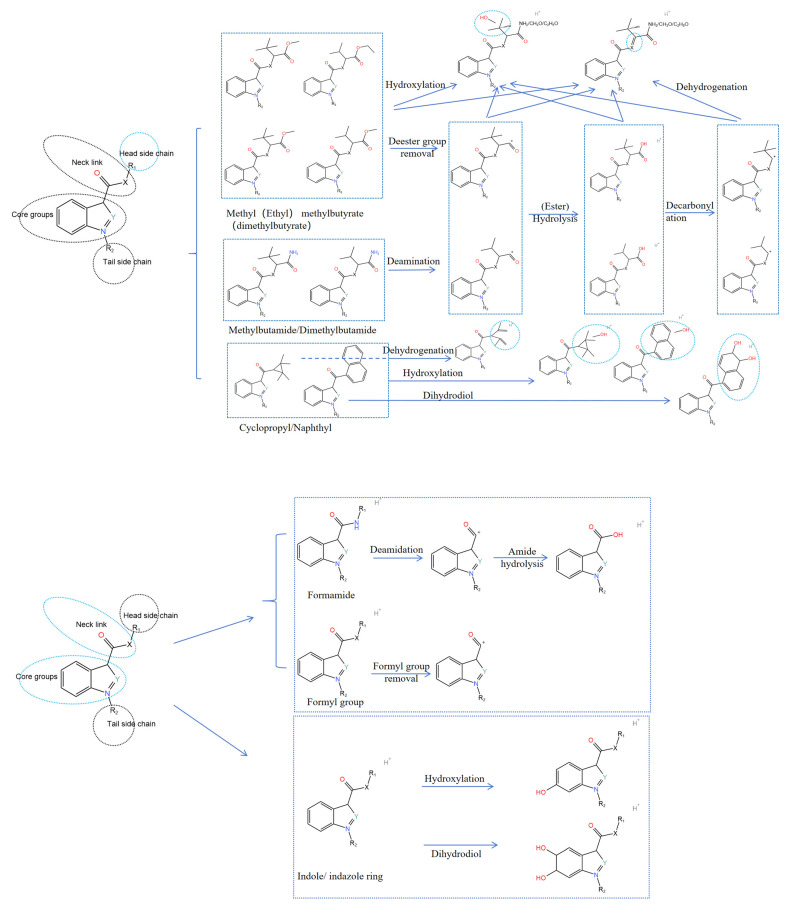

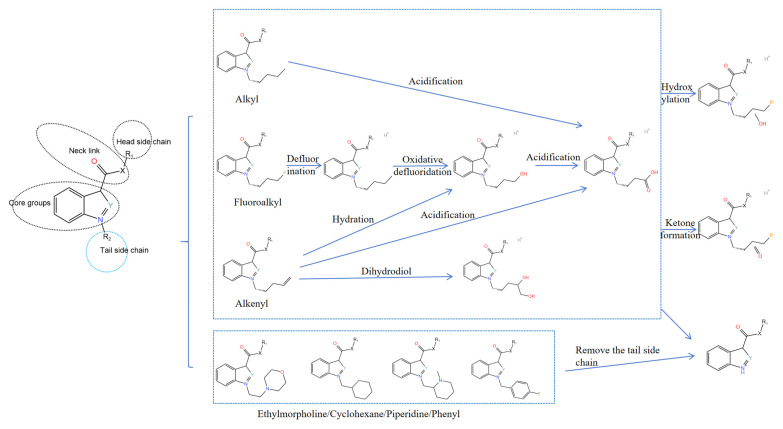
Summary of SCs metabolism.

The metabolism of SCs is predominantly governed by their structural composition [12]. SCs are universally constructed from a parent nuclear group, neck linkage, head group, and tail side chain, with each component undergoing distinct reaction processes. Figure 5 provides a comprehensive overview of SCs’ metabolism.

The parent nucleus of SCs typically possesses cyclic structures, including indole, indazole, pyrrole, and imidazole, which readily undergo oxidative metabolism to form hydroxylated metabolites. Indole and indazole rings are susceptible to dihydrodiol reactions.

When considering the linking moiety (or neck structure) of SCs, the linking moieties of indole formamide and indole formamide SCs were formamide, which was prone to deamidation and further hydrolyzed to form amide hydrolysis metabolite. However, SCs derived from naphthyl indoles, naphthyl pyrroles, benzimidazoles, and cyclopropylindoles possess a formyl group as their linking moiety, which will occur through formylation.

The head group is prone to the formation of esters such as dimethyl methyl butyrate, methyl methyl methyl butyrate, and ethyl methyl butyrate, which are susceptible to ester group cleavage and hydrolysis, ultimately yielding decarbonylation metabolites. Additionally, the methyl structure is easily oxidized to produce hydroxylated metabolites. The termini of methylbutyramide and dimethylbutyramide possess amino groups, and compared to C-C bonds, the C-N bonds are more susceptible to cleavage and hydrolysis. Consequently, the terminal amino groups often undergo hydrolysis, followed by carbonyl cleavage, to form a decarbonylation metabolite. Furthermore, the C-N bond linking the tert-butyl group and the amide group is susceptible to dehydrogenation, resulting in the formation of C=N bonds. Lastly, the naphthyl group, characterized by its cyclic aromatic structure, is prone to undergoing hydroxylation and dihydrodiol reactions.

The side chains at the tail end comprise alkyl, alkenyl, and tolyl groups. Typically, the tail end undergoes halogen substitution, most commonly with fluorine. The alkyl and alkenyl groups are susceptible to hydroxylation, acidification, or direct cleavage. The double bonds at the tail end of the alkenyl side chain are easily epoxidized, followed by a hydration reaction to produce dihydrodiol. The tolyl group, with its stable structure, usually undergoes direct cleavage. As for the halogen substitution at the tail end, it predominantly involves fluorine, which is subject to oxidative defluorination and further oxidation to generate acidified metabolites.

### 2.7. Spectrum Fragmentation Pattern of SCs Metabolites

SCs drugs and their metabolites exhibit obvious characteristic fragment ions in mass spectrum. The cleavage sequence of the precursor typically involves the initial removal of the terminal amino or ester group, followed by the detachment of the head group, and culminating in the shedding of the tail side chain. The masses of these fragment ions display a predictable pattern, characterized by a fixed mass shift relative to the precursor at the site of reaction, as shown in Table 5. The table summarizes the fragmentation patterns observed for metabolites resulting from a single-step reaction. Notably, metabolites arising from two-step and three-step reactions exhibit mass shift based on both the original drug precursor and intermediate one-step reaction metabolite, although these are not detailed in the table. In addition, SCs with a naphthalene group as the head group adhere to these fragmentation rules while also generating a specific fragment ion of 155.0491414 (C11H7O), corresponding to the naphthalene and formyl group, upon breakage of the neck linker. When reactions occur on the naphthalene group, corresponding mass shifts are observed, providing valuable insights into determining the site of reaction. These characteristic fragment ions are instrumental in facilitating the structural identification or confirmation of the sample.

### 2.8. Comparison of Human Liver Microsomal Incubation and Rat Metabolic Model

In the human liver microsomal incubation, a total of 383 metabolites were identified, consisting of 337 Phase I metabolites and 46 Phase II metabolites. These metabolites are associated with 24 distinct metabolic pathways. Phase I metabolic reactions encompass a variety of processes such as deamination, de-esterification, hydrolysis, ester hydrolysis, decarboxylation, dehydrogenation, acylation, deamidation, amide hydrolysis, deformylation, removal of N-alkyl and N-phenyl side chains, desethylmorpholine formation, demethylation, hydration, acidification, ketone formation, defluorination, oxidative defluorination, hydroxylation, dihydroxylation, and the dihydrodiol reaction. Conversely, Phase II reactions primarily involve glucuronidation. In the rat metabolic model, a total of 316 metabolites were identified, comprising 172 Phase I metabolites and 144 Phase II metabolites, spanning across 27 metabolic pathways. Phase I metabolic reactions include processes like deamination, de-esterification, hydrolysis, ester hydrolysis, dehydrogenation, deamidation, amide hydrolysis, deformylation, removal of N-alkyl and N-phenyl side chains, hydration, acidification, ketone formation, oxidative defluorination, hydroxylation, dihydroxylation, and dihydrodiol reaction. Phase II reactions, however, predominantly involve glucuronidation, glycosylation, sulfation, acetylation, and conjugation with various amino acids, including arginine.

The human liver microsomal incubation serves as an effective simulator of human metabolic processes, providing a faster and more comprehensive metabolite profiling approach than animal experiments. However, the primary metabolites identified following incubation in this model were predominantly Phase I metabolites, with Phase II reactions being limited to glucuronidation and, occasionally, acetylation. This model is unable to produce the sulfated metabolites and various amino acid-binding metabolites observed in the rat metabolic model. The possible reasons for the speculation are as follows: i. During the preparation of human liver microsomes, some aldehyde oxidases and glutathione S-transferases, which are essential for human metabolism, are removed due to their larger mass during the differential centrifugation process, leading to fewer Phase II reactions in the model, which does not fully reflect the drug metabolism process in the human body; ii. Due to species differences, the degree of sulfation or sulfonation in rats is higher than in other species but hardly ever occurs in humans or pigs [42,43]. Furthermore, the drug content in the experimental results may not align with the actual samples, rendering these results unsuitable for direct application in real sample analysis. In this study, the rat exposure model was established for in vivo metabolism research. Given the high genetic similarity between rats and humans, rats serve as an excellent simulator of in vivo metabolism. The metabolic pathways of Phase I metabolites in both models share considerable overlap. Notably, the rat metabolic model produced fewer Phase I metabolites compared to the human liver microsomal incubation, with hydroxylation and dihydrodiol metabolites being most prevalent. Due to their high polarity, these metabolites are more readily eliminated from the body. In addition, the number of Phase I metabolic pathways in the rat metabolic model was reduced, and specific pathways such as decarbonylation, cyclopropylation, defluorination, and desethylmorpholine were absent. These pathways are elimination reactions, potentially due to excessive reactions following drug introduction and inadequate sampling time points. Models in vivo and in vitro rarely have the same metabolites, possibly for the following reasons: i. In vitro model: Liver microsomes do not contain cell membranes, so drugs can directly contact the metabolic enzymes in liver microsomes without needing to pass through the cell membrane, resulting in a higher biotransformation rate of drugs in liver microsomes compared to primary hepatocytes; ii. During the preparation of human liver microsomes, some aldehyde oxidases and glutathione S-transferases, which are essential for human metabolism, are removed due to their larger molecular mass. This results in fewer Phase II conjunction reactions in the model, which fails to fully recapitulate the complexity of in vivo metabolism; iii. During in vivo metabolism, there is a stronger tendency for conversion into highly polar metabolites for excretion.

Considering the inherent differences between the rat and human samples, a combined approach was employed. The in vitro incubation technology of human liver microsomes was utilized to simulate the human metabolic process, while in vivo metabolism research technology involving exposed rats provided additional insights. This comprehensive analysis allowed for a detailed examination of the metabolic pathways, mechanisms, and rules governing synthetic cannabinoids.

## 3. Materials and Methods

### 3.1. Chemicals and Reagents

JWH-019 (99.7%), UR-144 (99.8%), 5F-EMB-PICA (99.3%), AMB-FUBICA (99.91%), FUB-144 (99.7%), 5F-ADB (99.7%), AB-FUBINACA (99.8%), ADB-4en-PINACA (99.8%), EDMB-PINACA (99.9%), ADB-CHMINACA (100.0%), BIM-018 (99.8%), JWH-030 (99.8%), FUBIMIN (99.9%), EMB-FUBINACA (99.8%), ADB-P7AICA (99.8%), 5F-EMB-PINACA (99.8%), AB-4en-PINACA (99.6%), JWH-307 (99.5%), ADB-3en-BUTINACA (99.6%), ADB-HEXINACA (99.9%), 5F-CYPPICA (99.8%), 5F-MDMB-PICA (99.5%), JWH-370 (98.9%), and MDMB-4en-PINACA (99.6%) were purchased from Shanghai Yuansi Technology and Shanghai Academy of Criminal Sciences; AB-005 (99.9%) and JWH-200 (99.6%) were purchased from China Pharmaceutical University. Syringe filters (0.45 μm), acetonitrile, and methanol of chromatographic purity were purchased from Sigma-Aldrich Trading Co., Ltd. (Shanghai, China). Chromatographic purity of ammonium formate and formic acid were purchased from Merck KGaA (Darmstadt, Germany). In vitro metabolism research kits were purchased from Beijing Huizhi Heyuan Biotechnology Co., Ltd. (Beijing, China); the kit contains the following: human liver microsomes (20 mg/mL), NADPH regeneration solution A and solution B, and 0.1 mol/L phosphate buffer solution (the kit is stored at −80 °C). Ultrapure water was produced in the laboratory.

### 3.2. Human Liver Microsomal Incubation

Each of the 26 SCs was configured to 1 mg/mL with acetonitrile. The HLMs, NADPH regeneration solution A, regeneration solution B, and UDPGA solution were thawed at 4 °C. An icebox was prepared, and all additional steps were performed on ice. For this, 10 µL of NADPH regeneration solution A, 2 µL of regeneration solution B, 2 µL of SCs standard, and 10 µL of mixed human liver microsomes were added to 176 µL of 0.1 mol/L phosphate buffer. The mixture was mixed thoroughly and incubated at 37 °C for 1 h. Subsequently, 20 µL of UDPGA solution was added and mixed well, followed by an additional incubation for 30 min. The reaction was stopped by adding 200 µL of ice-cold acetonitrile. Additionally, the negative control group and the degradation control group were set up following the same procedure. The negative control group did not include SC standard, while the degradation control group did not include mixed human liver microsomes. The unadded components were supplemented with an equal amount of phosphate buffer. All samples were centrifuged at a high speed of 13,500 r for 10 min after reaction termination. The supernatant was collected and subsequently passed through a membrane filter (0.45 μm) prior to sampling.

### 3.3. Rat Metabolic Model

Sprague-Dawley male rats (200 ± 20 g) were obtained from SPF (Beijing) Biotechnology Co., Ltd. (Beijing, China). The animals were housed under controlled environmental conditions: Temperature was maintained at 20–25 °C, humidity at 50 ± 10%, and a 12/12 h light/dark cycle was applied. They had ad libitum access to standard laboratory chow and filtered water. A two-week acclimatization period was provided before the experiments began. The 26 SC standards were prepared in normal saline at concentrations of 0.5 mg/mL, 0.1 mg/mL, and 0.05 mg/mL. Each rat was weighed before administration, and the injection dose was adjusted based on body weight. The dosing volume for each rat was maintained at approximately 200 µL.

162 Sprague-Dawley (SD) rats were randomly divided into 27 groups, with six rats in each group. The control group received an injection of normal saline, while the remaining groups were injected with 26 different types of SCs. The dosage administered was determined based on pre-experimental results, specifically selecting the dose that produced the highest level of metabolites in the blood samples collected 1 h after tail vein injection at concentrations of 0.5 mg/kg, 0.1 mg/kg, and 0.05 mg/kg, as shown in Table 6. Subsequently, tail vein administration was conducted at the specified dosage. The orbital venous blood was taken from the outer canthus of the rat with capillaries at 15 min and 1 h. Feces were collected after the blood. The rats were then sacrificed, and their brains and livers were dissected. Samples were immediately frozen in liquid nitrogen and stored at −80 °C until analysis.

Sample pretreatment:

For blood samples, 0.5 mL of each sample was mixed with 1.0 mL of acetonitrile. For brain samples, 0.2 g of tissue was combined with 0.5 mL of acetonitrile. For liver samples, 0.5 g of tissue was mixed with 0.5 mL of acetonitrile. For feces samples, 0.2 g of sample was combined with 1 mL of acetonitrile-water (3:1) for grinding and homogenization. Each mixture was sonicated for 40 min, allowed to precipitate at 4 °C for 30 min, and then centrifuged at 13,500 rpm for 10 min. The supernatant was filtered using a syringe filter (0.45 μm) for further testing.

### 3.4. LC–Orbitrap Analysis

The samples were separated and analyzed using a UHPLC-Q-Exactive Orbitrap MS system (Thermo Fisher Scientific, Waltham, MA, USA), which comprised a Thermo-Scientific™ Q-Exactive™(Thermo Fisher Scientific, Waltham, MA, USA) quadrupole electrostatic field orbitrap mass spectrometer coupled to a Vanquish ultrahigh-performance liquid chromatography (UHPLC) system (Thermo Fisher Scientific, Waltham, MA, USA) with a heated electrospray ionization HESI source in the positive ionization mode. The samples were separated on an ACQUITY UPLC BEH C18 Column (100 × 2.1 mm, 1.7 μm) with the column temperature maintained at 30 °C. The chromatographic separation was achieved using a gradient elution program with mobile phase A consisting of 0.1% formic acid in water and mobile phase B containing 0.1% formic acid in acetonitrile. The gradient profile was programmed as follows: Initial conditions were maintained at 5% B from 0 to 1 min, followed by a linear increase to 95% B from 1 to 11 min; the 95% B composition was held from 11 to 15.5 min, then decreased back to 5% B from 15.5 to 16 min, and finally maintained at 5% B from 16 to 18 min. The flow rate was set at 0.3 mL/min, and the injection volume was 10 μL for all analyses.

The following HESI source conditions were adopted: Acquisition mode was set to positive ion mode; the capillary temperature was maintained at 325 °C, the auxiliary gas heater temperature was set to 350 °C, and the spray voltage was applied at 3.7 kV. The mass spectrometry analysis was carried out in full-scan (FS) mode to trigger data-dependent acquisition in tandem mass spectrometry (FS-ddMS^2^) mode. The FS data acquisition was performed using the following parameters: a resolution of 60,000, an automatic gain control (AGC) target of 1 × 10^6^, a scan range of *m*/*z* 66.7–1000, and a maximum injection time (IT) of 250 ms. The ddMS^2^ data acquisition was performed using the following parameters: a resolution of 30,000; an automatic gain control (AGC) target of 1 × 10^5^; normalized collision energies (NCEs) of 20, 40, and 70 eV; and a maximum injection time (IT) of 50 ms.

### 3.5. Data Processing

Data acquisition was executed utilizing Xcalibur 4.3 software (Thermo Fisher Scientific, Waltham, MA, USA). The subsequent analysis of the acquired data was conducted using Thermo Fisher Scientific Composite Discoverer 3.2 software (Thermo Fisher Scientific, Waltham, MA, USA). The workflow’s nodes employed specific parameters: a mass tolerance of 5 ppm, a signal-to-noise threshold of 3, a minimum peak intensity of 1 × 10^5^, a maximum peak width of 0.8 min, and a minimum of 3 scans per peak. The metabolites were evaluated based on the following criteria: an average peak area exceeding 1 × 10^6^, a mass error of less than 5 ppm for the protonated molecule, a consistent isotopic pattern, a product ion spectrum that aligns with the proposed structure, and a plausible retention time for the proposed structure. Notably, no identical peaks were observed in the negative control and degradation samples. After the screening of metabolites, mass spectrometric analysis was performed on the metabolites generated in the metabolic model. The reaction conversion rates were quantitatively determined through comparative analysis of peak areas between the experimental group and degradation control group for each synthetic cannabinoid compound. Furthermore, the metabolites of 26 types of SCs were individually identified. By observing the relationship between the fragment ions of metabolites and comparing the differences between the various fragment ions, we determined which elements have increased or decreased in the metabolites compared to the parent. From this comparison, we inferred the metabolic reactions that occurred and the metabolic sites.

## 4. Conclusions

In this study, a UHPLC-QE Orbitrap MS detection method was used to analyze the prodrugs, metabolites, and fragmentation patterns of seven SCs in both an in vivo rat metabolism model and an in vitro liver microsomes metabolism model. A total of 609 metabolites were identified, comprising 420 Phase I metabolites and 189 Phase II metabolites. There were 30 metabolic pathways involved in total. Phase I metabolic reactions included deamination, deesterification, hydrolysis, ester hydrolysis, decarbonylation, dehydrogenation, cyclopropyl group cleavage, deamidation, amide hydrolysis, formyl group removal, N-alkyl side chain removal, N-phenyl side chain elimination, desethyl morpholine removal, demethylation, hydration, acidification, ketone formation, defluorination, oxidative defluorination, hydroxylation, dihydroxylation, trihydroxylation, and dihydrodiol reaction. Meanwhile, Phase II reactions mainly included glucuronic acid conjugation. For the in vitro model, Phase I metabolic reactions included ester hydrolysis, dehydrogenation, deamidation, amide hydrolysis, formyl group removal, N-alkyl side chain removal, demethylation, hydration, acidification, ketone formation, oxidative defluorination, hydroxylation, and dihydrodiol formation. Phase II reactions were mainly arginine conjugation, with acetylation, sulfation, glycoside conjugation, ornithine conjugation, and glutamine conjugation reactions also occurring. In addition, this study summarized the metabolic rules and mass spectrometry fragmentation patterns of various synthetic cannabinoids, categorizing them based on their head group, neck linker, tail side chain, and core group in the studied species. This study summarizes the metabolic patterns of seven types of SCs and the m/z variation patterns of their metabolites relative to the parent compounds. In the future, a visual feature matrix can be established to predict the metabolites and metabolic patterns of new types of SCs. Additionally, an online mass spectrometry data stream processing pipeline can be established for dynamic monitoring and early warning.

In vitro liver microsomal metabolism offers advantages including low cost, operational simplicity, and easy control of experimental conditions. However, this approach has inherent limitations and may not fully replicate the authentic metabolic profiles of synthetic cannabinoids in humans. Rat models are frequently employed in pharmacological studies due to their organ structures and metabolic rates being relatively comparable to those in humans, enabling simulation of drug absorption, distribution, metabolism, and excretion (ADME) processes. While rats provide physiologically relevant data approximating human responses, interspecies variations in metabolic pathways necessitate caution in extrapolating results. To address these limitations, we adopted a complementary approach combining both in vitro and in vivo metabolic models. This integrated strategy enhances reliability by cross-validating findings between experimental systems. The metabolite data and metabolic markers obtained from this study can be applied to the detection of actual cases, providing a foundation for identifying such substances in biological samples and serving as a reference for further studies on the metabolic mechanism of other novel SCs.

## Figures and Tables

**Table 1:** In the prosome information, metabolite count, and characteristic metabolite structures of all kinds of SCs; red was the parent nucleus structure, orange was the neck link, blue was the head group, and green was the tail side chain.

Type	Number	Name	Structure	Structural Formula	Number of Metabolites	Number of Phase I Metabolites	Number of Phase II Metabolites	Characteristic Metabolite
Indazolamide	A	AB-FUBINACA	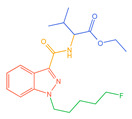	C_20_H_21_FN_4_O_2_	19	15	4	Hydrolysis, hydroxylation, and deamination metabolites
	B	5F-EMB-PINACA	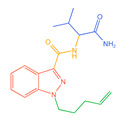	C_20_H_28_FN_3_O_3_	23	19	4	Ester hydrolysis, oxidative defluorination, ester hydrolysis, and hydroxylated metabolites
	C	AB-4en-PINACA	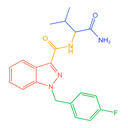	C_18_H_24_N_4_O_2_	18	17	1	Deamination, hydration, and deamidation metabolites
	D	ADB-4en-PINACA	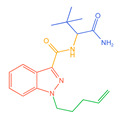	C_19_H_26_N_4_O_2_	30	25	5	Deamination, hydration, dihydrodiol, and deamination metabolites
	E	ADB-CHMINACA	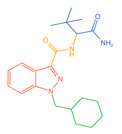	C_21_H_30_N_4_O_2_	25	18	7	Deamination, deamination of N-alkyl side chains, and hydrolytic metabolites
	F	ADB-HEXINACA	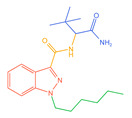	C_20_H_30_N_4_O_2_	31	24	7	Deamination, deamidation, amide hydrolysis, and hydroxylation metabolites
	G	EDMB-PINACA	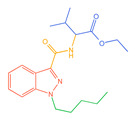	C_21_H_31_N_3_O_3_	42	31	11	Ester hydrolysis, ketogenic, and de-N-alkyl side chain metabolites
	H	EMB-FUBINACA	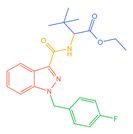	C_22_H_24_FN_3_O_3_	14	9	5	Ester hydrolysis, amide hydrolysis, ester hydrolysis, and hydroxylated metabolites
	I	ADB-3en-BUTINACA	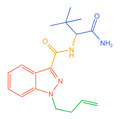	C_18_H_24_N_4_O_2_	22	18	4	Dihydrodiol, deamination, deamination, and hydroxylation metabolites
	J	5F-ADB	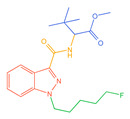	C_20_H_28_FN_3_O_3_	25	17	8	Ester hydrolysis, dehydrogenation, and acidification metabolites
	K	MDMB-4en-PINACA	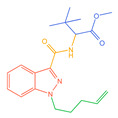	C_20_H_27_N_3_O_3_	14	12	2	Dihydrodiol, ester hydrolysis, ketogenic, and amide hydrolysis metabolites
Indoleamides	L	5F-MDMB-PICA	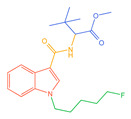	C_21_H_29_FN_2_O_3_	25	18	7	Ester hydrolysis, de-N-alkyl side chain, and oxidative defluorination metabolite
	M	5F-EMB-PICA	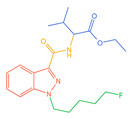	C_21_H_29_FN_2_O_3_	19	9	10	Ester hydrolysis, amide hydrolysis, and oxidative defluorination metabolite
	N	5F-CYPPICA	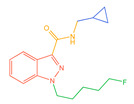	C_18_H_23_FN_2_O	25	22	3	Polycyclic butane, de-N-alkyl side chains, and dehydrometabolites
	O	AMB-FUBICA	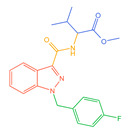	C_22_H_23_FN_2_O_3_	25	15	10	Ester hydrolysis, deamidation, and de-N-alkyl side chain metabolites
Azaindoles	P	ADB-P7AICA	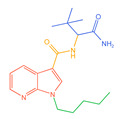	C_19_H_28_N_4_O_2_	40	28	12	Hydroxylation, deamination, and dehydrogenation metabolites
Naphthyl indoles	Q	JWH-019	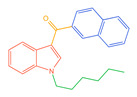	C_25_H_25_NO	27	16	11	Dihydrodiol, hydroxylated, and ketogenic metabolite
	R	JWH-200	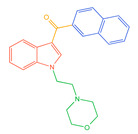	C_25_H_24_N_2_O_2_	16	11	5	Dihydrodiol, desethylmorpholine, and hydroxylate metabolites
Naphthyl benzimidazoles	S	BIM-2201	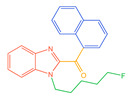	C_23_H_21_FN_2_O	29	17	12	Oxidative defluorination, dihydrodiol, and hydroxylated metabolite
	T	BIM-018	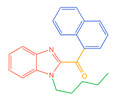	C_23_H_22_N_2_O	27	13	14	Ketogenic, hydroxylated, and dehydrogenated metabolites
Cyclopropylindoles	U	UR-144	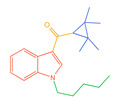	C_21_H_29_NO	24	15	9	Hydroxylation, de-N-alkyl side chains, and ketogenic metabolites
	V	AB-005	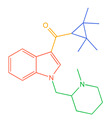	C_23_H_32_N_2_O	17	12	5	Hydroxylation, de-N-alkyl side chain, dehydrogenation, and dihydroxylation metabolites
	W	FUB-144	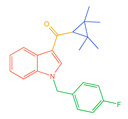	C_23_H_24_FNO	18	11	7	Ketogenic and hydroxylated, hydroxylated, and dehydrogenated metabolites
Naphthyl pyrroles	X	JWH-030	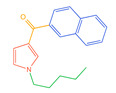	C_20_H_21_NO	12	7	13	Dihydrodiol, hydrate, and hydroxylated metabolite
	Y	JWH-307	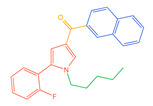	C_26_H_24_FNO	20	12	8	Hydroxylation, ketogenic, and de-N-alkyl side chain metabolites
	Z	JWH-370	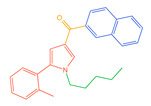	C_27_H_27_NO	14	9	5	Dihydrodiol, hydroxylated, and de-N-alkyl side chain metabolites

**Table 2:** Conversion rate of in vitro model method.

Classify	Abbreviation	Conversion Rate
Indoleamides	5F-CYPPICA	80.4%
	5F-MDMB-PICA	95.9%
	5F-EMB-PICA	80.6%
	AMB-FUBICA	87.9%
Azaindoles	ADB-P7AICA	42.9%
Indazolamide	AB-4en-PINACA	98.7%
	AB-FUBINACA	74.6%
	ADB-3en-BUTINACA	98.7%
	ADB-4en-PINACA	18.2%
	ADB-CHMINACA	62.8%
	ADB-HEXINACA	24.8%
	EDMB-PINACA	71.3%
	EMB-FUBINACA	99.8%
	5F-EMB-PINACA	100.0%
	5F-ADB	88.1%
	MDMB-4en-PINACA	69.7%
Naphthyl benzimidazoles	FUBIMINA,BIM-2201	90.2%
	BIM-018	93.7%
Naphthyl pyrroles	JWH-030	99.8%
	JWH-307	55.2%
	JWH-370	42.0%
Cyclopropylindoles	AB-005	60.9%
	FUB-144	30.4%
	UR-144	30.5%
Naphthyl indoles	JWH-019	84.7%
	JWH-200	67.4%

**Table 3:** 5F-MDMB-PICA parent compound and metabolite information.

Name	Formula	Transformations	Composition Change	In Vitro Metabolites			In Vivo Metabolites		
				Annot. DeltaMass [ppm]	*m*/*z*	RT [min]	Annot. DeltaMass [ppm]	*m*/*z*	RT [min]
L0	C_21_H_29_FN_2_O_3_	5F-MDMB-PICA		0.64	377.22374	9.37			
L1	C_14_H_15_NO_2_	Deamidation + Oxidative defluoridation	−(C7 H14 F N O)	0.73	230.11772	7.96			
L2	C_14_H_14_FNO	Deamidation	−(C7 H15 N O2)	0.8	232.1134	8.34			
L3	C_14_H_15_NO_3_	Deamidation + Oxidative defluoridation + Hydroxylation	−(C7 H14 F N)	0.8	246.11267	6.87			
L4	C_14_H_14_FNO_2_	Deamidation + Hydroxylation	−(C7 H15 N O)	−0.16	248.10809	7.96			
L5	C_14_H_16_FNO_2_	Amide hydrolysis	−(C7 H13 N O)	0.72	250.12396	8.34			
L6	C_16_H_20_N_2_O_3_	N-alkyl side chain removal	−(C5 H9 F)	0.5	289.15482	7.63			
L7	C_20_H_25_FN_2_O_3_	Ester hydrolysis + Dehydrogenation	−(C H4)	−4.14	361.19239	8.31			
L8	C_20_H_27_FN_2_O_3_	Ester hydrolysis	−(C H2)	0.39	363.20798	8.34			
L9	C_21_H_30_N_2_O_4_	Oxidative defluoridation	−(F) +(H O)	0.36	375.22797	7.97	−1.98	375.22709	6.73
L10	C_20_H_30_N_2_O_5_	Ester hydrolysis + Hydroxylation	−(C H2) +(O)	0.23	379.2027485	7.33			
L11	C_21_H_28_N_2_O_5_	Acidification	−(H F) +(O2)				−2.39	389.20617	8.04
L12	C_21_H_30_N_2_O_5_	Oxidative defluoridation + Hydroxylation	−(F) +(H O2)				−2.82	391.22165	7.59
L13	C_21_H_29_FN_2_O_4_	Hydroxylation	+(O)	0.49	393.21861	9.56			
L14	C_21_H_29_FN_2_O_4_	Hydroxylation	+(O)	0.32	393.21887	8.49			
L15	C_21_H_32_N_2_O_6_	Oxidative defluoridation + Dihydrodiol	−(F) +(H3 O3)				2.97	409.23453	10.22
L16	C_21_H_31_FN_2_O_6_	Hydroxylation + Dihydrodiol	+(H2 O3)				0.18	427.22397	8.77
L17	C_21_H_31_FN_2_O_7_	Dihydroxylation + Dihydrodiol	+(H2 O4)				−3.66	443.21719	5.67
L18	C_21_H_33_FN_2_O_7_	Dihydrodiol + Dihydrodiol	+(H4 O4)				−3.96	445.2327	5.43
L19	C_21_H_28_N_2_O_7_S	Oxidative defluoridation + Dehydrogenation + Sulfation	−(H F) +(O4 S)				−4.95	453.16676	8.85
L20	C_21_H_27_FN_2_O_6_S	Dehydrogenation + Sulfation	−(H2) +(O3 S)				−4.21	455.16275	8.83
L21	C_21_H_29_FN_2_O_8_S	Dihydroxylation + Sulfation	+(O5 S)				4.11	489.17215	1.45
L22	C_27_H_39_FN_2_O_8_	Glycoside	+(C6 H10 O5)				−0.71	539.27594	10.76
L23	C_27_H_37_FN_2_O_9_	Glucuronidation	+(C6 H8 O6)	0.61	553.25525	11.97			
L24	C_27_H_39_FN_2_O_9_	Hydroxylation + Glycoside	+(C6 H10 O6)				−4.73	555.26862	10.91
L25	C_27_H_37_FN_2_O_10_	Hydroxylation + Glucuronidation	+(C6 H8 O7)	0.83	569.25097	6.87			

**Table 4:** 5F-EMB-PINACA parent compound and metabolite information.

Name	Formula	Transformations	Composition Change	In Vitro Metabolites			In Vivo Metabolites		
				Annot. DeltaMass [ppm]	*m*/*z*	RT [min]	Annot. DeltaMass [ppm]	*m*/*z*	RT [min]
B0	C_20_H_28_FN_3_O_3_	5F-EMB-PINACA			378.21793	10.11			
B1	C_13_H_14_N_2_O_2_	Deamidation + Oxidative defluoridation	−(C7 H14 F N O)				−0.89	231.1126	4.42
B2	C_13_H_13_FN_2_O	Deamidation	−(C7 H15 N O2)	−1.09	233.10822	8.35			
B3	C_13_H_15_FN_2_O_2_	Amide hydrolysis	−(C7 H13 N O)	−0.79	251.11884	8.35			
B4	C_18_H_22_FN_3_O_3_	Dehydrogenation + Ester hydrolysis	−(C2 H6)	−1	348.17145	8.17			
B5	C_18_H_24_FN_3_O_3_	Ester hydrolysis	−(C2 H4)	−0.81	350.18716	7.20	−2.25	350.18666	8.42
B6	C_19_H_24_FN_3_O_3_	Demethylation + Dehydrogenation	−(C H4)	−1.12	362.18704	8.12			
B7	C_19_H_26_FN_3_O_3_	Demethylation	−(C H2)	−1.18	364.20267	9.49			
B8	C_18_H_24_FN_3_O_4_	Ester hydrolysis + Hydroxylation(N-alkyl side chains)	−(C2 H4) +(O)	−0.97	366.18201	7.56			
B9	C_18_H_24_FN_3_O_4_	Ester hydrolysis + Hydroxylation(Tert-butyl)	−(C2 H4) +(O)	−0.47	366.18219	6.83			
B10	C_20_H_29_N_3_O_4_	Oxidative defluoridation	−(F) +(H O)				−1.14	376.22266	4.99
B11	C_18_H_24_FN_3_O_5_	Ester hydrolysis + Dihydroxylation	−(C2 H4) +(O2)				2.72	382.17831	1.14
B12	C_20_H_27_N_3_O_5_	Acidification	−(H F) +(O2)	−1.22	390.20187	8.29			
B13	C_20_H_29_N_3_O_5_	Oxidative defluoridation + Hydroxylation	−(F) +(H O2)				−1.29	392.21749	4.10
B14	C_20_H_28_FN_3_O_4_	Hydroxylation(Tert−butyl)	+(O)	−0.64	394.21341	8.57			
B15	C_20_H_29_N_3_O_6_	Oxidative defluoridation + Dihydroxylation	−(F) +(H O3)				−1.48	408.21231	4.06
B16	C_20_H_28_FN_3_O_5_	Dihydroxylation	+(O2)				2.94	410.20978	5.31
B17	C_20_H_30_FN_3_O_5_	Dihydrodiol	+(H2 O2)				−4.78	412.22226	5.48
B18	C_20_H_28_FN_3_O_6_	Trihydroxylation	+(O3)				−3.8	426.20187	4.84
B19	C_20_H_30_FN_3_O_7_	Dihydrodiol + Dihydroxylation	+(H2 O4)				3.29	444.21551	6.58
B20	C_20_H_26_FN_3_O_7_S	Ketone formation + Sulfation	−(H2) +(O4 S)				3.98	472.1567	1.03
B21	C_24_H_32_FN_3_O_9_	Ester hydrolysis + Glucuronidation	+(C4 H4 O6)	−0.33	526.21936	7.21			
B22	C_26_H_40_FN_7_O_4_	Arginine binding	+(C6 H12 N4 O)				−3.66	534.3179	8.37
B23	C_26_H_38_FN_7_O_5_	Ketone formation + Arginine binding	+(C6 H10 N4 O2)				−2.99	548.29749	10.19

**Table 5:** Summary of SCs metabolite characteristic fragment ions. ”—”means that such substances do not have such characteristic fragment ions.

Primitive Body	Hydroxylation (on Parent Nucleus)	Dihydroxylation (on Parent Nucleus)	Dihydrodiol Reaction (on Parent Nucleus)	Defluorination to Acid	Ketone Formation	Hydroxylation (on N-alkyl Side Chain)	Hydration	Defluorination	Alkyl Acidification	Alkenylation or Dihydroxylation (on that N-alkyl Side Chain)	Dihydrodiol Reaction (N-alkyl Side Chain)
Characteristic fragment ion a	a+O	a+2O	a+2H+2O	a	a	a	a	a	a	a	a
Characteristic fragment ion b	b+O	b+2O	b+2H+2O	b−H−F+2O	b−2H+O	b+O	b+2H+O	b−F+H	b−2H+2O	b+2O	b+2H+2O
Characteristic fragment ion c	c+O	c+2O	c+2H+2O	c−H−F+2O	c−2H+O	c+O	c+2H+O	c−F+H	c−2H+2O	c+2O	c+2H+2O
Parent ion d	d+O	d+2O	d+2H+2O	d−H−F+2O	d−2H+O	d+O	d+2H+O	d−F+H	d−2H+2O	d+2O	d+2H+2O
**Primitive body**	**Dehydrogenation (on tail side chain)**	**Dehydrogenation (amide junction)**	**Hydroxylation (on tert-butyl)**	**Dihydroxylation (on tert-butyl)**	**Methyl ester hydrolysis**	**Ethyl ester hydrolysis**	**Amino hydrolysis**	**De-N-** **alkyl side chain**	**Hydroxylation (on naphthyl)**	**Dihydroxylation (on naphthyl)**	**Dihydrodiol reaction (on naphthyl)**
Characteristic fragment ion a	a	a	a	a	a	a	a	a	C11H7O+O	C11H7O+2O	C11H7O+2H+2O
Characteristic fragment ion b	b−2H	b	b	b	b	b	b	—	—	—	—
Characteristic fragment ion c	c−2H	c−2H	c+O	c+2O	c	c	c	c−R1	—	—	—
Parent ion d	d−2H	d−2H	d+O	d+2O	d−H2−C	d−H4−C2	d−H−N+O	d−R1	d+O	d+2O	d+2H+2O

**Table 6:** Administration dose of synthetic cannabinoid of model species in vivo.

Serial Number	Abbreviation	Structural Formula	Injection Dose
1	JWH-019	C21H29NO	0.1 mg/kg
2	UR-144	C25H25NO	0.05 mg/kg
3	5F-EMB-PICA	C21H29FN2O3	0.1 mg/kg
4	AMB-FUBICA	C22H23FN2O3	0.05 mg/kg
5	FUB-144	C23H24FNO	0.5 mg/kg
6	5F-MDMB-PINACA,5F-ADB	C20H28FN3O3	0.5 mg/kg
7	AB-FUBINACA	C20H21FN4O2	0.5 mg/kg
8	ADB-4en-PINACA	C19H26N4O2	0.1 mg/kg
9	EDMB-PINACA	C21H31N3O3	0.05 mg/kg
10	ADB-CHMINACA	C21H30N4O2	0.5 mg/kg
11	BIM-018	C23H22N2O	0.05 mg/kg
12	JWH-030	C20H21NO	0.5 mg/kg
13	FUBIMINA,BIM-2201	C23H21FN2O	0.1 mg/kg
14	EMB-FUBINACA	C22H24FN3O3	0.5 mg/kg
15	ADB-P7AICA	C19H28N4O2	0.5 mg/kg
16	5F-EMB-PINACA	C20H28FN3O3	0.1 mg/kg
17	AB-4en-PINACA	C18H24N4O2	0.5 mg/kg
18	ADB-3en-BUTINACA	C18H24N4O2	0.5 mg/kg
19	ADB-HEXINACA	C20H30N4O2	0.1 mg/kg
20	5F-CYPPICA	C18H23FN2O	0.05 mg/kg
21	5F-MDMB-PICA	C21H29FN2O3	0.05 mg/kg
22	MDMB-4en-PINACA	C20H27N3O3	0.05 mg/kg
23	JWH-307	C26H24FNO	0.05 mg/kg
24	JWH-370	C27H27N0	0.1 mg/kg
25	AB-005	C23H23N20	0.5 mg/kg
26	JWH-200	C25H24N2O2	0.1 mg/kg

## Data Availability

Data are contained within the article and Supplementary Materials.

## Supplementary Materials

The following supporting information can be downloaded at: https://www.mdpi.com/article/10.3390/molecules30132682/s1, Figure S1: The product ion spectra and fragmentation of various SCs; Figure S2: Metabolic reaction diagrams of 26 SCs; Table S1: 26 SCs parent compounds and metabolite information.

## Abbreviations

The following abbreviations are used in this manuscript:

ACN	acetonitrile
NPS	new psychoactive substance
SCs	synthetic cannabinoids
UHPLC-QE Orbitrap MS	ultra-high performance liquid chromatography-Q Exactive Orbitrap mass spectrometry
HLMs	human liver microsomes
CB1R	cannabinoid receptor type 1
CB2R	cannabinoid receptor type 2
