# Supplementary material for: Systematic Characterization of In Vitro and In Vivo Metabolic Pathways and Identification of Novel Biomarkers of 26 Synthetic Cannabinoids
Source: Molecules. 2025 Jun 21;30(13):2682. doi: 10.3390/molecules30132682 (PMC12250893; doi:10.3390/molecules30132682)
Supplement: Supplementary file 1 [file molecules-30-02682-s001.zip › Supplementary Materials-Figure S2.pdf]

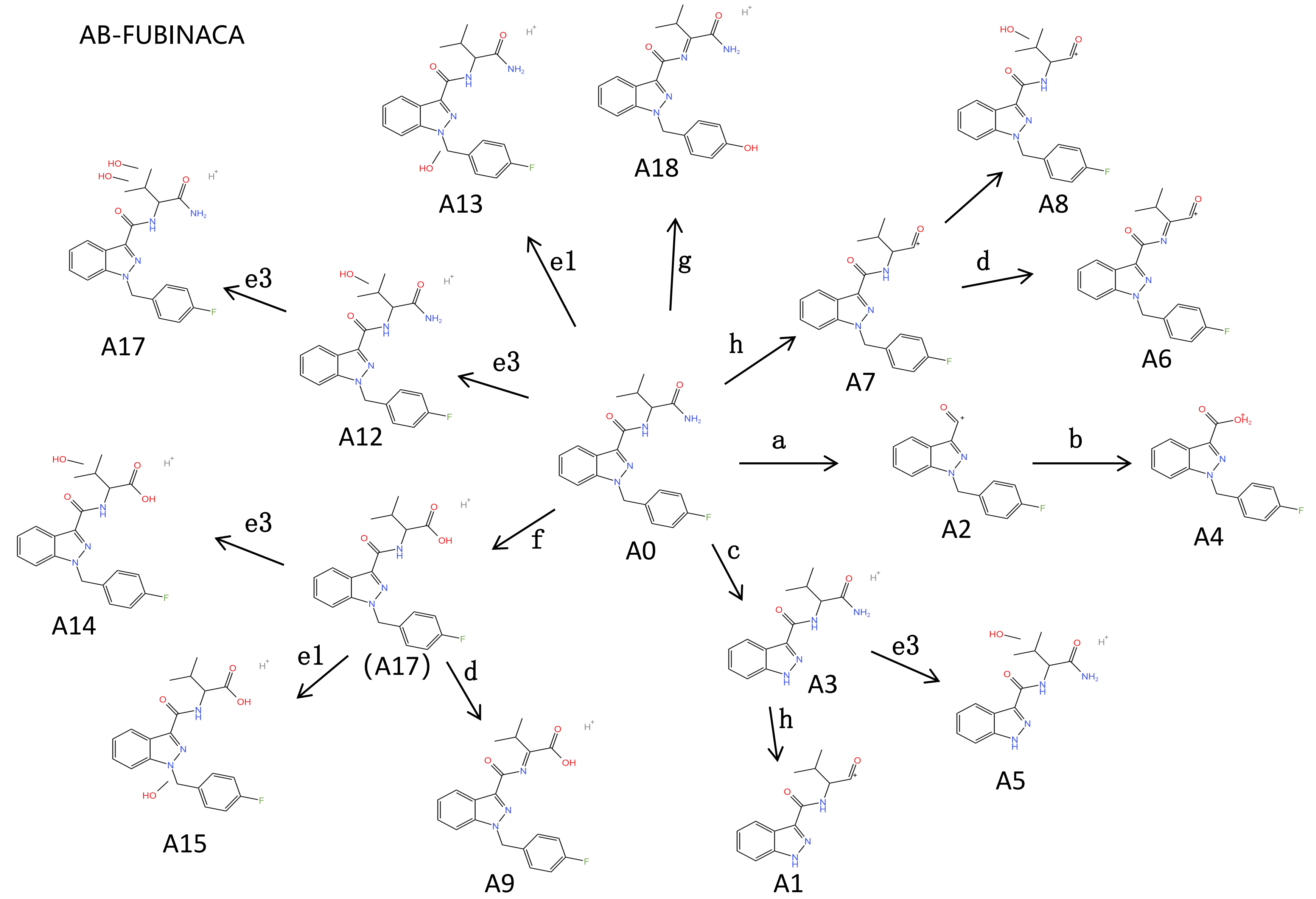

a. deamidation, b. amide hydrolysis, c. dehydrated N-phenyl side chains, d. dehydrogenation, e. hydroxylation (e1. N-Phenyl side chain, e2. indole ring, e3. isopropyl) , f. hydrolysis, g.oxidative defluoridation, h.Deamination

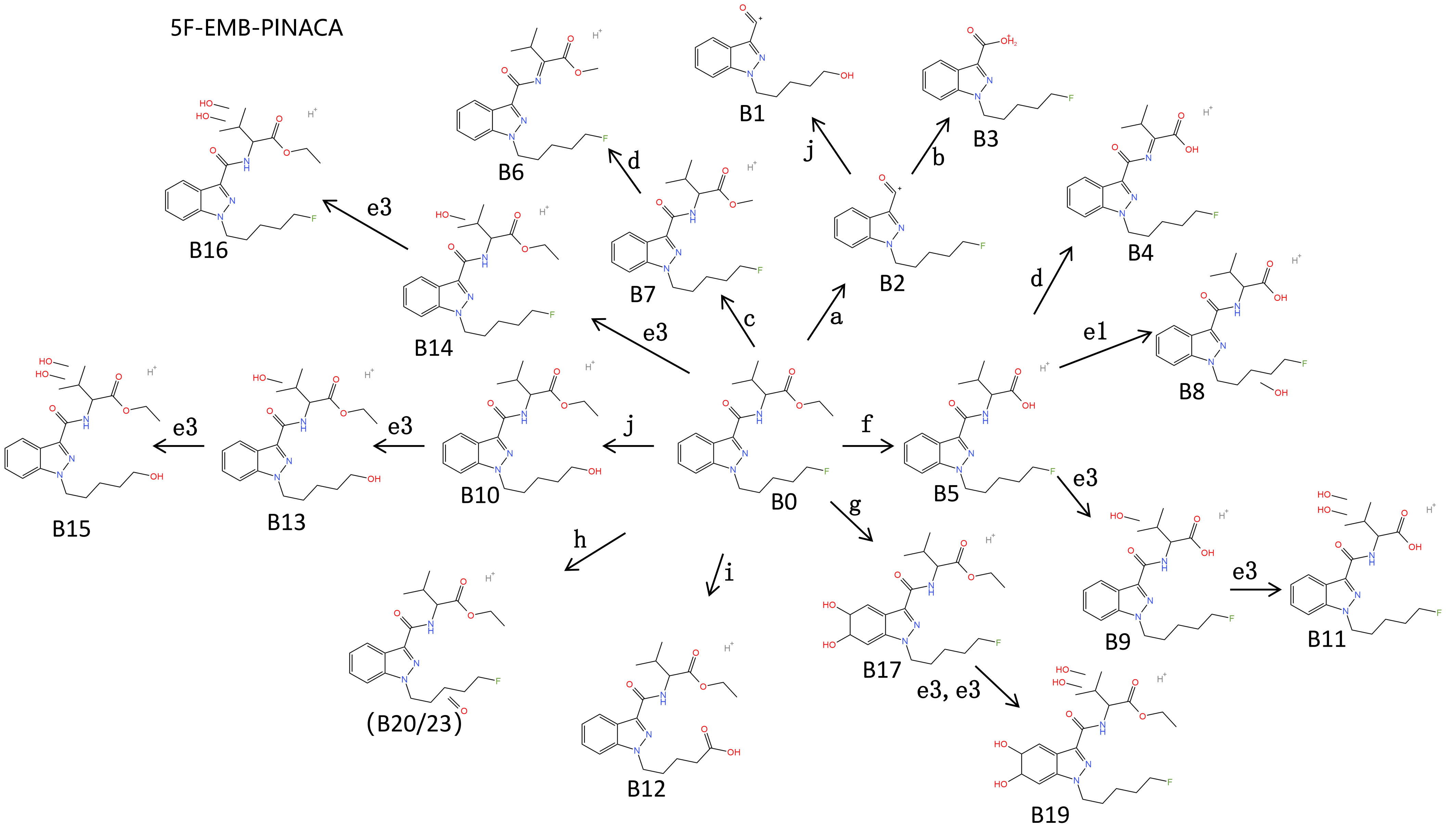

a. deamidation, b. amide hydrolysis, c. demethylation, d. dehydrogenation, e. hydroxylation (e1. N-alkyl side chains, e2. indole ring, e3. isopropyl) , f. ester hydrolysis, g.dihydrodiol, h.ketone formation, i.acidification, j.oxidative defluoridation

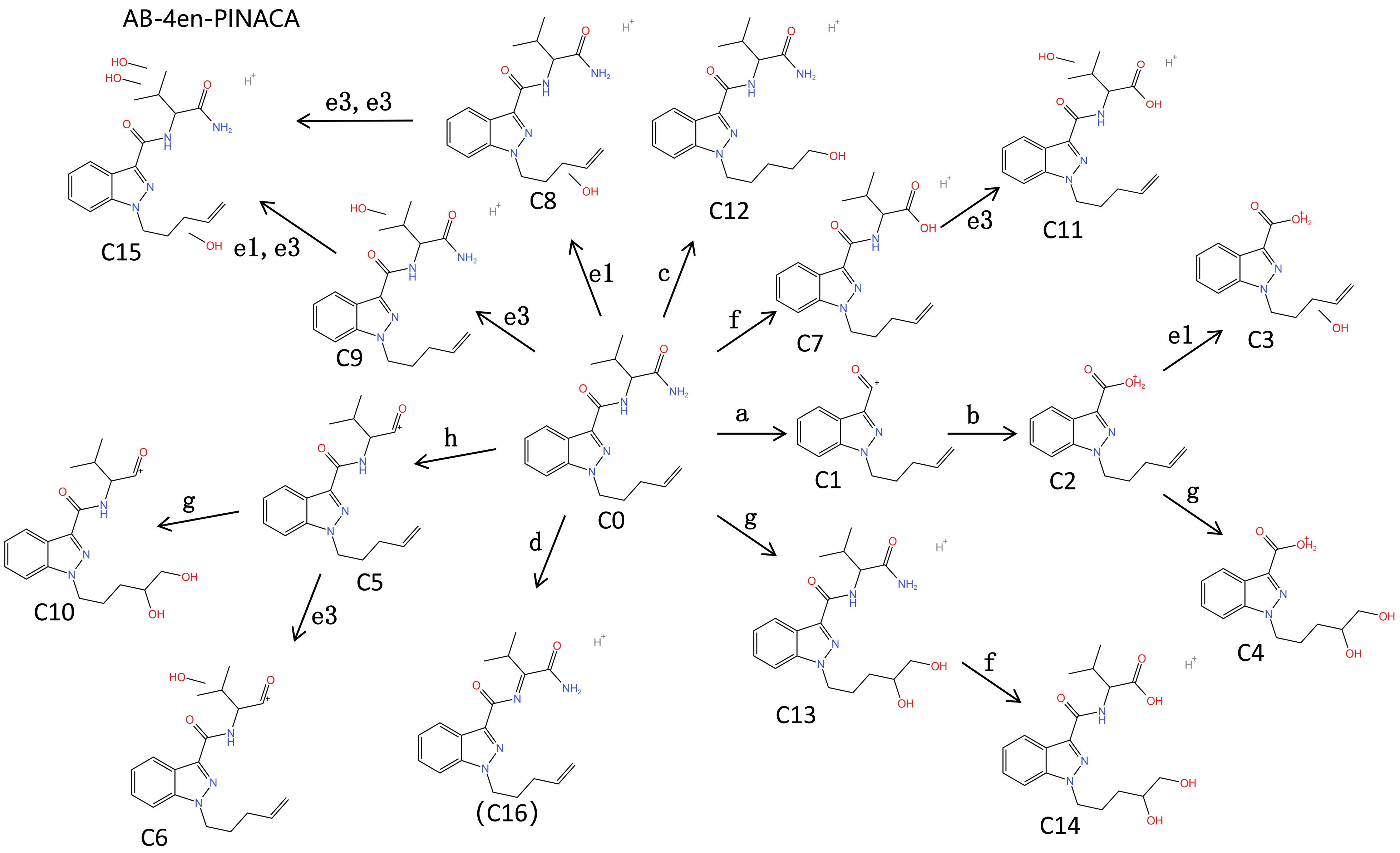

a. deamidation，b. amide hydrolysis，c. hydration，d. dehydrogenation，e. hydroxylation（e1. N-alkyl side chains，e2. indole ring，e3. isopropyl），f. hydrolysis，g.dihydrodiol，h.deester group removal

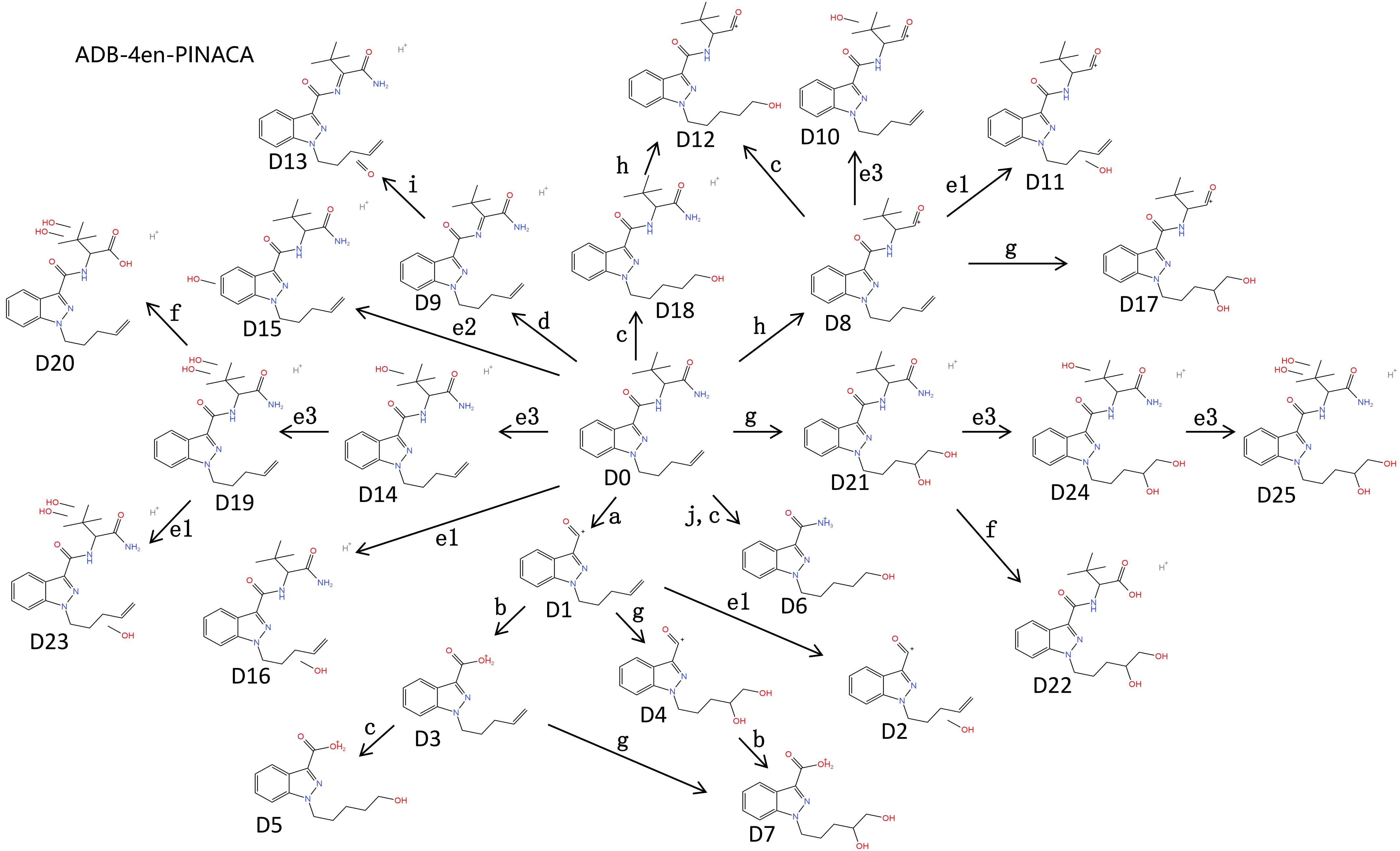

a. deamidation, b. amide hydrolysis, c. hydration, d. dehydrogenation, e. hydroxylation (e1. N-alkyl side chains, e2. Indazole ring, e3. tert-butyl) , f. hydrolysis, g.dihydrodiol, h.deester group removal, i.ketone formation, j.deamidation (C-N bond at amide junction is broken)

ADB-CHMINACA

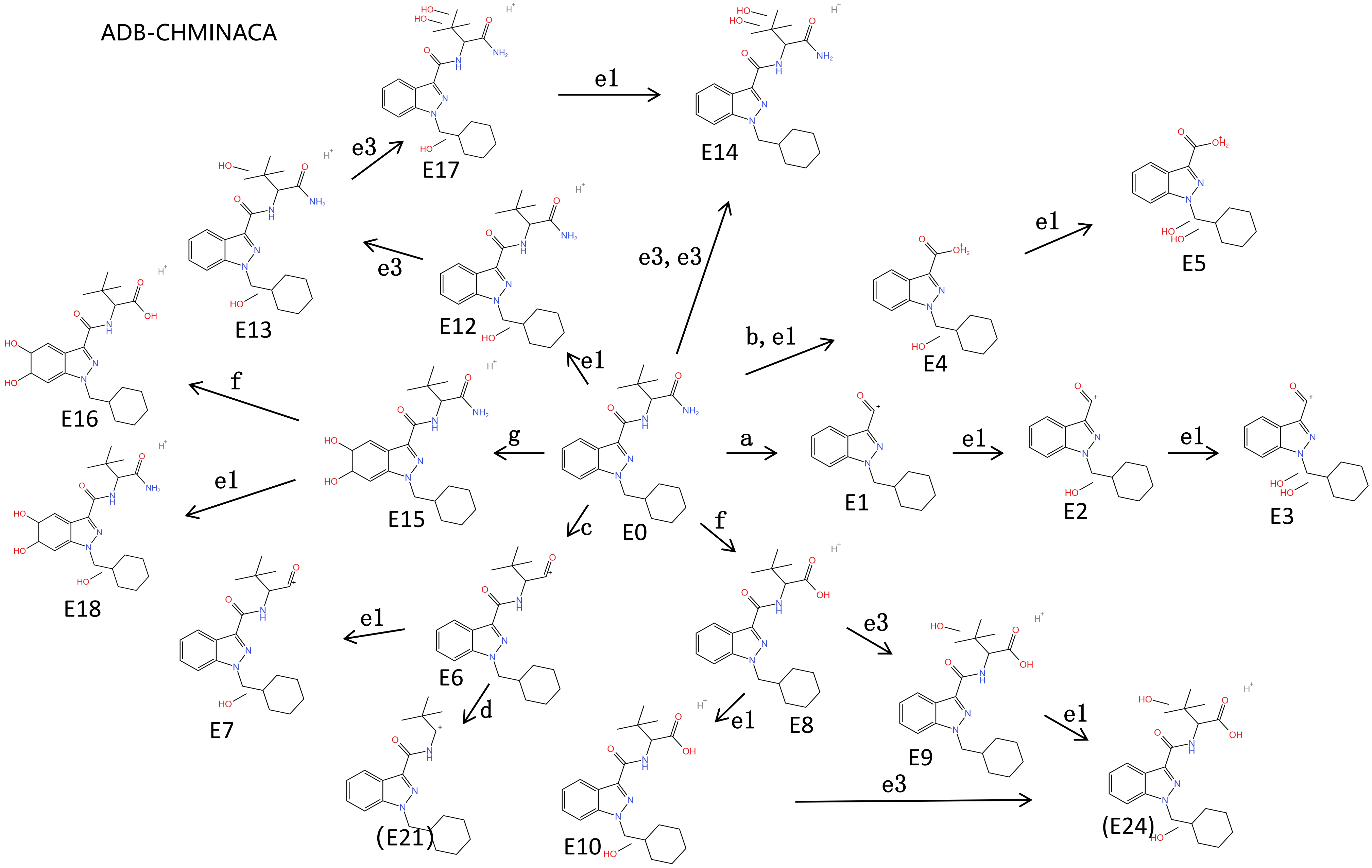

a. deamidation, b. amide hydrolysis, c.deester group removal, d. decarbonyl, e. hydroxylation (e1. N-alkyl side chains, e2. Indazole ring, e3. isopropyl) , f. hydrolysis, g.dihydrodiol

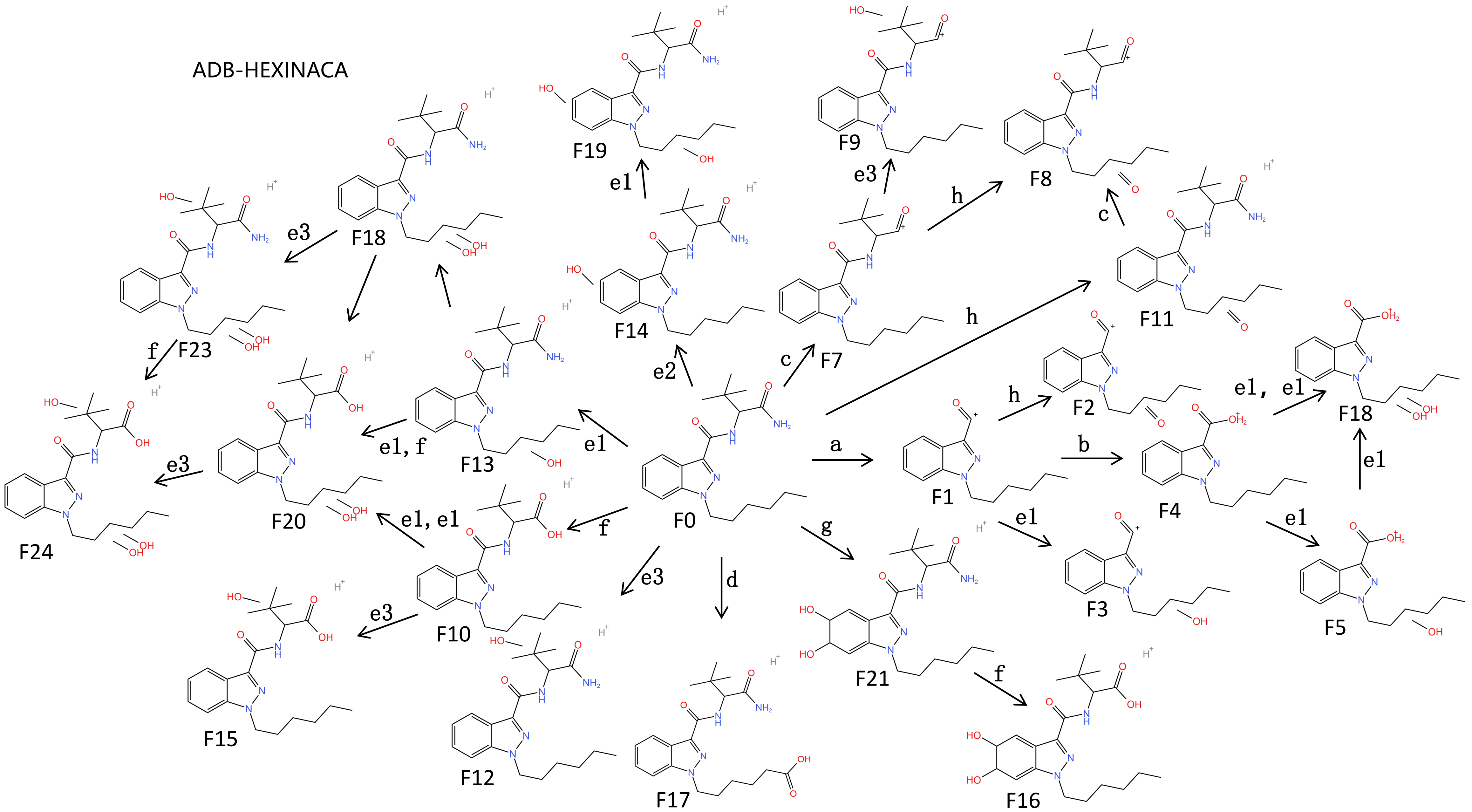

a. deamidation, b. amide hydrolysis, c. deester group removal, d. acidification, e. hydroxylation (e1. N-alkyl side chains, e2. Indazole ring, e3. tert-butyl) , f. ester hydrolysis, g.dihydrodiol, h.ketone formation

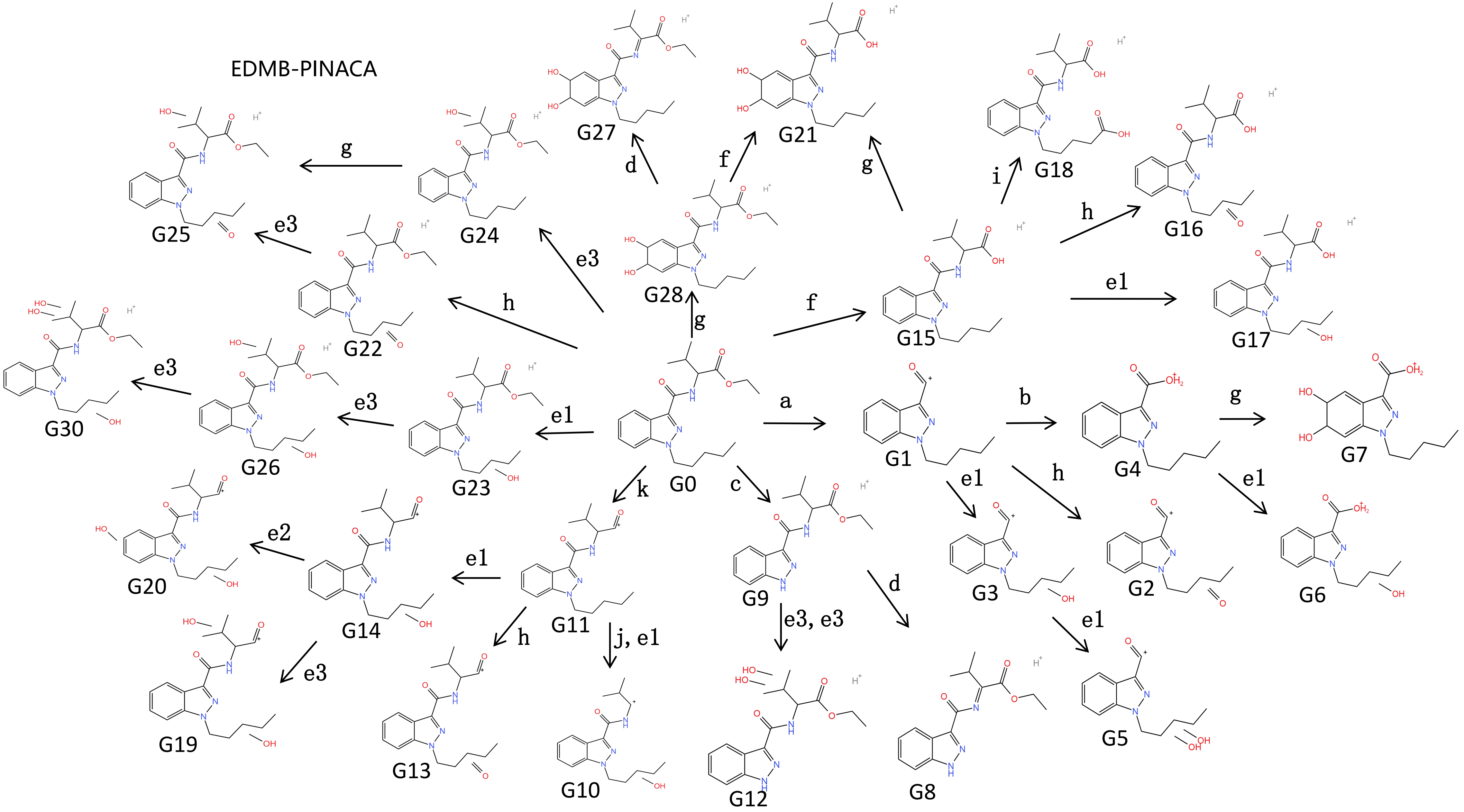

a. deamidation, b. amide hydrolysis, c. N- alkyl side chain removal, d. dehydrogenation, e. hydroxylation (e1. N-alkyl side chains, e2. Indazole ring, e3. isopropyl) , f. ester hydrolysis, g.dihydrodiol, h.ketone formation, i.acidification, j.decarbonyl, k.deester group removal

EMB-FUBINACA

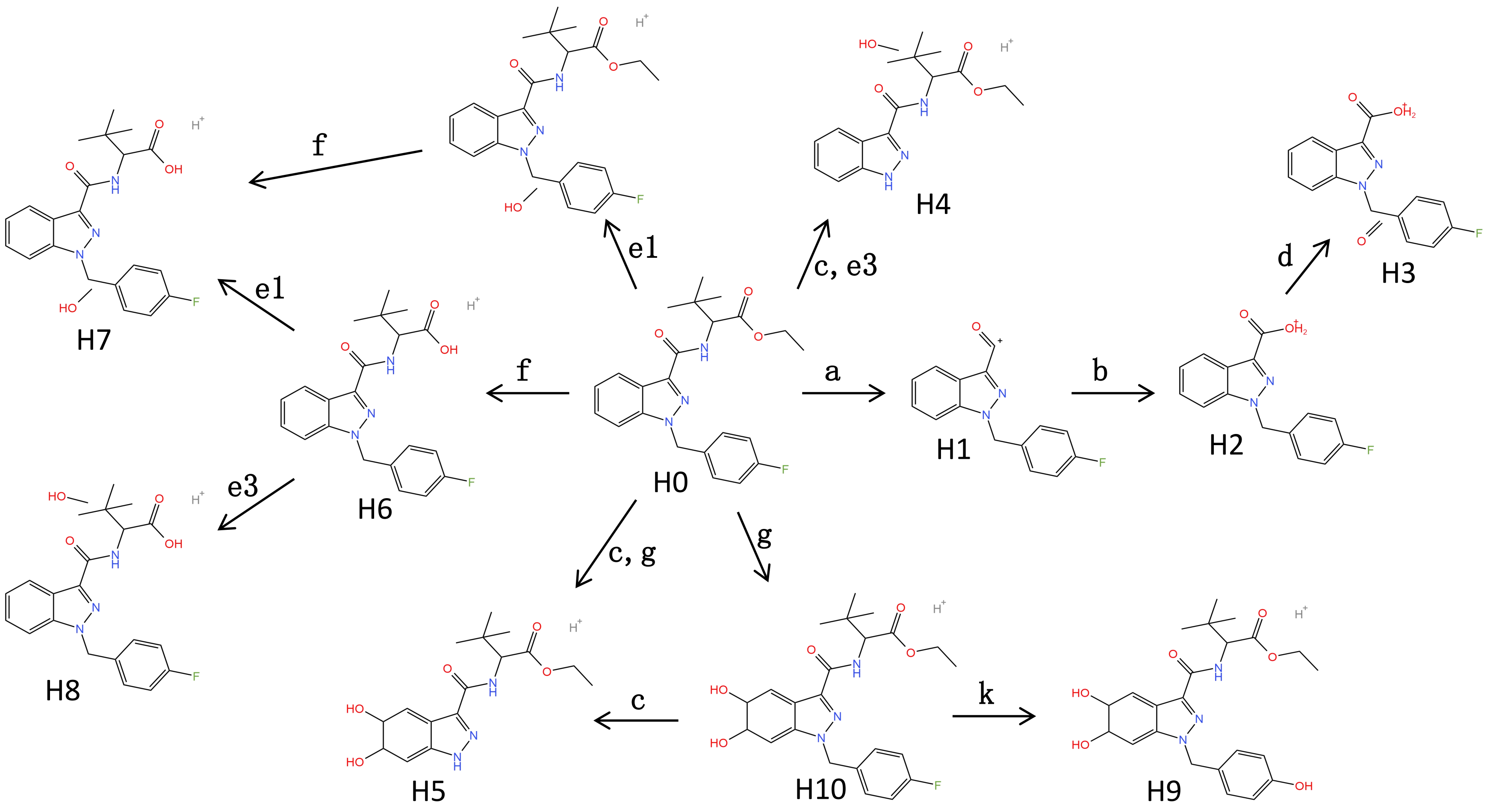

a. deamidation, b. amide hydrolysis, c. N- alkyl side chain removal, d. ketone formation, e. hydroxylation (e1. N-alkyl side chains, e2.Indazole ring, e3. tert-butyl) , f.ester hydrolysis , g.dihydrodiol, k. oxidative defluoridation

ADB-3en-BUTINACA

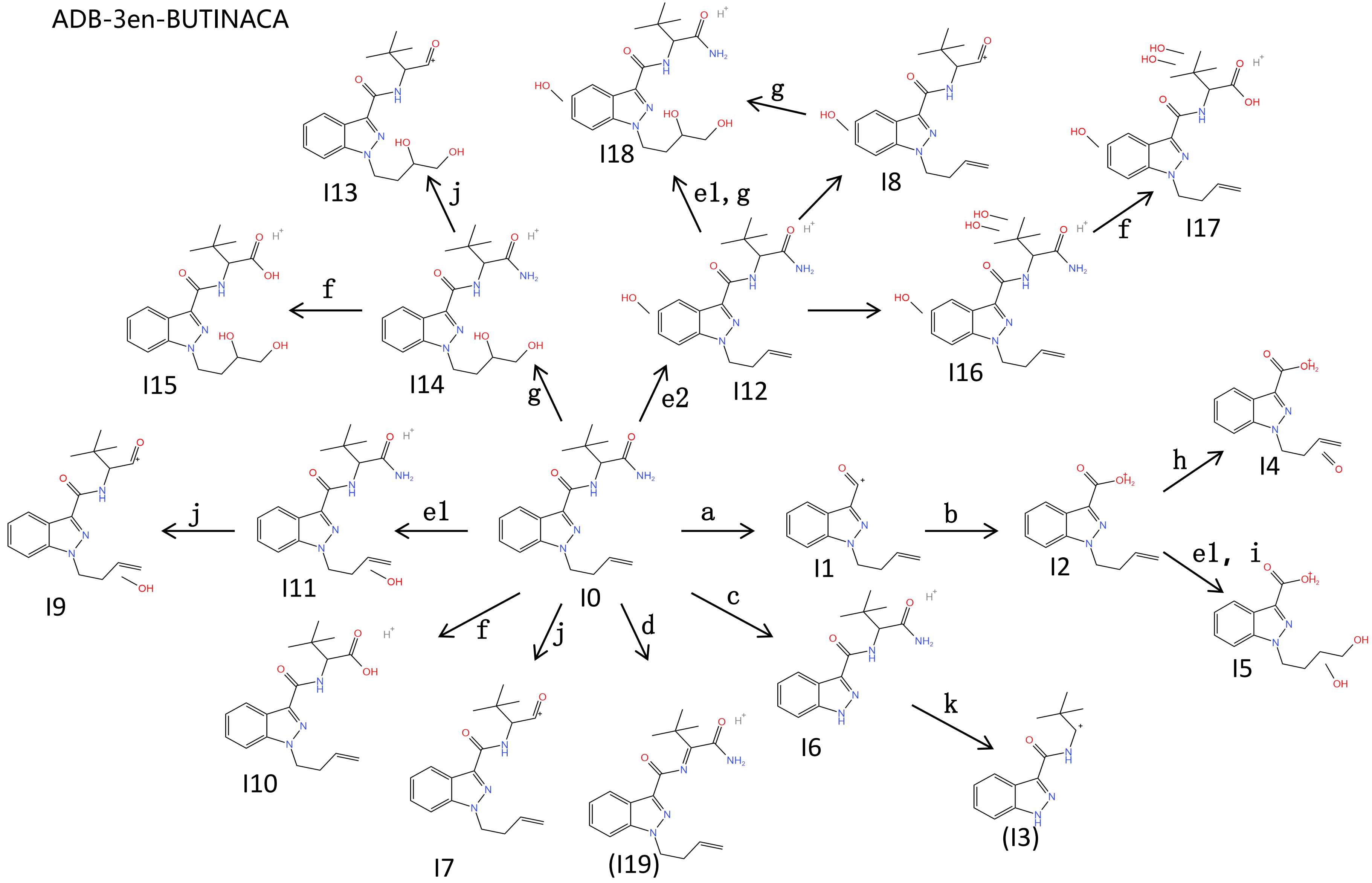

a. deamidation，b. amide hydrolysis，c. N- alkyl side chain removal，d. dehydrogenation，e. hydroxylation（e1. N-alkyl side chains，e2. Indazole ring，e3. tert-butyl），f. hydrolysis，g.dihydrodiol，h.ketone formation，i.hydration，j.Deamination，k.decarbonyl

5F-ADB

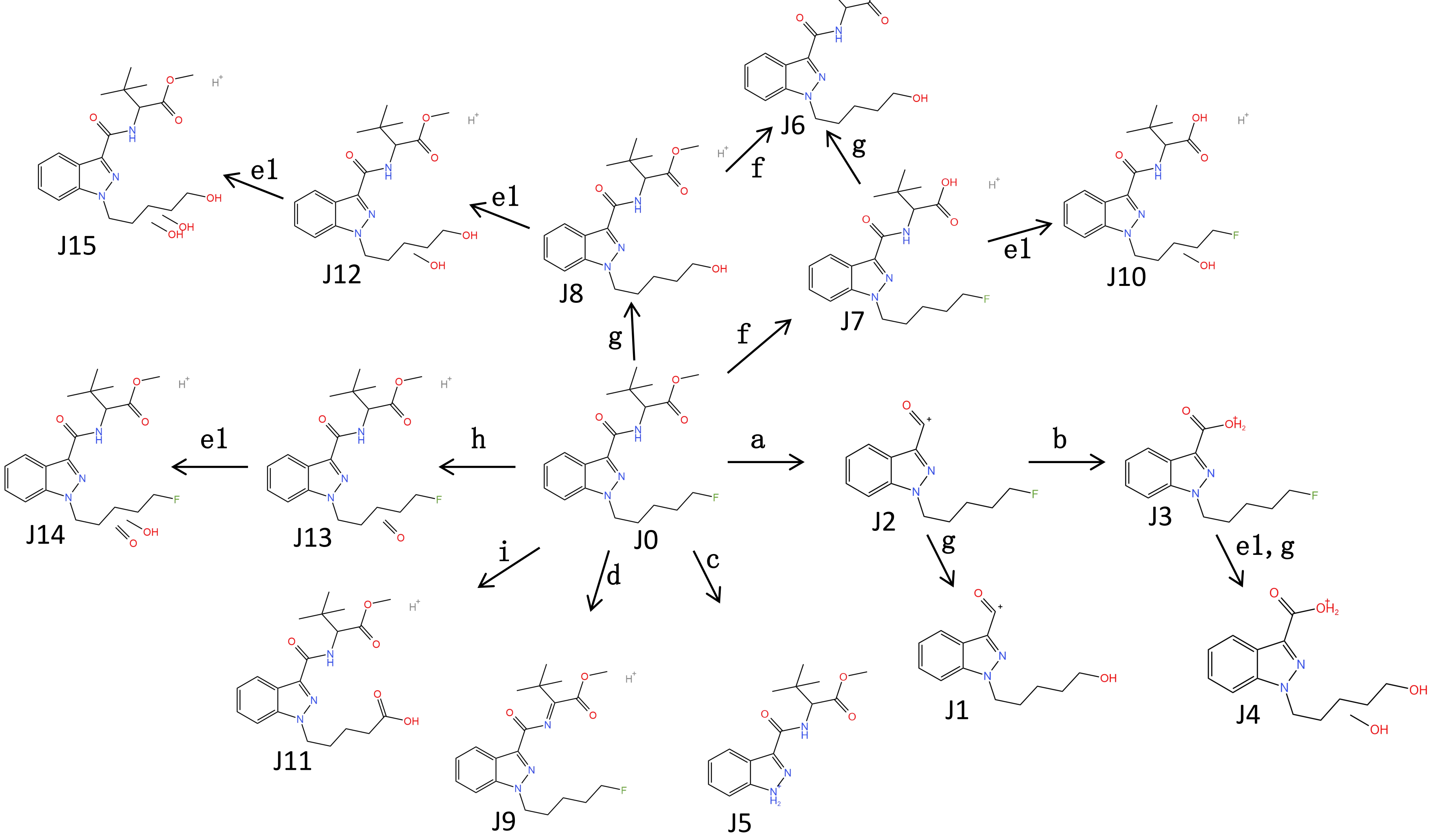

a. deamidation，b. amide hydrolysis，c. N- alkyl side chain removal，d. dehydrogenation，e. hydroxylation（e1. N-alkyl side chains，e2. Indazole ring，e3. tert-butyl），f. ester hydrolysis，g.oxidative defluoridation，h.ketone formation，i.acidification

MDMB-4en-PINACA

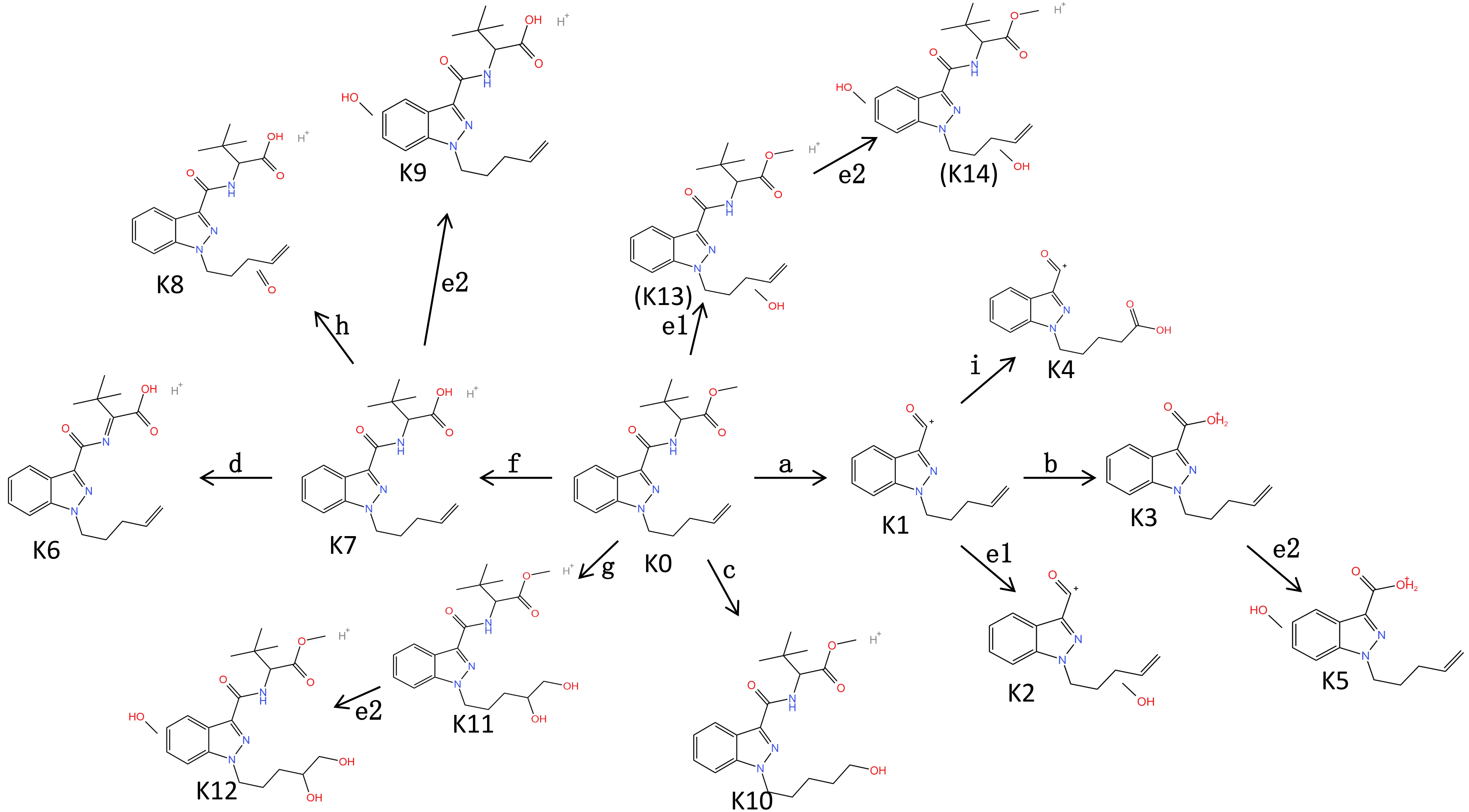

a. deamidation, b. amide hydrolysis, c. hydration, d. dehydrogenation, e. hydroxylation (e1. N-alkyl side chains, e2. Indazole ring, e3. tert-butyl), f. ester hydrolysis, g.dihydrodiol, h.ketone formation, i.acidification

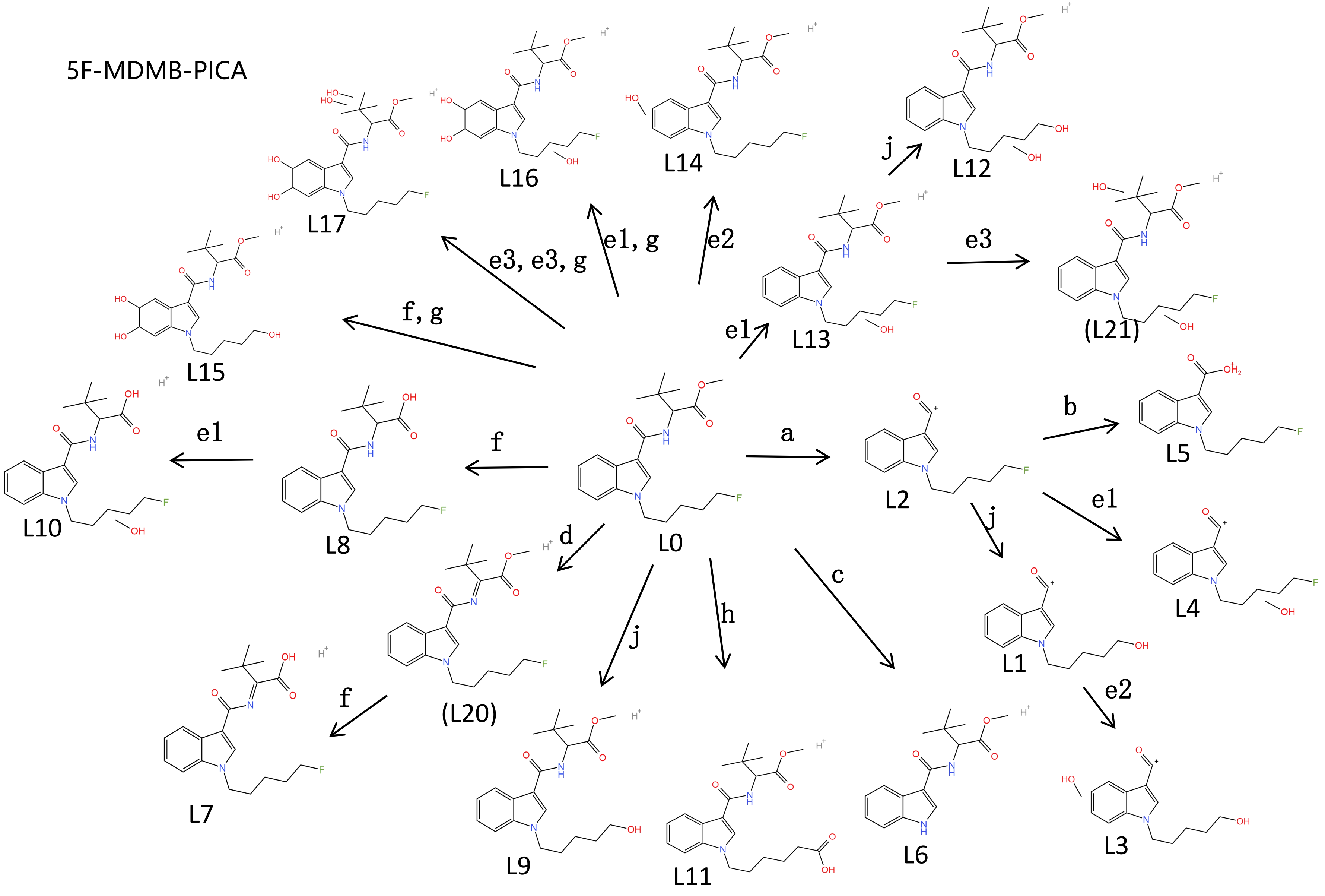

a. deamidation，b. amide hydrolysis，c. N- alkyl side chain removal，d. dehydrogenation，e. hydroxylation（e1. N-alkyl side chains，e2. indole ring，e3. tert-butyl），f. ester hydrolysis，g.dihydrodiol，h.acidification，j.oxidative defluoridation

5F-EMB-PICA

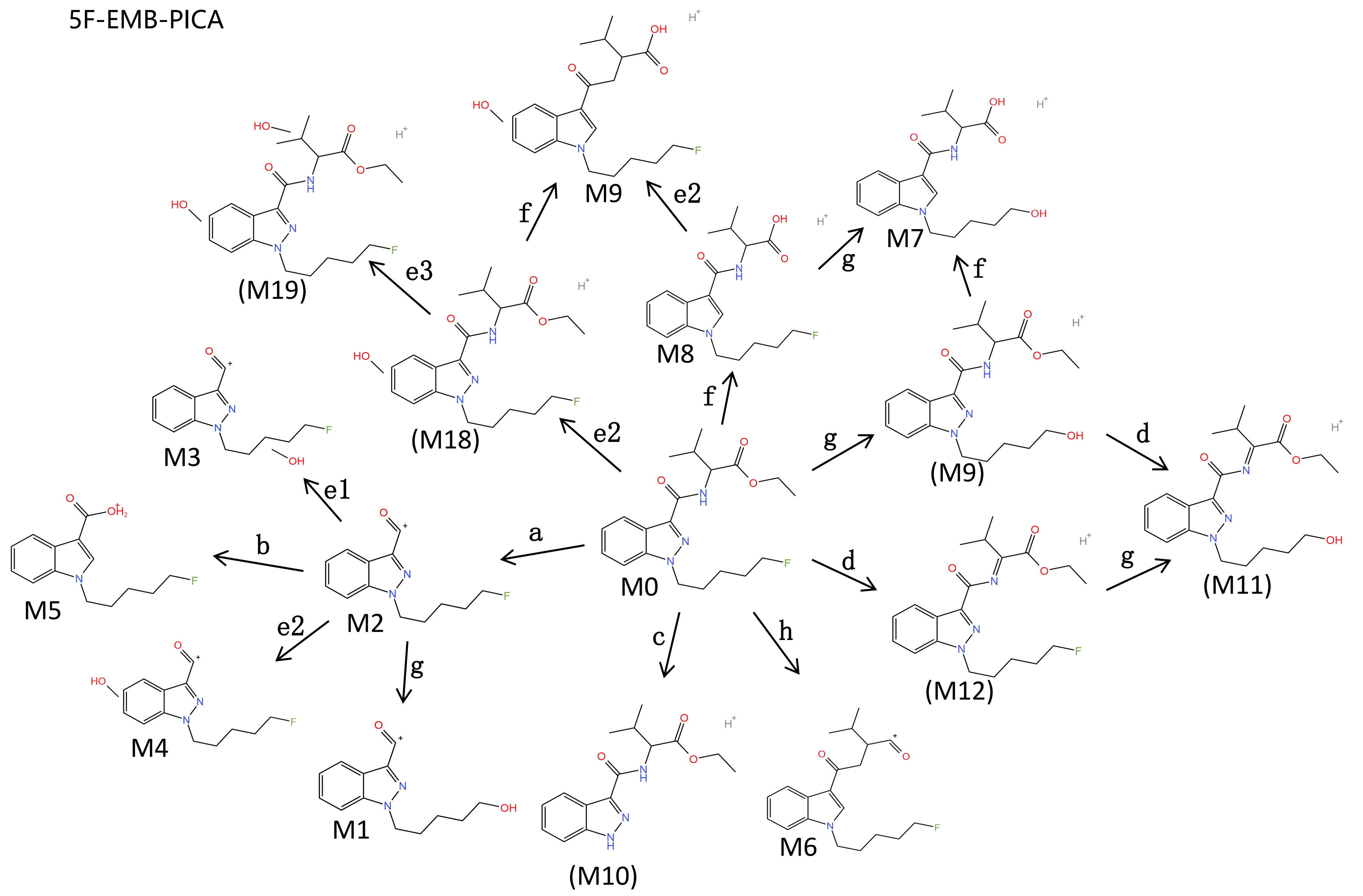

a. deamidation, b. amide hydrolysis, c. N- alkyl side chain removal, d. dehydrogenation, e. hydroxylation (e1. N-alkyl side chains, e2. indole ring, e3. isopropyl) , f. ester hydrolysis, g.oxidative defluoridation, h.deester group removal

5F-CYPPICA

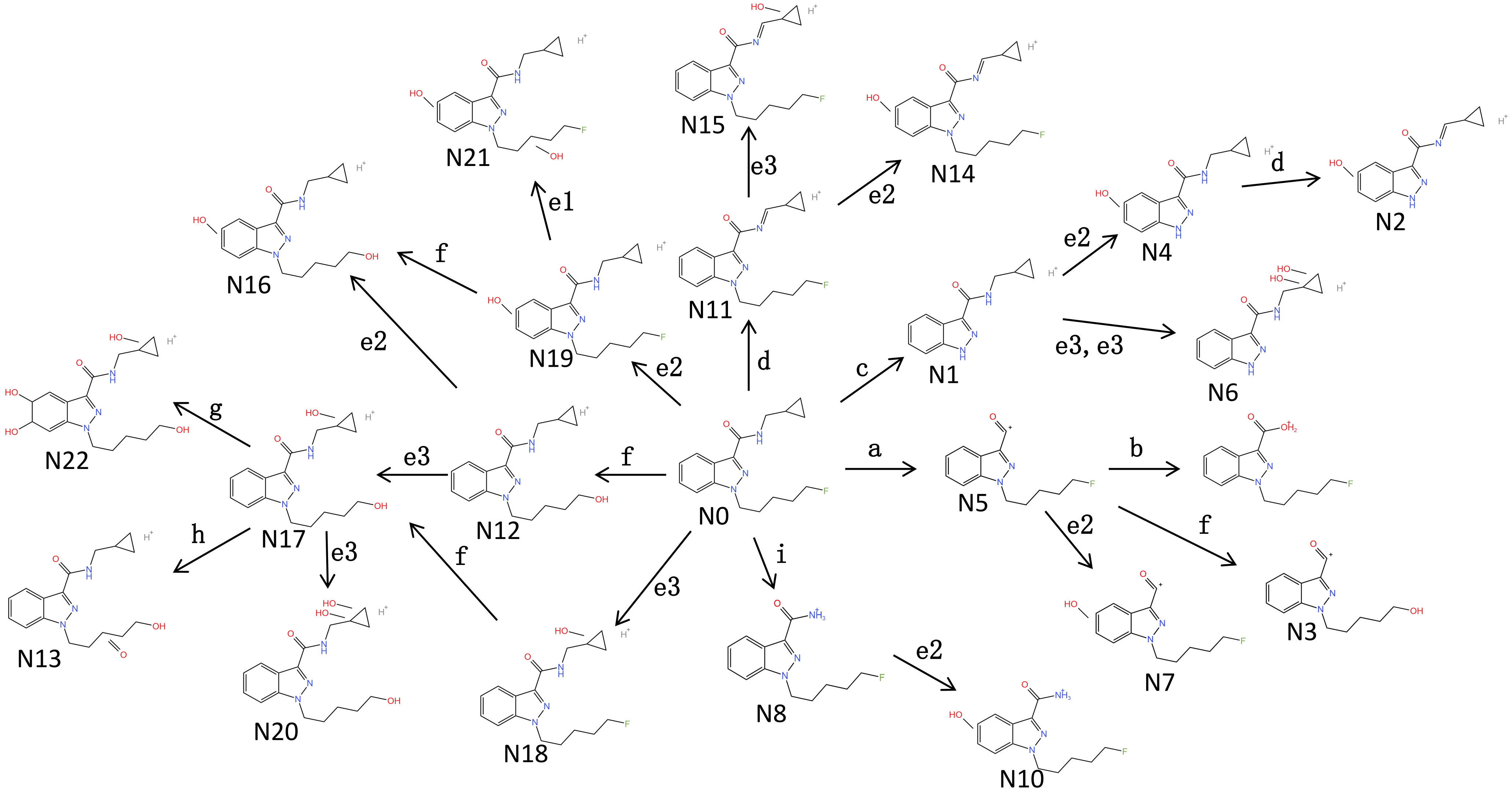

a. deamidation, b. amide hydrolysis, c. N- alkyl side chain removal, d. dehydrogenation, e. hydroxylation (e1. N-alkyl side chains, e2. indole ring, e3. tert-butyl) , f. oxidative defluoridation, g.dihydrodiol, h.ketone formation, i.decyclobutane

AMB-FUBICA

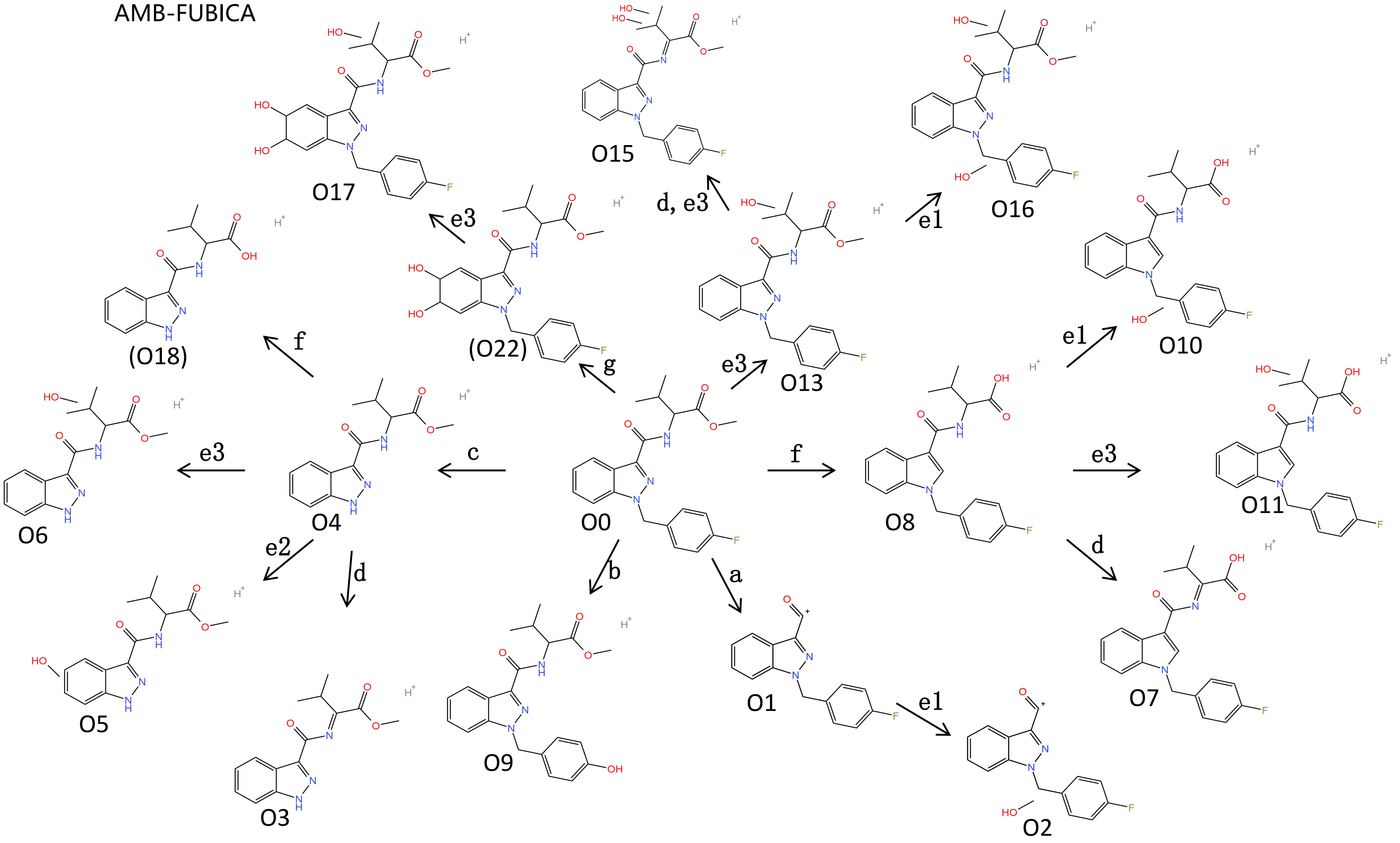

a. deamidation，b. oxidative defluoridation，c. dehydrated N-phenyl side chains，d. dehydrogenation，e. hydroxylation（e1. N-Phenyl side chain，e2. indole ring，e3. isopropyl），f. ester hydrolysis，g.dihydrodiol

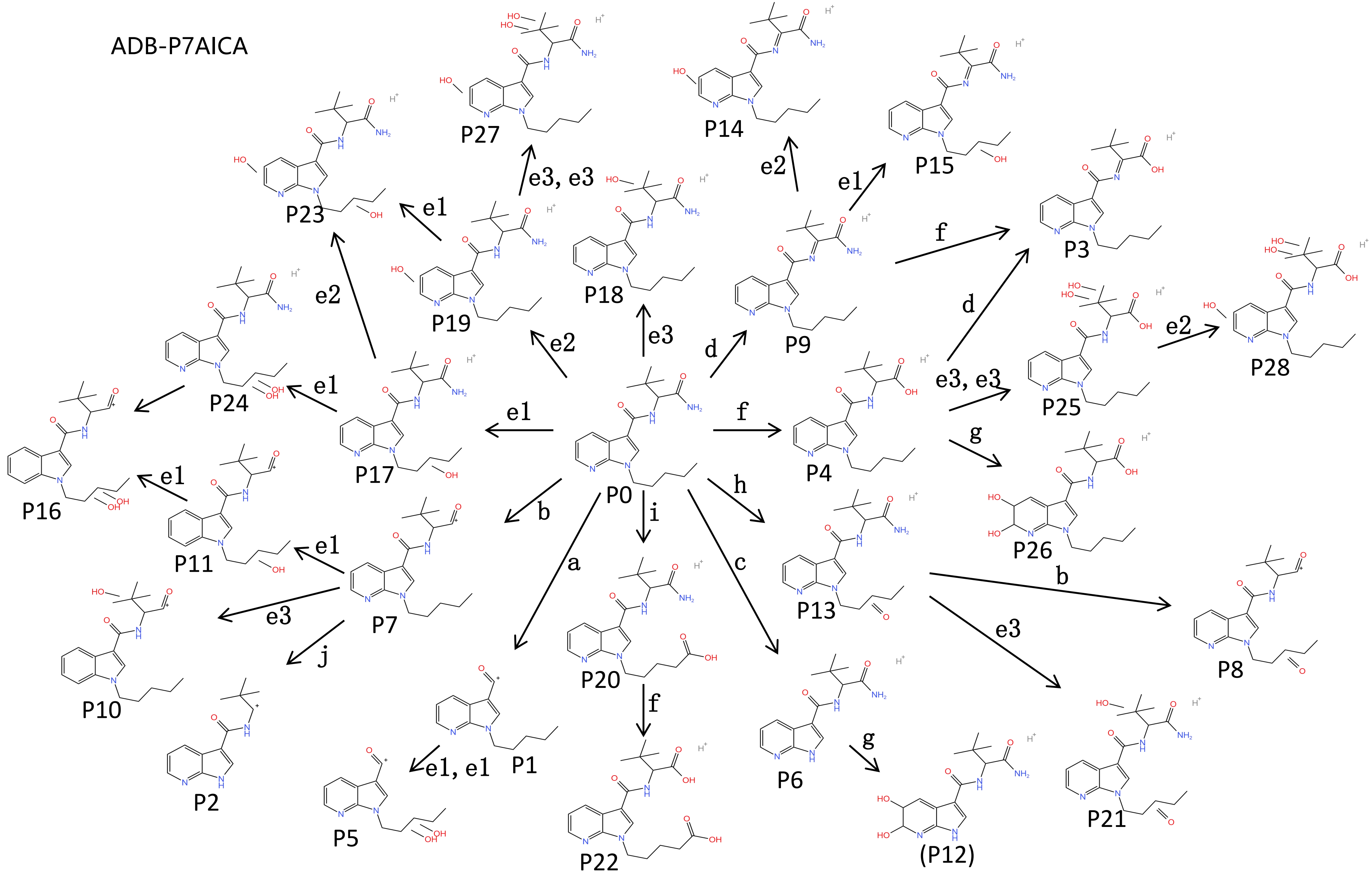

a. deamidation, b. Deamination, c. N- alkyl side chain removal, d. dehydrogenation, e. hydroxylation (e1. N-alkyl side chains, e2. indole ring, e3. tert-butyl), f. hydrolysis, g.dihydrodiol, h.ketone formation, i.acidification, j.decarbonyl

JWH-019

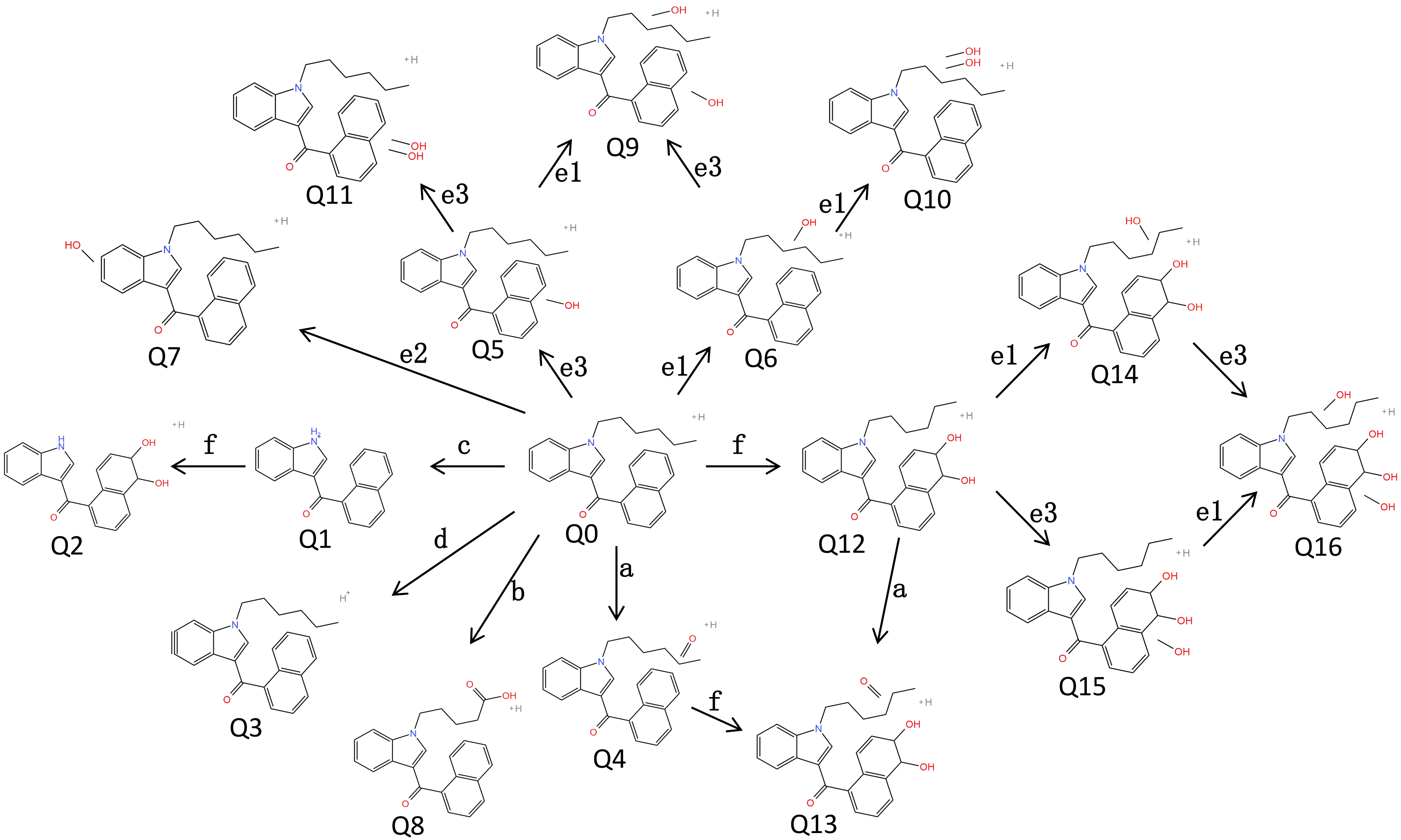

a. ketone formation, b. acidification, c. N- alkyl side chain removal, d. dehydrogenation, e. hydroxylation (e1. N-alkyl side chains, e2. indole ring, e3. naphthyl) , f. dihydrodiol

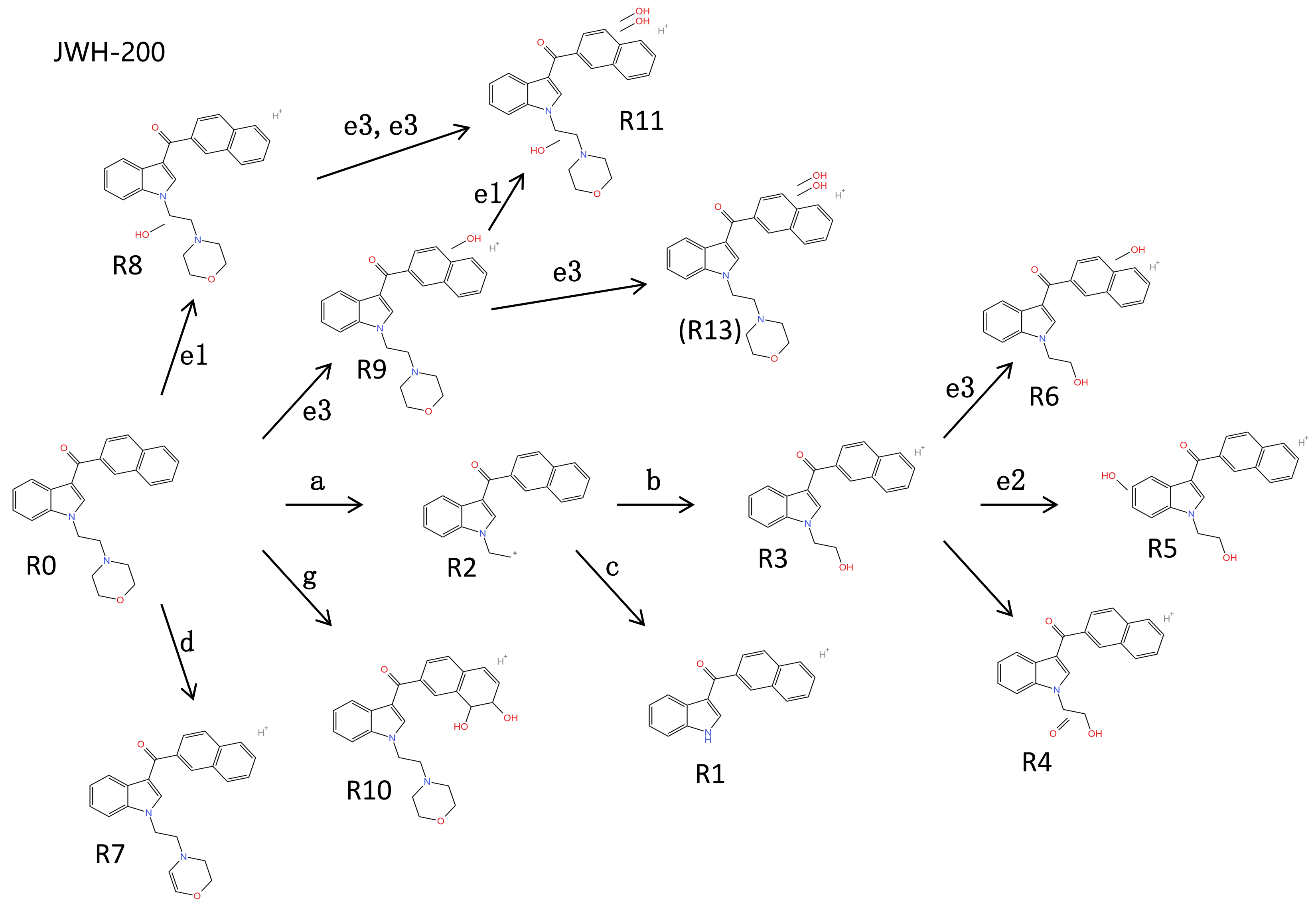

a. diethylmorpholine，b. oxidation，c. N- alkyl side chain removal，d. dehydrogenation，e. hydroxylation（e1. N-alkyl side chains，e2. indole ring，e3. naphthyl），f. dihydrodiol，g.ketone formation

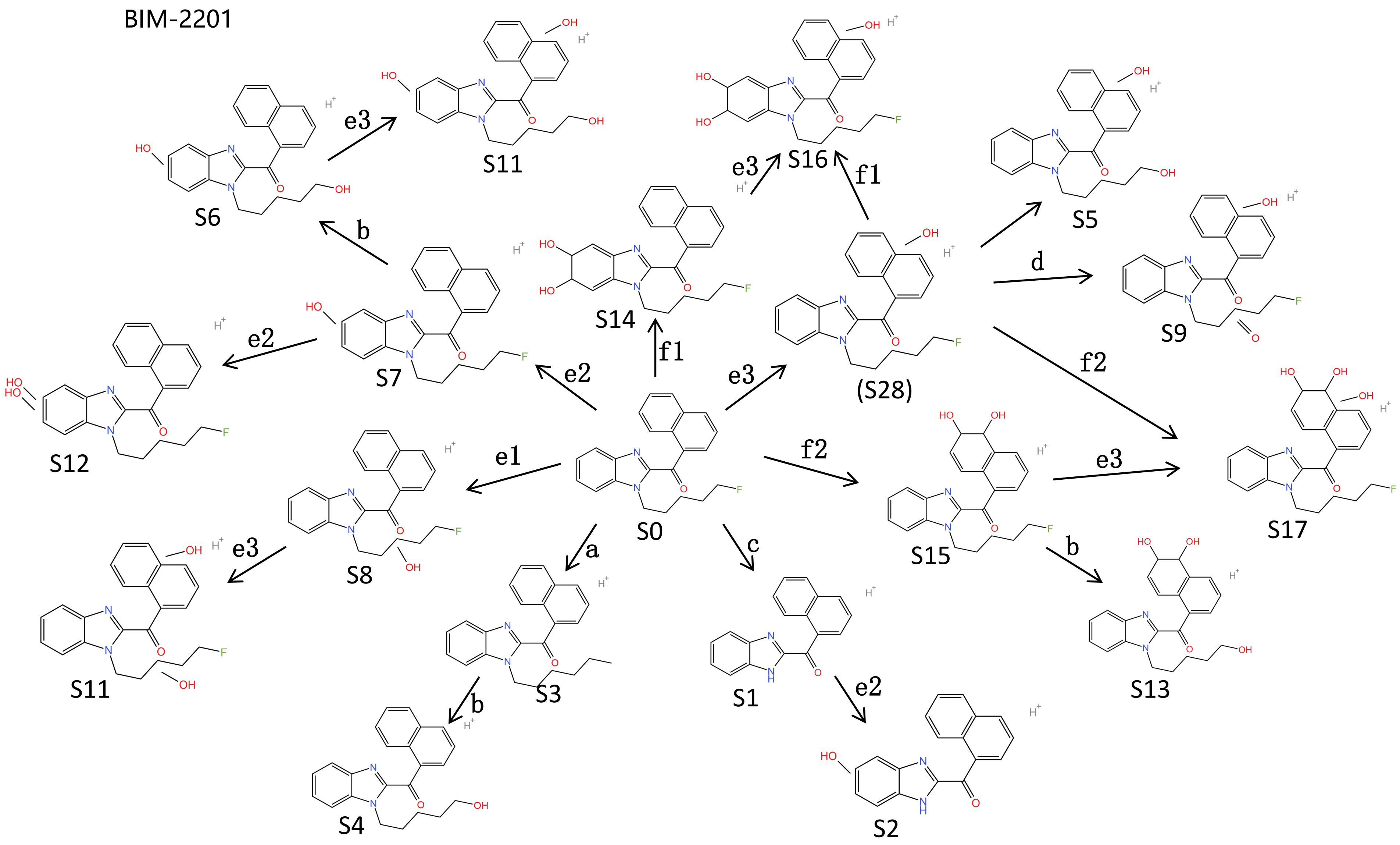

a. defluorination，b. oxidative defluoridation，c. N- alkyl side chain removal，d. ketone formation，e. hydroxylation（e1. N-alkyl side chains，e2. benzimidazole ring，e3. naphthyl），f.dihydrodiol（f1.benzimidazole ring，f2.naphthyl）

BIM-018

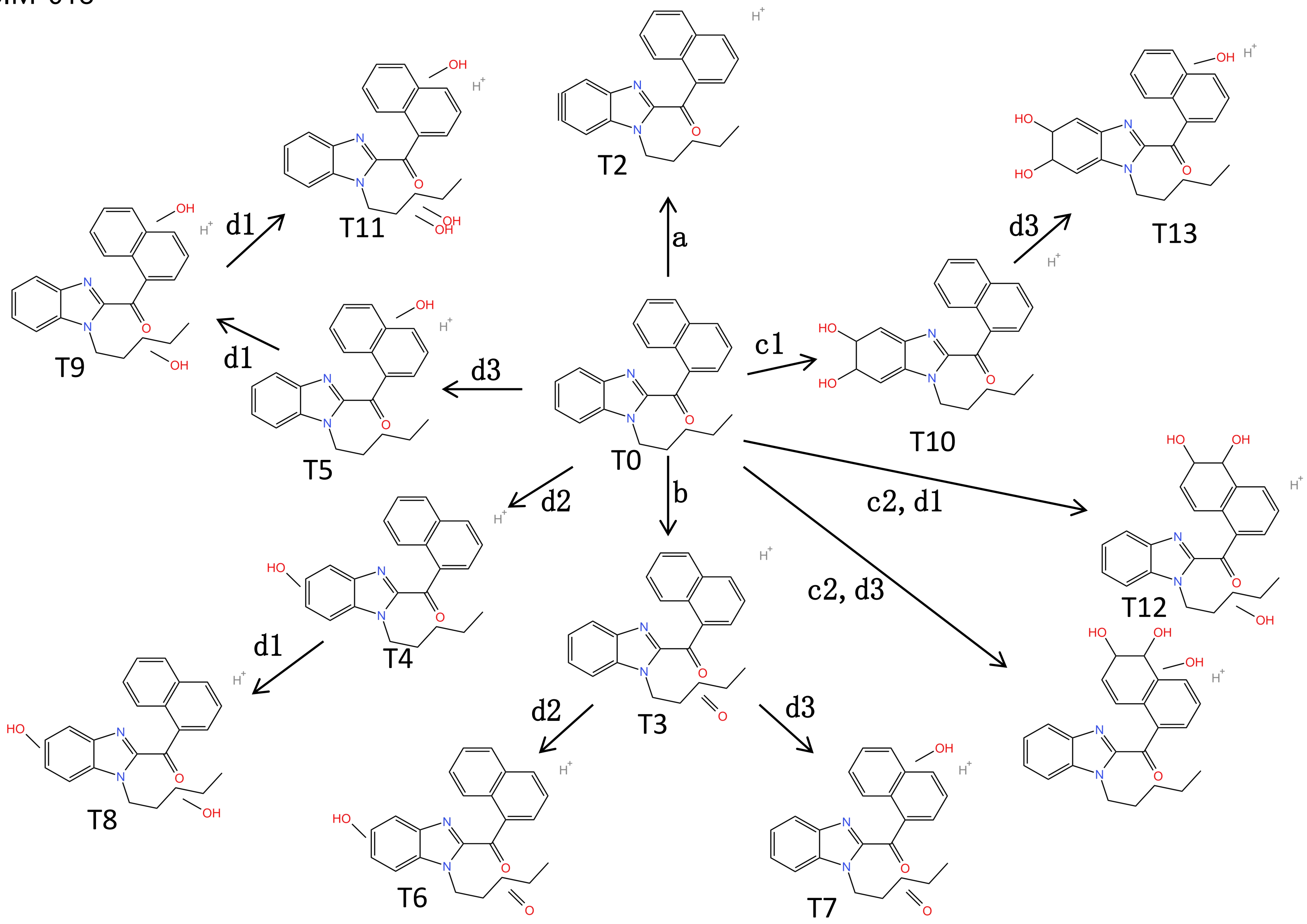

a. dehydrogenation, b. ketone formation, c. dihydrodiol (c1.benzimidazole ring, c2.naphthyl) , d. hydroxylation (d1. N-alkyl side chains, d2. benzimidazole ring, d3. naphthyl)

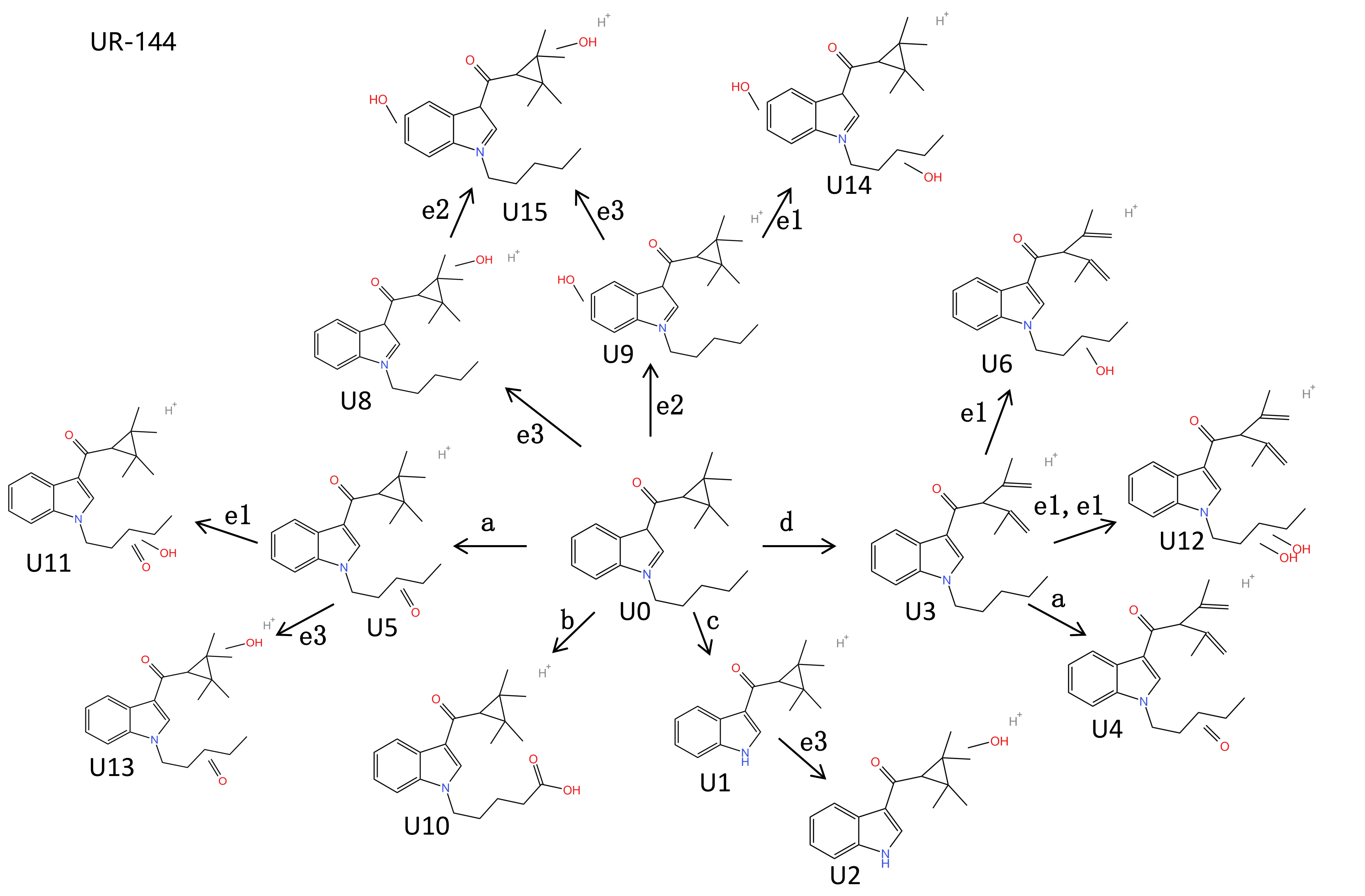

a. ketone formation, b. acidification, c. N- alkyl side chain removal, d. dehydrogenation, e. hydroxylation (e1. N-alkyl side chains, e2. indole ring, e3. cyclopropyl)

AB-005

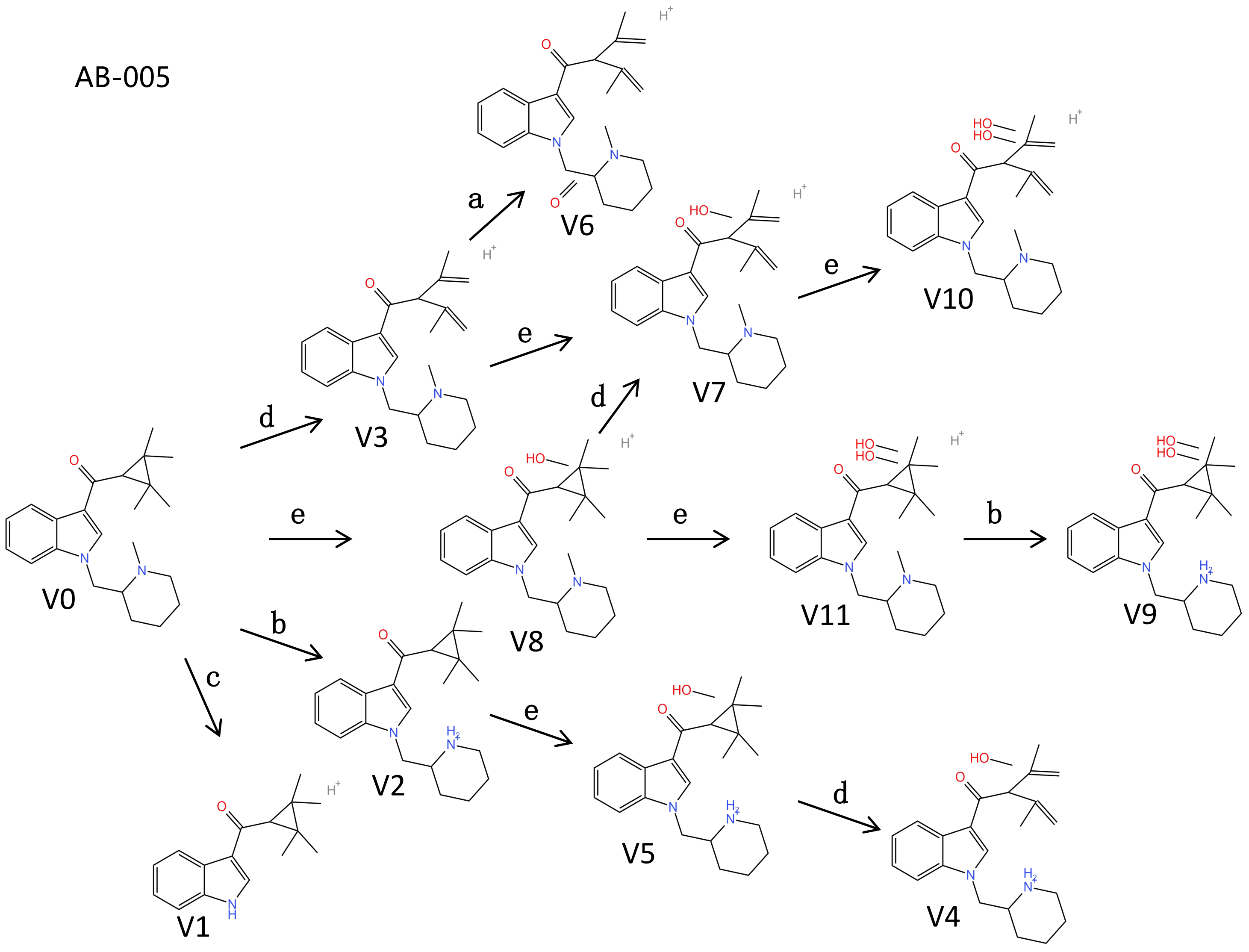

a. ketone formation, b. demethylation, c. N- alkyl side chain removal, d. dehydrogenation, e. hydroxylation (cyclopropyl)

FUB-144

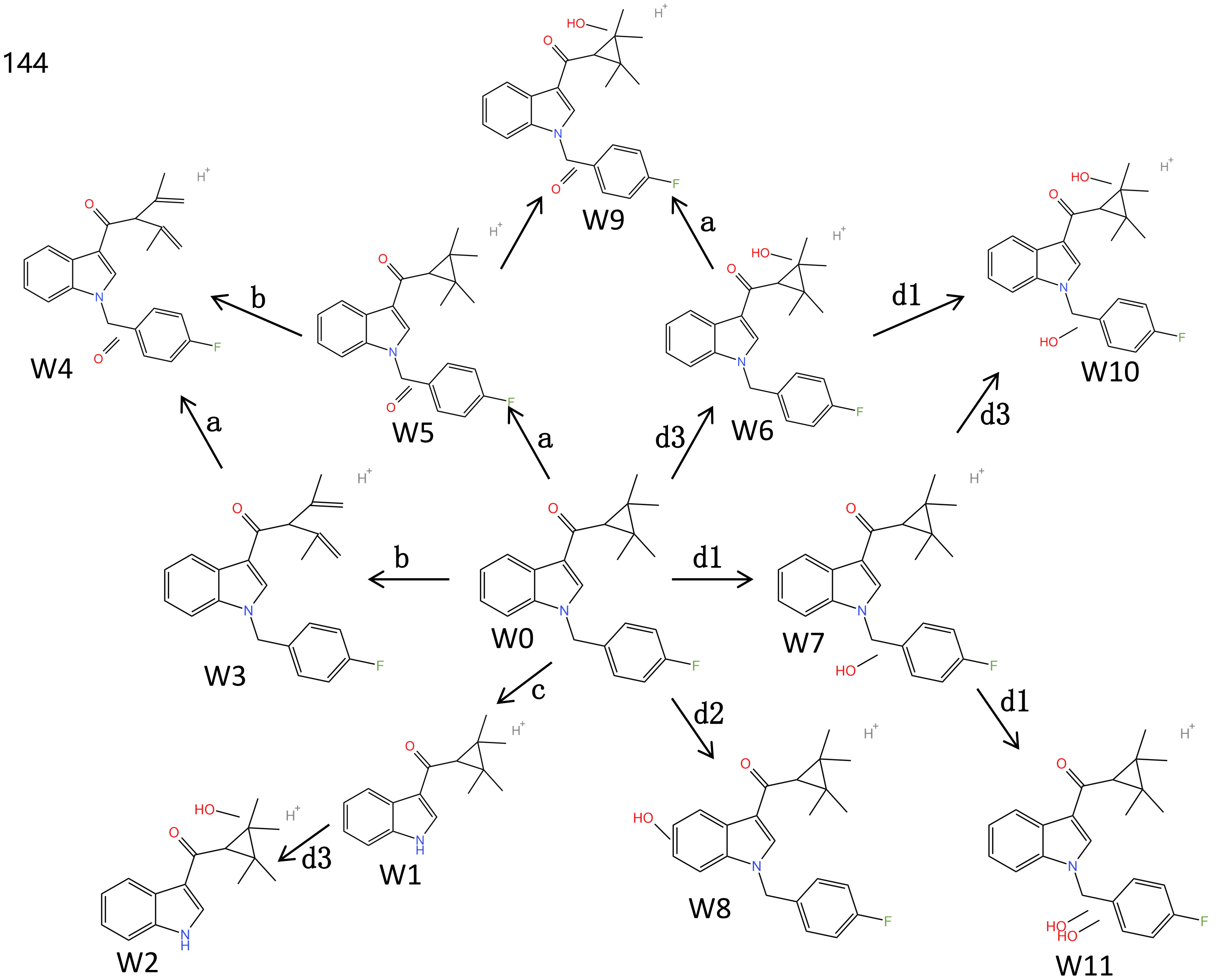

a. ketone formation, b. dehydrogenation, c. dehydrated N-phenyl side chains, d. hydroxylation (d. N-Phenyl side chain, d2. indole ring, d3. naphthyl)

JWH-030

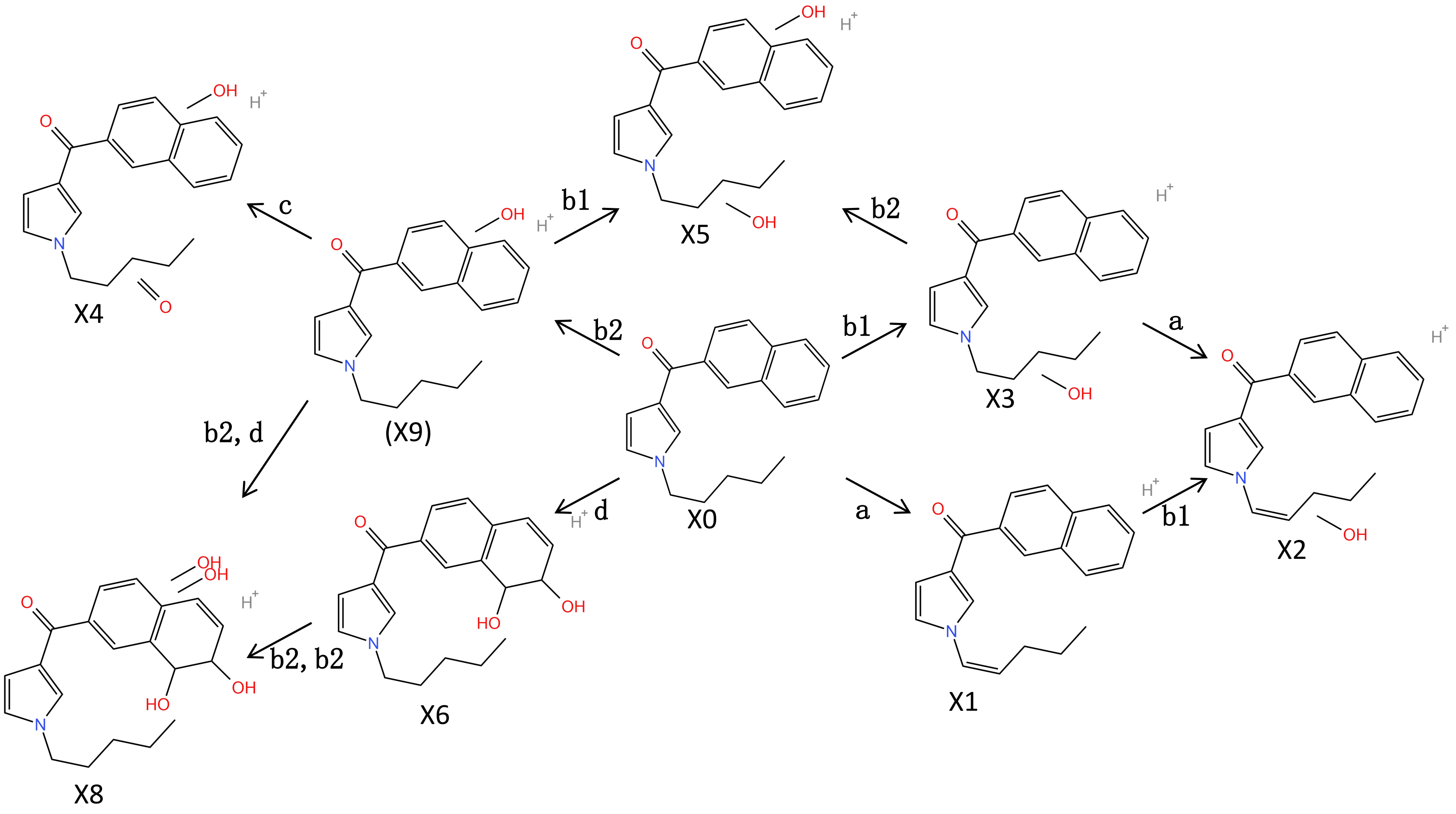

a. dehydrogenation, b. hydroxylation (b1. N-alkyl side chains, b2. naphthyl), c. ketone formation, d.dihydrodiol

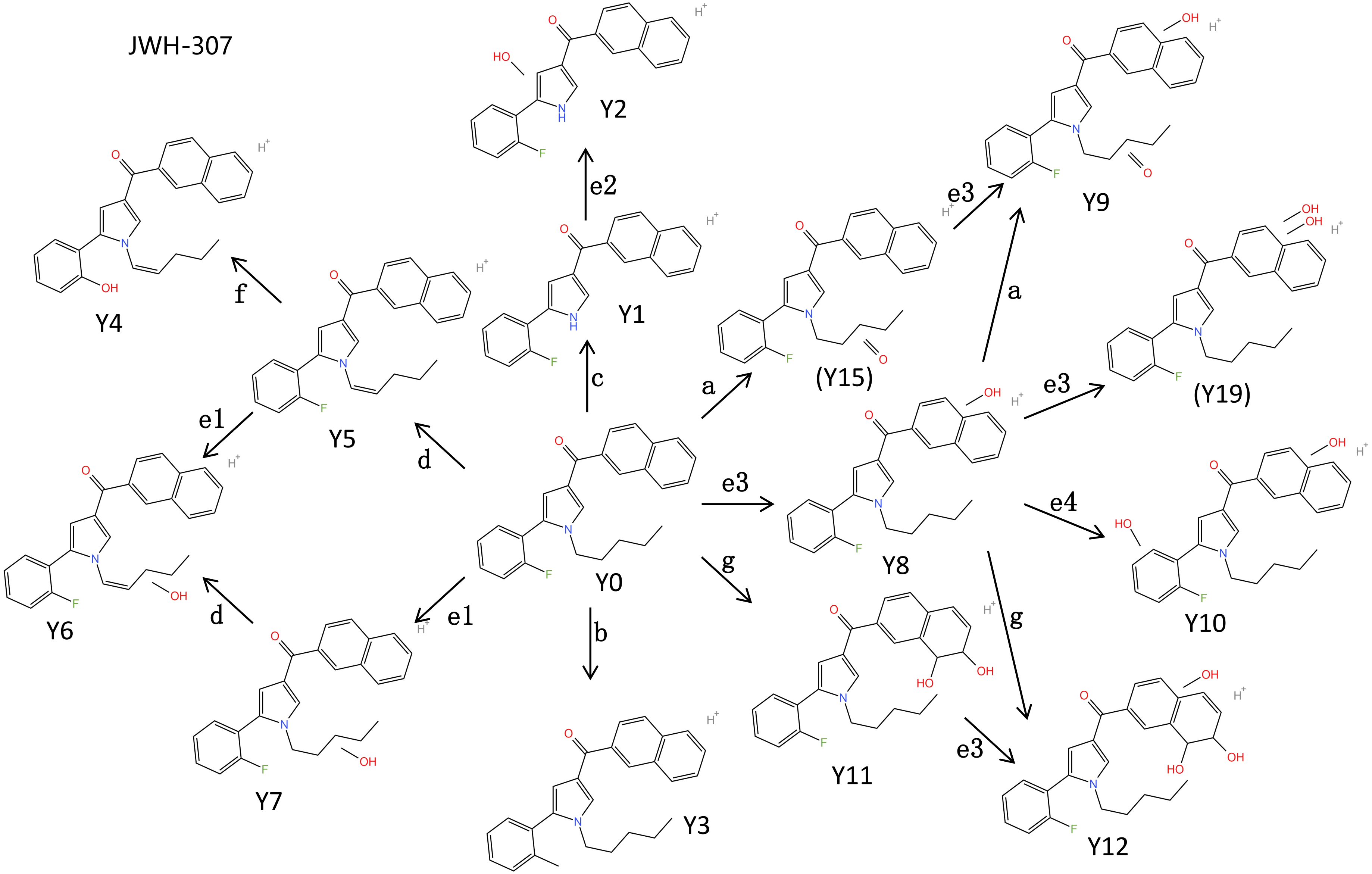

a. ketone formation，b.defluorination，c. N- alkyl side chain removal，d. dehydrogenation，e. hydroxylation（e1. N-alkyl side chains，e2.pyrrole ring，e3. naphthyl，e4.phenyl），f. oxidative defluoridation，g.dihydrodiol

JWH-370

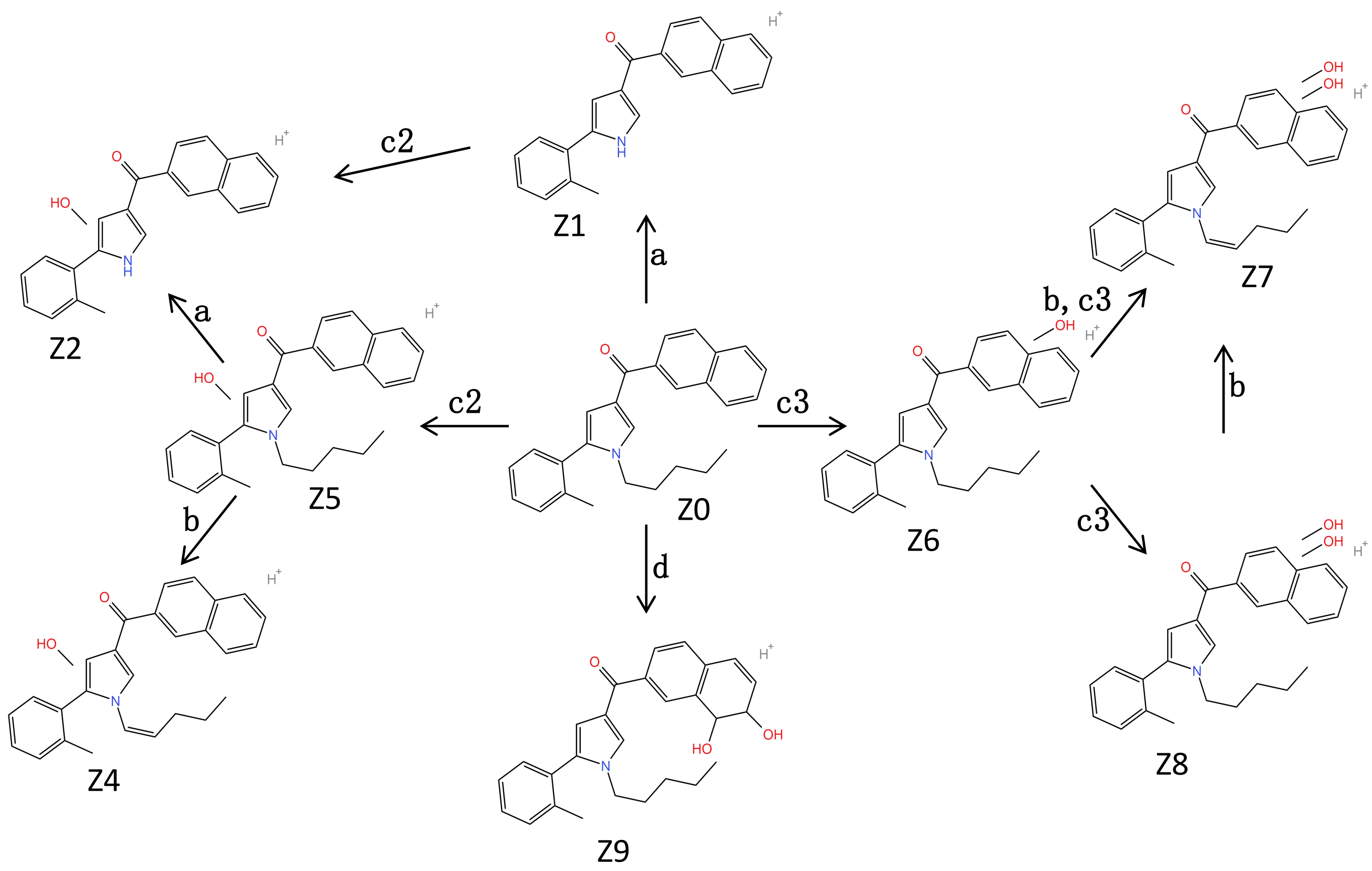

a. N- alkyl side chain removal, b. dehydrogenation, c. hydroxylation (c1. N-alkyl side chains, c2. pyrrole ring, c3. naphthyl), d. dihydrodiol
