# Supplementary material for: Systematic Characterization of In Vitro and In Vivo Metabolic Pathways and Identification of Novel Biomarkers of 26 Synthetic Cannabinoids
Source: Molecules. 2025 Jun 21;30(13):2682. doi: 10.3390/molecules30132682 (PMC12250893; doi:10.3390/molecules30132682)
Supplement: Supplementary file 1 [file molecules-30-02682-s001.zip › Supplementary Materials-Table S1.pdf]

Table1 Information of AB-FUBINACA and it's metabolites

Name	Formula	Transformations	Composition Change	*In vitro* metabolites			*In vivo* metabolites		
				Annot. DeltaMass [ppm]	m/z	RT [min]	Annot. DeltaMass [ppm]	m/z	RT [min]
A0	$C_{20}H_{21}FN_4O_2$	AB-FUBINACA		0.13	369.17218	7.97	-0.88	369.17181	8.17
A1	$C_{13}H_{13}N_3O_2$	Deamination+Dehydrated N- phenyl side chains	-(C7 H8 F N)	0.42	244.10815	5.56			
A2	$C_{15}H_9FN_2O$	Deamidation	-(C5 H12 N2 O)	-2.32	253.07658	8.61	-2.32	253.07658	8.01
A3	$C_{13}H_{16}N_4O_2$	Dehydrated N-phenyl side chains	-(C7 H5 F)	0.39	261.1347	5.56			
A4	$C_{15}H_{11}FN_2O_2$	Amide hydrolysis	-(C5 H10 N2)	-2.24	271.08713	8.61			
A5	$C_{13}H_{16}N_4O_3$	Dehydrated N-phenyl side chains+Hydroxylation （Tert-butyl）	-(C7 H5 F) +(O)	0	277.12952	4.85			
A6	$C_{20}H_{16}FN_3O_2$	Deamination+Dehydrogenation	-(H5 N)	-0.66	350.1297	7.22			
A7	$C_{20}H_{18}FN_3O_2$	Deamination	-(H3 N)	-0.04	352.14557	7.97	-0.77	352.14531	8.17
A8	$C_{20}H_{18}FN_3O_3$	Deamination+Hydroxylation	-(H3 N) +(O)	-1.23	368.14005	8.44			
A9	$C_{20}H_{18}FN_3O_3$	Dehydrogenation+Hydrolysis	-(H3 N) +(O)	-0.98	368.14014	6.96	-2.29	368.13966	7.27
A10	$C_{20}H_{20}FN_3O_3$	Hydrolysis	-(H N) +(O)	-0.46	370.15598	8.61	-1.75	370.1555	8.65
A11	$C_{18}H_{26}N_6O_3$	Dehydrated N-phenyl side chains+Ornithine binding	-(C2 F) +(H5 N2 O)				-1.35	375.21341	7.36
A12	$C_{20}H_{21}FN_4O_3$	Hydroxylation（ Tert- butyl）	+(O)	-1.01	385.16666	6.96			

A13	$C_{20}H_{21}FN_4O_3$	Hydroxylation（N- phenyl side chain）	+(O)	-0.69	385.16678	7.22	-2.64	385.16603	7.27
A14	$C_{20}H_{20}FN_3O_4$	Hydrolysis+Hydroxylation（Tert-butyl）	-(H N) +(O2)	-0.87	386.15073	7.57			
A15	$C_{20}H_{20}FN_3O_4$	Hydrolysis+ Hydroxylation（N-phenyl side chain）	-(H N) +(O2)	-0.71	386.15079	7.81			
A16	$C_{18}H_{26}N_6O_4$	Dehydrated N-phenyl side chains+Hydroxylation+Ornithine binding	-(C2 F) +(H5 N2 O2)				-1.9	391.20809	7.21
A17	$C_{20}H_{21}FN_4O_4$	Dihydroxylation（Tert- butyl）	+(O2)	-0.62	401.16171	6.91			
A18	$C_{20}H_{20}N_4O_6S$	Dehydrogenation+Oxidative defluoridation+Sulfation	-(H F) +(O4 S)				3.66	445.11926	11.68
A19	$C_{26}H_{31}FN_8O_3$	Dehydrogenation+Arginine binding	+(C6 H10 N4 O)				-2.9	523.25608	6.94

Table2 Information of 5F-EMB-PINACA and it's metabolites

Name	Formula	Transformations	Composition Change	*In vitro* metabolites			*In vivo* metabolites		
				Annot. DeltaMass [ppm]	m/z	RT [min]	Annot. DeltaMass [ppm]	m/z	RT [min]
B0	$C_{20}H_{28}FN_3O_3$	5F-EMB-PINACA		-0.47	378.21793	10.11			
B1	$C_{13}H_{14}N_2O_2$	Deamidation+Oxidative defluoridation	-(C7 H14 F N O)				-0.89	231.1126	4.42

B2	$C_{13}H_{13}FN_2O$	Deamidation	-(C7 H15 N O2)	-1.09	233.10822	8.35			
B3	$C_{13}H_{15}FN_2O_2$	Amide hydrolysis	-(C7 H13 N O)	-0.79	251.11884	8.35			
B4	$C_{18}H_{22}FN_3O_3$	Dehydrogenation＋Ester hydrolysis	-(C2 H6)	-1	348.17145	8.17			
B5	$C_{18}H_{24}FN_3O_3$	Ester hydrolysis	-(C2 H4)	-0.81	350.18716	7.20	-2.25	350.18666	8.42
B6	$C_{19}H_{24}FN_3O_3$	Demethylation+Dehydrogenation	-(C H4)	-1.12	362.18704	8.12			
B7	$C_{19}H_{26}FN_3O_3$	Demethylation	-(C H2)	-1.18	364.20267	9.49			
B8	$C_{18}H_{24}FN_3O_4$	Ester hydrolysis＋Hydroxylation（N-alkyl side chains）	-(C2 H4) +(O)	-0.97	366.18201	7.56			
B9	$C_{18}H_{24}FN_3O_4$	Ester hydrolysis＋Hydroxylation（Tert-butyl）	-(C2 H4) +(O)	-0.47	366.18219	6.83			
B10	$C_{20}H_{29}N_3O_4$	Oxidative defluoridation	-(F) +(H O)				-1.14	376.22266	4.99
B11	$C_{18}H_{24}FN_3O_5$	Ester hydrolysis+Dihydroxylation	-(C2 H4) +(O2)				2.72	382.17831	1.14
B12	$C_{20}H_{27}N_3O_5$	Acidification	-(H F) +(O2)	-1.22	390.20187	8.29			
B13	$C_{20}H_{29}N_3O_5$	Oxidative defluoridation+Hydroxylation	-(F) +(H O2)				-1.29	392.21749	4.10
B14	$C_{20}H_{28}FN_3O_4$	Hydroxylation（Tert- butyl）	+(O)	-0.64	394.21341	8.57			
B15	$C_{20}H_{29}N_3O_6$	Oxidative defluoridation+Dihydroxylation	-(F) +(H O3)				-1.48	408.21231	4.06
B16	$C_{20}H_{28}FN_3O_5$	Dihydroxylation	+(O2)				2.94	410.20978	5.31
B17	$C_{20}H_{30}FN_3O_5$	Dihydrodiol	+(H2 O2)				-4.78	412.22226	5.48

B18	$C_{20}H_{28}FN_3O_6$	Trihydroxylation	+(O3)				-3.8	426.20187	4.84
B19	$C_{20}H_{30}FN_3O_7$	Dihydrodiol+Dihydroxylation	+(H2 O4)				3.29	444.21551	6.58
B20	$C_{20}H_{26}FN_3O_7S$	Ketone formation+Sulfation	-(H2) +(O4 S)				3.98	472.1567	1.03
B21	$C_{24}H_{32}FN_3O_9$	Ester hydrolysis+ Glucuronidation	+(C4 H4 O6)	-0.33	526.21936	7.21			
B22	$C_{26}H_{40}FN_7O_4$	Arginine binding	+(C6 H12 N4 O)				-3.66	534.3179	8.37
B23	$C_{26}H_{38}FN_7O_5$	Ketone formation+Arginine binding	+(C6 H10 N4 O2)				-2.99	548.29749	10.19

Table3 Information of AB-4en-PINACA and it's metabolites

Name	Formula	Transformations	Composition Change	*In vitro* metabolites			*In vivo* metabolites		
				Annot. DeltaMass [ppm]	m/z	RT [min]	Annot. DeltaMass [ppm]	m/z	RT [min]
C0	$C_{18}H_{24}N_4O_2$	AB-4en-PINACA		-0.64	329.19699	RT [min]	Annot. DeltaMass [ppm]	m/z	RT [min]
C1	$C_{13}H_{12}N_2O$	Deamidation	-(C5 H12 N2 O)	-0.46	213.10214	7.42	-1.77	329.19662	8.15
C2	$C_{13}H_{14}N_2O_2$	Amide hydrolysis	-(C5 H10 N2)	-0.25	231.11275	7.20	-0.47	213.10214	8.32
C3	$C_{13}H_{14}N_2O_3$	Amide hydrolysis+ Hydroxylation	-(C5 H10 N2) +(O)	-0.09	247.1077	8.78	-0.73	231.11264	5.37

C4	$C_{13}H_{16}N_2O_4$	Amide hydrolysis+ Dihydrodiol	-(C5 H8 N2) +(O2)	-0.22	265.11823	6.32			
C5	$C_{18}H_{21}N_3O_2$	Deamination	-(H3 N)	-0.49	312.1705	5.42			
C6	$C_{18}H_{21}N_3O_3$	Deamination+Hydroxylation（Tert-butyl）	-(H3 N) +(O)	-0.78	328.16531	8.06	-2.06	312.17001	8.15
C7	$C_{18}H_{23}N_3O_3$	Hydrolysis	-(H N) +(O)	-0.94	330.18091	7.20	-2.06	328.16489	6.42
C8	$C_{18}H_{24}N_4O_3$	Hydroxylation（N-alkyl side chains）	+(O)	-0.74	345.19186	7.52			
C9	$C_{18}H_{24}N_4O_3$	Hydroxylation（Tert- butyl）	+(O)	-0.47	345.19196	7.21			
C10	$C_{18}H_{23}N_3O_4$	Deamination＋Dihydrodiol	-(H N) +(O2)	-0.49	346.17596	6.09			
C11	$C_{18}H_{23}N_3O_4$	Hydrolysis+Hydroxylation	-(H N) +(O2)	-0.22	346.17606	6.96			
C12	$C_{18}H_{26}N_4O_3$	Hydration	+(H2 O)	-0.27	347.20767	5.42			
C13	$C_{18}H_{26}N_4O_4$	Dihydrodiol	+(H2 O2)			6.22			
C14	$C_{18}H_{25}N_3O_5$	Hydrolysis+Dihydrodiol	-(N) +(H O3)				-1.16	363.20226	5.53
C15	$C_{18}H_{24}N_4O_5$	Trihydroxylation	+(O3)				-1.66	364.1861	4.23
C16	$C_{23}H_{32}N_6O_3$	Dehydrogenation+Ornithine binding	+(C5 H8 N2 O)				-1.9	377.18123	4.19

Table4 Information of ADB-4en-PINACA and it's metabolites

Name	Formula	Transformations	CompositionChange	Invitrometabolites			Invivometabolites		
				Annot.DeltaMass[ppm]	m/z	RT [min]	Annot. DeltaMass [ppm]	m/z	RT [min]
D0	$C_{19}H_{26}N_4O_2$	ADB-4en-PINACA		-0.33	343.21274	8.63			

D1	$C_{13}H_{12}N_2O$	Deamidation	-(C6 H14 N2 O)	-0.39	213.10216	8.63	-0.75	213.10208	7.70
D2	$C_{13}H_{12}N_2O_2$	Deamidation+ Hydroxylation（N-alkyl side chains）	-(C6 H14 N2)	-0.06	229.09714	6.83	0.19	229.0972	4.25
D3	$C_{13}H_{14}N_2O_2$	Amide hydrolysis	-(C6 H12 N2)	-0.31	231.11273	8.63	-0.61	231.11266	4.45
D4	$C_{13}H_{14}N_2O_3$	Deamidation+Dihydrodiol	-(C6 H12 N2) +(O)				-1.41	247.10737	4.06
D5	$C_{13}H_{16}N_2O_3$	Amide hydrolysis+Hydration	-(C6 H10 N2) +(O)				-0.62	249.12321	3.60
D6	$C_{13}H_{15}N_3O_3$	Deamidation（C-N bond at amide junction is broken）+Hydration	-(C6 H11 N) +(O)				-0.57	262.11847	4.04
D7	$C_{13}H_{16}N_2O_4$	Dihydrodiol＋Amide hydrolysis	-(C6 H10 N2) +(O2)	0.01	265.11829	5.87			
D8	$C_{19}H_{23}N_3O_2$	Deamination	-(H3 N)	-0.36	326.18619	8.63			
D9	$C_{19}H_{24}N_4O_2$	Dehydrogenation	-(H2)	-0.26	341.19711	8.31			
D10	$C_{19}H_{23}N_3O_3$	Deamination+Hydroxylation（Tert-butyl）	-(H3 N) +(O)	-0.82	342.18094	7.40			
D11	$C_{19}H_{23}N_3O_3$	Deamination+Hydroxylation（N-alkyl side chains）	-(H3 N) +(O)	-0.1	342.18118	6.81			
D12	$C_{19}H_{25}N_3O_3$	Hydration＋Deamination	-(H N) +(O)	-0.71	344.19662	9.27	3.91	344.19821	9.48
D13	$C_{19}H_{22}N_4O_3$	Dehydrogenation+Ketone formation	-(H4) +(O)				1.83	355.17711	10.47
D14	$C_{19}H_{26}N_4O_3$	Hydroxylation（Tert- butyl）	+(O)	-1.2	359.20734	7.40			

D15	$C_{19}H_{26}N_4O_3$	Hydroxylation（Indazole ring）	+(O)	-0.78	359.20749	7.53			
D16	$C_{19}H_{26}N_4O_3$	Hydroxylation（N-alkyl side chains）	+(O)	-0.52	359.20758	6.58			
D17	$C_{19}H_{25}N_3O_4$	Dihydrodiol＋Deamination	-(H N) +(O2)	-0.2	360.19171	5.87	-1.98	360.19107	5.90
D18	$C_{19}H_{28}N_4O_3$	Hydration	+(H2 O)	-0.59	361.22321	6.70	-4.38	361.22184	10.74
D19	$C_{19}H_{26}N_4O_4$	Dihydroxylation	+(O2)				-0.61	375.20245	4.96
D20	$C_{19}H_{25}N_3O_5$	Hydrolysis+Dihydroxylation	-(H N) +(O3)				-0.6	376.18647	4.96
D21	$C_{19}H_{28}N_4O_4$	Dihydrodiol	+(H2 O2)	-0.02	377.21832	5.87			
D22	$C_{19}H_{27}N_3O_5$	Hydrolysis+Dihydrodiol	-(N) +(H O3)				-1.34	378.20184	3.76
D23	$C_{19}H_{26}N_4O_5$	Trihydroxylation	+(O3)				3.38	391.19891	1.01
D24	$C_{19}H_{28}N_4O_5$	Hydroxylation+Dihydrodiol	+(H2 O3)				-1.39	393.2127	4.68
D25	$C_{19}H_{28}N_4O_6$	Dihydroxylation+Dihydrodiol	+(H2 O4)				-1.35	409.20761	4.29
D26	$C_{21}H_{30}N_4O_5$	Dihydrodiol+Acetylization	+(C2 H4 O3)				-1.39	419.22831	5.51
D27	$C_{21}H_{30}N_4O_5$	Dihydrodiol+Acetylization	+(C2 H4 O3)				-1.31	419.22835	4.32
D28	$C_{24}H_{34}N_6O_3$	Dehydrogenation+Ornithine binding	+(C5 H8 N2 O)				-0.68	455.27621	11.52
D29	$C_{24}H_{36}N_6O_3$	Ornithine binding	+(C5 H10 N2 O)				-0.96	457.29173	10.61
D30	$C_{25}H_{40}N_8O_4$	Hydration+Arginine binding	+(C6 H14 N4 O2)				4.02	517.3266	6.97

Table5 Information of ADB-CHMINACA and it's metabolites

Name	Formula	Transformations	Composition Change	*In vitro* metabolites			*In vivo* metabolites		
				Annot. DeltaMass [ppm]	m/z	RT [min]	Annot. DeltaMass [ppm]	m/z	RT [min]
E0	$C_{21}H_{30}N_4O_2$	ADB-CHMINACA		-0.53	371.24396	9.80	-1.85	371.24347	9.74
E1	$C_{15}H_{16}N_2O$	Deamidation	-(C6 H14 N2 O)	-3.35	241.13274	8.65	-1.06	241.13329	7.29
E2	$C_{15}H_{16}N_2O_2$	Deamidation+Hydroxylation（N-alkyl side chains）	-(C6 H14 N2)	-2.05	257.12793	7.90	-0.5	257.12833	4.45
E3	$C_{15}H_{16}N_2O_3$	Deamidation+Dihydroxylation	-(C6 H14 N2) +(O)				-0.42	273.12325	5.15
E4	$C_{15}H_{18}N_2O_3$	Amide hydrolysis+Hydroxylation（N-alkyl side chains）	-(C6 H12 N2) +(O)	-1.27	275.13867	7.89	-0.51	275.13888	4.45
E5	$C_{15}H_{18}N_2O_4$	Amide hydrolysis+Dihydroxylation	-(C6 H12 N2) +(O2)				1.33	291.13432	1.00
E6	$C_{21}H_{27}N_3O_2$	Deamination	-(H3 N)	-0.64	354.21738	9.80	-2.46	354.21674	9.69
E7	$C_{21}H_{27}N_3O_3$	Deamination+Hydroxylation（N-alkyl side chains）	-(H3 N) +(O)	-1.39	370.21201	7.62	-1.52	370.21196	7.00
E8	$C_{21}H_{29}N_3O_3$	Hydrolysis	-(H N) +(O)	-0.46	372.228	10.41	4.39	372.2298	4.24
E9	$C_{21}H_{28}N_4O_3$	Hydrolysis+Hydroxylation（Tert-butyl）	-(H2) +(O)	-1.5	385.22284	8.65			

E10	$C_{21}H_{28}N_4O_4$	Hydrolysis+Hydroxylation（N-alkyl side chains）	-(H2) +(O)	-0.95	385.22305	7.39			
E11	$C_{21}H_{27}N_3O_4$	Deamination+Dihydroxylation（N-alkyl side chains+Tert-butyl）	-(H3 N) +(O2)	-0.96	386.20706	6.87	-1.89	386.20671	6.51
E12	$C_{21}H_{30}N_4O_3$	Hydroxylation（N-alkyl side chains）	+(O)	-1.48	387.23849	7.62			
E13	$C_{21}H_{30}N_4O_4$	Dihydroxylation（Tert-butyl+N-alkyl side chains）	+(O2)	-1.15	403.23352	7.11	-1.69	403.2333	6.51
E14	$C_{21}H_{30}N_4O_4$	Dihydroxylation（Tert-butyl）	+(O2)	-0.16	403.23392	6.06	-1.39	403.23342	6.03
E15	$C_{21}H_{32}N_4O_4$	Dihydrodiol	+(H2 O2)				-0.9	405.24927	7.48
E16	$C_{21}H_{31}N_3O_5$	Hydrolysis+Dihydrodiol	-(N) +(H O3)				-1.07	406.23322	5.72
E17	$C_{21}H_{30}N_4O_5$	Trihydroxylation	+(O3)				-1.19	419.2284	5.51
E18	$C_{21}H_{32}N_4O_5$	Hydroxylation+Dihydrodiol	+(H2 O3)				-0.31	421.24442	4.86
E19	$C_{26}H_{40}N_6O_3$	Ornithine binding	+(C5 H10 N2 O)				2.89	485.32486	8.00
E20	$C_{26}H_{40}N_6O_4$	Ornithine binding+Hydroxylation	+(C5 H10 N2 O2)				-4.27	501.31624	9.11
E21	$C_{27}H_{39}N_3O_6$	Decarbonyl+Glucuronidation	-(N) +(C6 H9 O4)	2.26	502.2923	9.67			
E22	$C_{26}H_{42}N_6O_5$	Ornithine binding+Dihydrodiol	+(C5 H12 N2 O3)	-4.74	519.32649	9.19			

E23	$C_{27}H_{41}N_7O_4$	Hydrolysis+Arginine binding	+(C6 H11 N3 O2)	-1.15	528.32867	11.97			
E24	$C_{27}H_{41}N_7O_6$	Hydrolysis+Dihydroxylation +Arginine binding	+(C6 H11 N3 O4)	0.08	560.31915	8.17			
E25	$C_{27}H_{38}N_4O_{10}$	Dihydroxylation+Glucuronidation	+(C6 H8 O8)	2.82	579.2677	11.00			

Table6 Information of ADB-HEXINACA and it's metabolites

Name	Formula	Transformations	Composition Change	*In vitro* metabolites			*In vivo* metabolites		
				Annot. DeltaMass [ppm]	m/z	RT [min]	Annot. DeltaMass [ppm]	m/z	RT [min]
F0	$C_{20}H_{30}N_4O_2$	ADB-HEXINACA		-1.23	359.24371	9.70			
F1	$C_{14}H_{16}N_2O$	Deamidation	-(C6 H14 N2 O)	-1.38	229.13322	9.70			
F2	$C_{14}H_{14}N_2O_2$	Deamidation＋Ketone formation	-(C6 H16 N2)	-0.99	243.11256	7.29			
F3	$C_{14}H_{16}N_2O_2$	Deamidation+Hydroxylation（N-alkyl side chains）	-(C6 H14 N2)	-0.9	245.12823	7.05			
F4	$C_{14}H_{18}N_2O_2$	Amide hydrolysis	-(C6 H12 N2)	-0.99	247.14386	9.70	-1.06	247.14384	5.19
F5	$C_{14}H_{18}N_2O_3$	Amide hydrolysis+ Hydroxylation（N-alkyl side chains）	-(C6 H12 N2) +(O)	-0.74	263.13882	7.47	-0.93	263.13877	5.12
F6	$C_{14}H_{18}N_2O_4$	Amide hydrolysis+Dihydroxylation	-(C6 H12 N2) +(O2)				0.21	279.13399	4.38
F7	$C_{20}H_{27}N_3O_2$	Deamination	-(H3 N)	-0.75	342.21735	9.70			

F8	$C_{20}H_{25}N_3O_3$	Deamination＋Ketone formation	-(H5 N) +(O)	-1.46	356.19635	7.29			
F9	$C_{20}H_{27}N_3O_3$	Deamination+ Hydroxylation（Tert- butyl）	-(H3 N) +(O)	-1.18	358.2121	7.47			
F10	$C_{20}H_{29}N_3O_3$	Hydrolysis	-(H N) +(O)	-1.58	360.2276	10.29			
F11	$C_{20}H_{28}N_4O_3$	Ketone formation	-(H2) +(O)	-1.22	373.22296	7.29			
F12	$C_{20}H_{30}N_4O_3$	Hydroxylation（Tert- butyl）	+(O)	-0.96	375.23871	7.47			
F13	$C_{20}H_{30}N_4O_3$	Hydroxylation（N - alkyl side chains）	+(O)	-0.88	375.23874	7.19			
F14	$C_{20}H_{30}N_4O_3$	Hydroxylation(Indazole ring）	+(O)	-0.8	375.23877	8.59			
F15	$C_{20}H_{29}N_3O_4$	Hydrolysis+Hydroxylation	-(H N) +(O2)				0	376.22308	4.96
F16	$C_{20}H_{31}N_3O_4$	Hydrolysis+Dihydrodiol	-(N) +(H O2)				-1.27	378.23826	5.32
F17	$C_{20}H_{28}N_4O_4$	Acidification	-(H2) +(O2)				-1.25	389.21785	5.26
F18	$C_{20}H_{30}N_4O_4$	Dihydroxylation（N - alkyl side chains）	+(O2)	-1.34	391.23346	6.51			
F19	$C_{20}H_{30}N_4O_4$	Dihydroxylation(Indazole ring+N - alkyl side chains)	+(O2)	-0.79	391.23367	6.05			
F20	$C_{20}H_{29}N_3O_5$	Hydrolysis+Dihydroxylation	-(H N) +(O3)				-1.85	392.21727	4.25
F21	$C_{20}H_{32}N_4O_4$	Dihydrodiol	+(H2 O2)				-1.57	393.24902	3.68
F22	$C_{20}H_{31}N_3O_5$	Hydrolysis+Dihydrodiol	-(N) +(H O3)				-1.05	394.23324	4.64
F23	$C_{20}H_{30}N_4O_5$	Trihydroxylation	+(O3)				-1.36	407.22834	5.03
F24	$C_{20}H_{29}N_3O_6$	Hydrolysis+Trihydroxylation	-(H N) +(O4)				-1.02	408.2125	4.86
F25	$C_{22}H_{32}N_4O_4$	Hydroxylation+Acetylization	+(C2 H2 O2)				-1.9	417.24884	6.04

F26	$C_{25}H_{40}N_6O_3$	Ornithine binding	+(C5 H10 N2 O)				-1.19	473.3229	9.84
F27	$C_{25}H_{38}N_6O_4$	Glutamine binding	+(C5 H8 N2 O2)				-2.13	487.3017	11.01
F28	$C_{25}H_{40}N_6O_4$	Hydroxylation+Ornithine binding	+(C5 H10 N2 O2)				-0.92	489.31793	7.18
F29	$C_{25}H_{38}N_6O_5$	Hydroxylation+Glutamine binding	+(C5 H8 N2 O3)				-4.89	503.29519	9.82
F30	$C_{25}H_{40}N_6O_5$	Dihydroxylation+Ornithine binding	+(C5 H10 N2 O3)				-4.68	505.31094	10.19
F31	$C_{26}H_{38}N_4O_9$	Hydroxylation(Indazole ring)＋Glucuronidation	+(C6 H8 O7)	-0.73	551.27075	6.62			

Table7 Information of EDMB-PINACA and it's metabolites

Name	Formula	Transformations	Composition Change	*In vitro* metabolites			*In vivo* metabolites		
				Annot. DeltaMass [ppm]	m/z	RT [min]	Annot. DeltaMass [ppm]	m/z	RT [min]
G0	$C_{21}H_{31}N_3O_3$	EDMB-PINACA		-0.04	374.2438	12.03	-0.94	374.24347	11.60
G1	$C_{13}H_{14}N_2O$	Deamidation	-(C8 H17 N O2)	-1.85	215.11749	10.30			
G2	$C_{13}H_{12}N_2O_2$	Deamidation＋Ketone formation	-(C8 H19 N O)	-0.54	229.09703	8.23	-0.06	229.09714	4.26
G3	$C_{13}H_{14}N_2O_2$	Deamidation+ Hydroxylation(N-alkyl side chains)	-(C8 H17 N O)	-0.12	231.11278	7.84	-0.33	231.11273	4.31
G4	$C_{13}H_{16}N_2O_2$	Amide hydrolysis	-(C8 H15 N O)	-1.01	233.12822	10.31			

G5	$C_{13}H_{14}N_2O_3$	Deamidation+Dihydroxylation	-(C8 H17 N)				-0.89	247.1075	4.07
G6	$C_{13}H_{16}N_2O_3$	Amide hydrolysis+ Hydroxylation(N-alkyl side chains)	-(C8 H15 N)	-0.38	249.12328	8.12			
G7	$C_{13}H_{18}N_2O_4$	Amide hydrolysis+Dihydrodiol	-(C8 H13 N) +(O)				-1.46	267.13354	1.78
G8	$C_{16}H_{19}N_3O_3$	N- alkyl side chain removal+Dehydrogenation	-(C5 H12)				0.19	302.14998	4.23
G9	$C_{16}H_{21}N_3O_3$	N- alkyl side chain removal	-(C5 H10)	-0.74	304.16534	9.08	-0.18	304.16551	4.42
G10	$C_{18}H_{25}N_3O_2$	Decarbonyl+ Hydroxylation(N-alkyl side chains)	-(C3 H6 O)	-0.45	316.20181	7.84			
G11	$C_{19}H_{25}N_3O_2$	Deester group removal	-(C2 H6 O)	-0.9	328.20166	10.30			
G12	$C_{16}H_{21}N_3O_5$	N- alkyl side chain removal+Dihydroxylation	-(C5 H10) +(O2)				-0.91	336.15509	3.42
G13	$C_{19}H_{23}N_3O_3$	Deester group removal+ Ketone formation	-(C2 H8)	-0.55	342.18103	7.69			
G14	$C_{19}H_{25}N_3O_3$	Deester group removal+ Hydroxylation(N-alkyl side chains)	-(C2 H6)	-0.53	344.19669	10.24			
G15	$C_{19}H_{27}N_3O_3$	Ester hydrolysis	-(C2 H4)	-0.95	346.21219	10.31	-1.48	346.21201	9.79
G16	$C_{19}H_{25}N_3O_4$	Ester hydrolysis＋Ketone formation	-(C2 H6) +(O)	-1.73	360.19116	8.56	-0.88	360.19147	7.72
G17	$C_{19}H_{27}N_3O_4$	Ester hydrolysis+ Hydroxylation	-(C2 H4) +(O)	-1.11	362.20703	8.61	-2.55	362.20651	7.32

G18	$C_{19}H_{25}N_3O_5$	Ester hydrolysis+ Acidification	-(C2 H6) +(O2)	-0.63	376.18646	7.19			
G19	$C_{19}H_{27}N_3O_5$	Deester group removal+ Dihydroxylation(N-alkyl side chains+Tert-butyl)	-(C2 H4) +(O2)	-0.53	378.20215	6.97	-0.8	378.20205	3.77
G20	$C_{19}H_{27}N_3O_5$	Deester group removal+ Dihydroxylation(Indazole ring+N-alkyl side chains)	-(C2 H4) +(O2)	-0.45	378.20218	6.70			
G21	$C_{19}H_{29}N_3O_5$	Ester hydrolysis+Dihydrodiol	-(C2 H2) +(O2)				-0.59	380.21777	5.09
G22	$C_{21}H_{29}N_3O_4$	Ketone formation	-(H2) +(O)	-0.79	388.22278	9.97			
G23	$C_{21}H_{31}N_3O_4$	Hydroxylation(N-alkyl side chains)	+(O)	-1.32	390.23822	10.19			
G24	$C_{21}H_{31}N_3O_4$	Hydroxylation(Tert-butyl)	+(O)	-1.08	390.23831	9.86			
G25	$C_{21}H_{29}N_3O_5$	Ketone formation+Hydroxylation(Tert-butyl)	-(H2) +(O2)	-1.16	404.21768	8.25			
G26	$C_{21}H_{31}N_3O_5$	Dihydroxylation(Tert-butyl+ N-alkyl side chains)	+(O2)	-0.77	406.23334	8.45			
G27	$C_{21}H_{31}N_3O_5$	Dehydrogenation+Dihydrodiol	+(O2)	-0.39	406.23349	7.86			
G28	$C_{21}H_{33}N_3O_5$	Dihydrodiol	+(H2 O2)	-0.82	408.24896	9.23	2.5	408.25031	9.11
G29	$C_{23}H_{33}N_3O_4$	Acetylization	+(C2 H2 O)				-1.24	416.25387	6.67
G30	$C_{21}H_{31}N_3O_6$	Trihydroxylation	+(O3)				-2.14	422.22766	5.93

G31	$C_{19}H_{27}N_3O_6S$	Ester hydrolysis+Sulfation	-(C2 H4) +(O3 S)				-1.2	426.16882	5.29
G32	$C_{23}H_{33}N_3O_6$	Acetylization+Dihydroxylation	+(C2 H2 O3)				-3.49	448.24265	9.11
G33	$C_{25}H_{39}N_7O_4$	Ester hydrolysis+Arginine binding	+(C4 H8 N4 O)				-0.5	502.31338	7.04
G34	$C_{26}H_{37}N_5O_6$	Ketone formation+Glutamine binding	+(C5 H6 N2 O3)				-3.52	516.27985	5.89
G35	$C_{26}H_{41}N_5O_6$	Ornithine binding+Dihydroxylation	+(C5 H10 N2 O3)				0.24	520.31308	5.54
G36	$C_{27}H_{43}N_7O_4$	Arginine binding	+(C6 H12 N4 O)				3.42	530.34674	11.76
G37	$C_{26}H_{39}N_5O_7$	Glutamine binding+Dihydroxylation	+(C5 H8 N2 O4)				4.48	534.29462	10.40
G38	$C_{26}H_{39}N_5O_7$	Glutamine binding+Dihydroxylation	+(C5 H8 N2 O4)				4.63	534.29469	10.40
G39	$C_{26}H_{39}N_5O_7$	Dihydroxylation+Glutamine binding	+(C5 H8 N2 O4)				4.83	534.2948	10.33
G40	$C_{26}H_{41}N_5O_7$	Dihydrodiol+Glutamine binding	+(C5 H10 N2 O4)				-0.48	536.30762	5.18
G41	$C_{27}H_{39}N_3O_{10}$	Hydroxylation(Tert-butyl)+ Glucuronidation	+(C6 H8 O7)	-0.77	566.27039	8.06			
G42	$C_{27}H_{39}N_3O_{10}$	Hydroxylation(N-alkyl side chains)＋Glucuronidation	+(C6 H8 O7)	-0.66	566.27045	8.61			

Table8 Information of EMB-FUBINACA and it's metabolites

Name	Formula	Transformations	Composition Change	*In vitro* metabolites			*In vivo* metabolites		
				Annot. DeltaMass [ppm]	m/z	RT [min]	Annot. DeltaMass [ppm]	m/z	RT [min]
H0	$C_{22}H_{24}FN_3O_3$	EMB-FUBINACA		-0.71	398.18716	10.29			
H1	$C_{15}H_9FN_2O$	Deamidation	-(C7 H15 N O2)	-2.07	253.07664	8.72			
H2	$C_{15}H_{11}FN_2O_2$	Amide hydrolysis	-(C7 H13 N O)	-1	271.08746	8.72			
H3	$C_{16}H_{13}FN_2O_2$	Amide hydrolysis+Ketone formation	-(C6 H11 N O)	-0.5	285.10324	8.72			
H4	$C_{15}H_{19}N_3O_4$	Hydroxylation+N- alkyl side chain removal	-(C7 H5 F) +(O)				-1.39	306.14441	3.00
H5	$C_{15}H_{21}N_3O_5$	Dihydrodiol+N- alkyl side chain removal	-(C7 H3 F) +(O2)				-1.38	324.15495	3.62
H6	$C_{20}H_{20}FN_3O_3$	Ester hydrolysis	-(C2 H4)	-0.79	370.15585	7.56	-2.19	370.15534	8.67
H7	$C_{20}H_{20}FN_3O_4$	Ester hydrolysis+Hydroxylation（N-alkyl side chains）	-(C2 H4) +(O)	-1.03	386.15067	7.90			
H8	$C_{20}H_{20}FN_3O_4$	Ester hydrolysis+Hydroxylation(Tert-butyl)	-(C2 H4) +(O)	-0.79	386.15076	7.51			
H9	$C_{22}H_{27}N_3O_6$	Oxidative defluoridation+Dihydrodiol	-(F) +(H3 O3)				-1.2	430.19675	4.02

H10	$C_{24}H_{28}FN_3O_6$	Dihydrodiol+Acetylization	+(C2 H4 O3)				-2.34	474.20238	6.75
H11	$C_{27}H_{34}FN_5O_4$	Ornithine binding	+(C5 H10 N2 O)				0.28	512.2669	6.27
H12	$C_{26}H_{32}FN_7O_4$	Ester hydrolysis+Arginine binding	+(C4 H8 N4 O)				-0.24	526.25713	6.91
H13	$C_{26}H_{28}FN_3O_9$	Ester hydrolysis+Glucuronidation	+(C4 H4 O6)	-0.56	546.18793	7.57			
H14	$C_{28}H_{36}FN_7O_5$	Hydroxylation+Ornithine binding	+(C6 H12 N4 O2)	-4.46	570.28093	9.64			

Table9 Information of ADB-3en-BUTINACA and it's metabolites

Name	Formula	Transformations	Composition Change	*In vitro* metabolites			*In vivo* metabolites		
				Annot. DeltaMass [ppm]	m/z	RT [min]	Annot. DeltaMass [ppm]	m/z	RT [min]
I0	$C_{18}H_{24}N_4O_2$	ADB-3en-BUTINACA		-1.2	329.19681	8.26			
I1	$C_{12}H_{10}N_2O$	Deamidation	-(C6 H14 N2 O)				-0.51	199.08649	8.29
I2	$C_{12}H_{12}N_2O_2$	Amide hydrolysis	-(C6 H12 N2)	-0.64	217.09702	8.27			
I3	$C_{13}H_{15}N_3O$	N- alkyl side chain removal+Decarbonyl	-(C5 H9 N O)	0.25	230.12885	6.31			
I4	$C_{13}H_{14}N_2O_2$	Amide hydrolysis+Ketone formation	-(C5 H10 N2)	-0.98	231.11258	8.27			

I5	$C_{12}H_{14}N_2O_4$	Amide hydrolysis+Hydroxylation（N-alkyl side chains）+Hydration	-(C6 H10 N2) +(O2)	-0.32	251.10255	5.87	-1.03	251.10238	4.30
I6	$C_{14}H_{18}N_4O_2$	N- alkyl side chain removal	-(C4 H6)	-0.39	275.15015	6.31			
I7	$C_{18}H_{21}N_3O_2$	Deamination	-(H3 N)	-1.17	312.17029	8.26	-1.04	312.17033	8.29
I8	$C_{18}H_{21}N_3O_3$	Deamination+Hydroxylation（Indazole ring)	-(H3 N) +(O)	-0.87	328.16528	6.51			
I9	$C_{18}H_{21}N_3O_3$	Deamination+Hydroxylation(N-alkyl side chains）	-(H3 N) +(O)	-0.22	328.1655	6.80	-1.97	328.16492	6.78
I10	$C_{18}H_{23}N_3O_3$	Hydrolysis	-(H N) +(O)	-0.94	330.18091	8.94			
I11	$C_{18}H_{24}N_4O_3$	Hydroxylation(N-alkyl side chains）	+(O)	-1.09	345.19174	7.47			
I12	$C_{18}H_{24}N_4O_3$	Hydroxylation（Indazole ring)	+(O)	-0.82	345.19183	6.51			
I13	$C_{18}H_{23}N_3O_4$	Deamination+Dihydrodiol	-(H N) +(O2)	-0.49	346.17596	5.87	-1.41	346.17565	5.73
I14	$C_{18}H_{26}N_4O_4$	Dihydrodiol	+(H2 O2)	-0.38	363.20255	5.87	-1.56	363.20212	5.74
I15	$C_{18}H_{25}N_3O_5$	Hydrolysis+Dihydrodiol	-(N) +(H O3)				-1.48	364.18616	4.25
I16	$C_{18}H_{24}N_4O_5$	Trihydroxylation	+(O3)				-1.65	377.18132	4.19
I17	$C_{18}H_{23}N_3O_6$	Hydrolysis+Trihydroxylation	-(H N) +(O4)				0.39	378.16611	4.13
I18	$C_{18}H_{26}N_4O_5$	Dihydrodiol+Hydroxylation	+(H2 O3)				-1.43	379.19705	4.48
I19	$C_{23}H_{32}N_6O_3$	Dehydrogenation+Ornithine binding	+(C5 H8 N2 O)				-1.82	441.26007	10.33
I20	$C_{23}H_{34}N_6O_3$	Ornithine binding	+(C5 H10 N2				0.63	443.27679	8.59

			O)						
I21	$C_{23}H_{34}N_6O_4$	Hydroxylation+Ornithine binding	+(C5 H10 N2 O2)				-3.63	459.26977	9.24
I22	$C_{24}H_{32}N_4O_9$	Hydroxylation（Indazole ring)+Glucuronidation	+(C6 H8 O7)	-0.04	521.22418	5.86			

Table10 Information of 5F-ADB and it's metabolites

Name	Formula	Transformations	Composition Change	*In vitro* metabolites			*In vivo* metabolites		
				Annot. DeltaMass [ppm]	m/z	RT [min]	Annot. DeltaMass [ppm]	m/z	RT [min]
J0	$C_{20}H_{28}FN_3O_3$	5F-ADB		-0.66	378.21899	10.03	-1.26	378.21827	10.05
J1	$C_{13}H_{14}N_2O_2$	Deamidation+Oxidative defluoridation	-(C7 H14 F N O)	-1.17	231.11307	7.20	0.12	231.11283	3.75
J2	$C_{13}H_{13}FN_2O$	Deamidation	-(C7 H15 N O2)	-0.56	233.1086	8.68			
J3	$C_{13}H_{15}FN_2O_2$	Amide hydrolysis	-(C7 H13 N O)	-0.84	251.11924	8.68			
J4	$C_{13}H_{16}N_2O_4$	Amide hydrolysis+Oxidative defluoridation+Hydroxylation	-(C7 H12 F N) +(O)				-0.28	265.11821	4.60
J5	$C_{15}H_{19}N_3O_3$	N- alkyl side chain removal	-(C5 H9 F)	-0.86	290.15017	7.86			
J6	$C_{19}H_{27}N_3O_4$	Oxidative defluoridation+Ester hydrolysis	-(C H F) +(O)				1.37	362.20793	1.02
J7	$C_{19}H_{26}FN_3O_3$	Ester hydrolysis	-(C H2)	-0.41	364.20325	8.68			
J8	$C_{20}H_{29}N_3O_4$	Oxidative defluoridation	-(F) +(H O)				-0.41	376.22293	4.98

J9	$C_{20}H_{29}N_3O_4$	Dehydrogenation	-(H2)	-0.69	376.22334	8.28			
J10	$C_{18}H_{26}FN_5O_3$	Ester hydrolysis+Hydroxylation	-(C H2)+(O)	-0.76	380.20953	7.21	-1.61	380.1974	7.60
J11	$C_{20}H_{27}N_3O_5$	Acidification	-(H F) +(O2)	-0.26	390.20245	8.17	-2.06	390.20155	8.31
J12	$C_{20}H_{29}N_3O_5$	Oxidative defluoridation+Hydroxylation	-(F) +(H O2)				-0.45	392.21782	4.13
J13	$C_{20}H_{29}N_3O_5$	Ketone formation	-(H2)+(O)	-0.74	392.21828	7.20			
J14	$C_{20}H_{26}FN_3O_5$	Ketone formation+Hydroxylation	-(H2) +(O2)				-1.75	408.19221	8.30
J15	$C_{20}H_{29}N_3O_6$	Oxidative defluoridation+Dihydroxylation	-(F) +(H O3)				-1.03	408.21249	4.97
J16	$C_{22}H_{30}FN_3O_4$	Acetylization	+(C2 H2 O)				-4.61	420.22738	7.63
J17	$C_{22}H_{30}FN_3O_5$	Acetylization+Hydroxylation	+(C2 H2 O2)				-3.85	436.22255	6.83
J18	$C_{21}H_{31}N_7O_4$	N- alkyl side chain removal+Arginine binding	-(F) +(C H3 N4 O)				4.21	446.2529	8.83
J19	$C_{22}H_{30}FN_3O_6$	Dihydroxylation+Acetylization	+(C2 H2 O3)				3.32	452.22064	6.73
J20	$C_{20}H_{26}FN_3O_7S$	Ketone formation+Sulfation	-(H2) +(O4 S)				4.25	472.15683	3.37
J21	$C_{25}H_{36}FN_3O_8$	Ester hydrolysis+Glycoside	+(C5 H8 O5)				2.83	526.25741	6.89
J22	$C_{26}H_{40}FN_7O_4$	Arginine binding	+(C6 H12 N4 O)				-3.14	534.31818	8.50
J23	$C_{26}H_{39}N_3O_9$	Oxidative defluoridation+Glycoside	-(F) +(C6 H11 O6)				0.4	538.27612	4.83

J24	$C_{26}H_{38}FN_3O_8$	Glycoside	+(C6 H10 O5)				-3.21	540.26984	8.71
J25	$C_{26}H_{38}FN_7O_5$	Ketone formation+Arginine binding	+(C6 H10 N4 O2)				-2.36	548.29783	10.44

Table11 Information of MDMB-4en-PINACA and it's metabolites

Name	Formula	Transformations	Composition Change	*In vitro* metabolites			*In vivo* metabolites		
				Annot. DeltaMass [ppm]	m/z	RT [min]	Annot. DeltaMass [ppm]	m/z	RT [min]
K0	$C_{20}H_{27}N_3O_3$	MDMB-4en-PINACA		-0.45	358.21268	9.77			
K1	$C_{13}H_{12}N_2O$	Deamidation	-(C7 H15 N O2)	-0.52	213.10235	9.15			
K2	$C_{13}H_{12}N_2O_2$	Deamidation+Hydroxylation	-(C7 H15 N O)				-0.33	229.09708	4.22
K3	$C_{13}H_{14}N_2O_2$	Amide hydrolysis	-(C7 H13 N O)	-0.89	231.11301	7.51	-0.77	231.11263	4.41
K4	$C_{13}H_{12}N_2O_3$	Deamidation+Acidification	-(C7 H15 N)				-0.68	245.0919	3.58
K5	$C_{13}H_{14}N_2O_3$	Amide hydrolysis+Hydroxylation	-(C7 H13 N)	-0.37	247.10781	7.19	-1.38	247.10738	4.02
K6	$C_{19}H_{23}N_3O_3$	Ester hydrolysis+Dehydrogenation	-(C H4)	-0.79	342.18149	9.08			
K7	$C_{19}H_{25}N_3O_3$	Ester hydrolysis	-(C H2)	-0.62	344.19708	9.15			
K8	$C_{19}H_{23}N_3O_4$	Ester hydrolysis+Ketone formation	-(C H4)+（O）	-0.45	358.21268	10.97			
K9	$C_{19}H_{25}N_3O_4$	Ester hydrolysis+Hydroxylation	-(C H2)+（O）	-0.92	360.2285	10.93			
K10	$C_{20}H_{29}N_3O_4$	Hydration	+(H2 O)				-0.73	376.22281	4.98

K11	$C_{20}H_{29}N_3O_5$	Dihydrodiol	+(H2 O2)	-0.03	392.21801	7.19	-1.51	392.21741	4.25
K12	$C_{20}H_{29}N_3O_6$	Dihydrodiol+Hydroxylation	+(H2 O3)				-0.85	408.21256	4.85
K13	$C_{25}H_{37}N_5O_5$	Hydroxylation+Ornithine binding	+(C5 H10 N2 O2)				-1.01	488.28625	5.73
K14	$C_{25}H_{37}N_5O_6$	Dihydroxylation+Ornithine binding	+(C5 H10 N2 O3)				1.33	504.28233	4.79

Table12 Information of 5F-MDMB-PICA and it's metabolites

Name	Formula	Transformations	Composition Change	*In vitro* metabolites			*In vivo* metabolites		
				Annot. DeltaMass [ppm]	m/z	RT [min]	Annot. DeltaMass [ppm]	m/z	RT [min]
L0	$C_{21}H_{29}FN_2O_3$	5F-MDMB-PICA		0.64	377.22374	9.37			
L1	$C_{14}H_{15}NO_2$	Deamidation＋Oxidative defluoridation	-(C7 H14 F N O)	0.73	230.11772	7.96			
L2	$C_{14}H_{14}FNO$	Deamidation	-(C7 H15 N O2)	0.8	232.1134	8.34			
L3	$C_{14}H_{15}NO_3$	Deamidation＋Oxidative defluoridation+Hydroxylati on	-(C7 H14 F N)	0.8	246.11267	6.87			
L4	$C_{14}H_{14}FNO_2$	Deamidation+Hydroxylatio n	-(C7 H15 N O)	-0.16	248.10809	7.96			
L5	$C_{14}H_{16}FNO_2$	Amide hydrolysis	-(C7 H13 N O)	0.72	250.12396	8.34			
L6	$C_{16}H_{20}N_2O_3$	N- alkyl side chain removal	-(C5 H9 F)	0.5	289.15482	7.63			

L7	$C_{20}H_{25}FN_2O_3$	Ester hydrolysis+Dehydrogenation	-(C H4)	-4.14	361.19239	8.31			
L8	$C_{20}H_{27}FN_2O_3$	Ester hydrolysis	-(C H2)	0.39	363.20798	8.34			
L9	$C_{21}H_{30}N_2O_4$	Oxidative defluoridation	-(F) +(H O)	0.36	375.22797	7.97	-1.98	375.22709	6.73
L10	$C_{20}H_{30}N_2O_5$	Ester hydrolysis+Hydroxylation	-(C H2) +(O)	0.23	379.2027485	7.33			
L11	$C_{21}H_{28}N_2O_5$	Acidification	-(H F) +(O2)				-2.39	389.20617	8.04
L12	$C_{21}H_{30}N_2O_5$	Oxidative defluoridation+Hydroxylation	-(F) +(H O2)				-2.82	391.22165	7.59
L13	$C_{21}H_{29}FN_2O_4$	Hydroxylation	+(O)	0.49	393.21861	9.56			
L14	$C_{21}H_{29}FN_2O_4$	Hydroxylation	+(O)	0.32	393.21887	8.49			
L15	$C_{21}H_{32}N_2O_6$	Oxidative defluoridation+Dihydrodiol	-(F) +(H3 O3)				2.97	409.23453	10.22
L16	$C_{21}H_{31}FN_2O_6$	Hydroxylation+Dihydrodiol	+(H2 O3)				0.18	427.22397	8.77
L17	$C_{21}H_{31}FN_2O_7$	Dihydroxylation+Dihydrodiol	+(H2 O4)				-3.66	443.21719	5.67
L18	$C_{21}H_{33}FN_2O_7$	Dihydrodiol+Dihydrodiol	+(H4 O4)				-3.96	445.2327	5.43
L19	$C_{21}H_{28}N_2O_7S$	Oxidative defluoridation+Dehydrogenation+Sulfation	-(H F) +(O4 S)				-4.95	453.16676	8.85

L20	$C_{21}H_{27}FN_2O_6S$	Dehydrogenation+Sulfation	-(H2) +(O3 S)				-4.21	455.16275	8.83
L21	$C_{21}H_{29}FN_2O_8S$	Dihydroxylation+Sulfation	+(O5 S)				4.11	489.17215	1.45
L22	$C_{27}H_{39}FN_2O_8$	Glycoside	+(C6 H10 O5)				-0.71	539.27594	10.76
L23	$C_{27}H_{37}FN_2O_9$	Glucuronidation	+(C6 H8 O6)	0.61	553.25525	11.97			
L24	$C_{27}H_{39}FN_2O_9$	Hydroxylation+Glycoside	+(C6 H10 O6)				-4.73	555.26862	10.91
L25	$C_{27}H_{37}FN_2O_{10}$	Hydroxylation+Glucuronidation	+(C6 H8 O7)	0.83	569.25097	6.87			

Table13 Information of 5F-EMB-PICA and it's metabolites

Name	Formula	Transformations	Composition Change	*In vitro* metabolites			*In vivo* metabolites		
				Annot. DeltaMass [ppm]	m/z	RT [min]	Annot. DeltaMass [ppm]	m/z	RT [min]
M0	$C_{21}H_{29}FN_2O_3$	5F-EMB-PICA		-0.43	377.22366	9.32			
M1	$C_{14}H_{15}NO_2$	Oxidative defluoridation+Deamidation	-(C7 H14 F N O)	-0.97	230.11778	6.60			
M2	$C_{14}H_{14}FNO$	Deamidation	-(C7 H15 N O2)	2	232.11275	7.95	-1.38	232.1129	8.09
M3	$C_{14}H_{14}FNO_2$	Deamidation+Hydroxylation	-(C7 H15 N O)	-0.31	248.10821	6.74			
M3	$C_{14}H_{14}FNO_2$	Deamidation+Hydroxylation	-(C7 H15 N O)	-0.9	248.10791	6.64			
M4	$C_{14}H_{16}FNO_2$	Amide hydrolysis	-(C7 H13 N O)	-0.55	250.12392	7.96			

M5	$C_{19}H_{23}FN_2O_2$	Deester group removal	-(C2 H6 O)	-0.51	331.18146	9.32	-3.86	331.18036	9.52
M6	$C_{19}H_{26}N_2O_4$	Ester hydrolysis＋Oxidative defluoridation	-(C2 H3 F)+(O)	-0.79	347.19681	6.60			
M7	$C_{19}H_{25}FN_2O_3$	Ester hydrolysis	-(C2 H4)	-0.43	349.19235	7.96	-2.45	349.19135	8.09
M8	$C_{19}H_{25}FN_2O_4$	Ester hydrolysis+Hydroxylation	-(C H2) +(O)	-0.52	365.1873	6.74			
M9	$C_{21}H_{30}N_2O_4$	Oxidative defluoridation	-(F) +(H O)				-1.88	375.22713	6.51
M10	$C_{22}H_{32}N_6O_4$	N- alkyl side chain removal+Arginine binding	-(F) +(C H3 N4 O)				1.9	445.25662	11.24
M11	$C_{21}H_{28}N_2O_7S$	Dehydrogenation+Oxidative defluoridation+Sulfation	-(H F) +(O4 S)				-4.48	453.16697	8.97
M12	$C_{27}H_{39}FN_6O_4$	Dehydrogenation+Arginine binding	+(C6 H10 N4 O)				0.15	531.30904	6.01
M13	$C_{27}H_{42}N_6O_5$	Oxidative defluoridation+Arginine binding	-(F) +(C6 H13 N4 O2)				-5	531.32629	10.45
M14	$C_{27}H_{41}FN_6O_4$	Arginine binding	+(C6 H12 N4 O)				-0.35	533.32442	6.56
M15	$C_{27}H_{40}N_2O_9$	Oxidative defluoridation+Glycoside	-(F) +(C6 H11 O6)				1.75	537.2816	11.04
M16	$C_{27}H_{39}FN_2O_8$	Glycoside	+(C6 H10 O5)				-0.64	539.27597	10.50
M17	$C_{27}H_{37}FN_2O_9$	Glucuronidation	+(C6 H8 O6)	0.71	553.25519	11.97			

M18	$C_{27}H_{39}FN_2O_9$	Hydroxylation+Glycoside	+(C6 H10 O6)				-4.62	555.26868	10.66
M19	$C_{27}H_{41}FN_6O_6$	Dihydroxylation+Arginine binding	+(C6 H12 N4 O3)				-3.11	565.31268	8.80

Table14 Information of 5F-CYPPICA and it's metabolites

Name	Formula	Transformations	Composition Change	*In vitro* metabolites			*In vivo* metabolites		
				Annot. DeltaMass [ppm]	m/z	RT [min]	Annot. DeltaMass [ppm]	m/z	RT [min]
N0	$C_{18}H_{23}FN_2O$	5F-CYPPICA		-0.34	303.18661	8.41			
N1	$C_{13}H_{14}N_2O$	N- alkyl side chain removal	-(C5 H9 F)	0.36	215.11797	6.38			
N2	$C_{13}H_{12}N_2O_2$	N- alkyl side chain removal+Dehydrogenation+Hydroxylation	-(C5 H11 F) +(O)				-0.19	229.09711	4.20
N3	$C_{14}H_{15}NO_2$	Deamidation+Oxidative defluoridation	-(C4 H8 F N) +(O)	-0.14	230.11752	6.19			
N4	$C_{13}H_{14}N_2O_2$	N- alkyl side chain removal+Hydroxylation（Indole ring）	-(C5 H9 F) +(O)	-0.71	231.11264	4.99	-0.81	231.11262	3.88
N5	$C_{14}H_{14}FNO$	Deamidation	-(C4 H9 N)	-0.59	232.11308	6.99			
N6	$C_{14}H_{18}N_2O_2$	N- alkyl side chain removal+Dihydroxylation（Cyclobutane）	-(C4 H5 F) +(O)	-0.13	247.14407	5.56	-1.96	247.10724	3.99
N7	$C_{14}H_{14}FNO_2$	Deamidation+Hydroxylation	-(C4 H9 N) +(O)	-0.22	248.10808	6.58			

N8	$C_{14}H_{17}FN_2O$	Decyclobutane	-(C4 H6)	-0.36	249.13968	6.99			
N9	$C_{14}H_{16}FNO_2$	Amide hydrolysis	-(C4 H7 N) +(O)	-0.51	250.12366	7.62			
N10	$C_{14}H_{17}FN_2O_2$	Decyclobutane+Hydroxylation	-(C4 H6) +(O)	-0.49	265.13455	7.39			
N11	$C_{18}H_{21}FN_2O$	Dehydrogenation	-(H2)	-0.57	301.1709	7.63	1.98	301.17166	7.76
N12	$C_{18}H_{24}N_2O_2$	Oxidative defluoridation	-(F) +(H O)	-0.15	301.19101	6.93			
N13	$C_{18}H_{22}N_2O_3$	Oxidative defluoridation+Ketone formation	-(H F) +(O2)	-0.68	315.1701	6.89			
N14	$C_{18}H_{21}FN_2O_2$	Dehydrogenation+Hydroxylation（Indole ring）	-(H2) +(O)	-0.67	317.16577	8.36			
N15	$C_{18}H_{21}FN_2O_2$	Dehydrogenation+Hydroxylation（Cyclobutane）	-(H2) +(O)	-0.38	317.16586	7.24			
N16	$C_{18}H_{24}N_2O_3$	Oxidative defluoridation+ Hydroxylation（Indole ring）	-(F) +(H O2)	-0.47	317.18582	6.26			
N17	$C_{18}H_{24}N_2O_3$	Oxidative defluoridation+Hydroxylation（Cyclobutane）	-(F) +(H O2)	-0.37	317.18585	7.47			
N18	$C_{18}H_{23}FN_2O_2$	Hydroxylation（ Cyclobutane）	+(O)	-0.65	319.18143	7.85			
N19	$C_{18}H_{23}FN_2O_2$	Hydroxylation（ Indole ring）	+(O)	-0.65	319.18143	8.97			
N20	$C_{18}H_{24}N_2O_4$	Oxidative defluoridation+Dihydroxylation	-(F) +(H O3)				-1.49	333.18039	5.24

N21	$C_{18}H_{23}FN_2O_3$	Dihydroxylation（Indole ring+N-alkyl side chains）	+(O2)	-1.01	335.17621	7.06			
N22	$C_{18}H_{26}N_2O_5$	Oxidative defluoridation+Dihydrodiol+Hydroxylation	-(F) +(H3 O4)				2.84	351.19244	9.12
N23	$C_{24}H_{35}FN_6O_3$	Hydroxylation+Arginine binding	+(C6 H12 N4 O2)				-3.47	475.2811	17.01
N24	$C_{24}H_{35}FN_6O_4$	Dihydroxylation+Arginine binding	+(C6 H12 N4 O3)				-3.98	491.27571	10.64
N25	$C_{24}H_{31}FN_2O_8$	Hydroxylation（ Indole ring） +Glucuronidation	+(C6 H8 O7)	-0.63	495.21341	5.96			

Table15 Information of AMB-FUBICA and it's metabolites

Name	Formula	Transformations	Composition Change	*In vitro* metabolites			*In vivo* metabolites		
				Annot. DeltaMass [ppm]	m/z	RT [min]	Annot. DeltaMass [ppm]	m/z	RT [min]
O0	$C_{22}H_{23}FN_2O_3$	AMB-FUBICA		-0.89	383.17621	9.22			
O1	$C_{16}H_{10}FNO$	Deamidation	-(C6 H13 N O2)	-1.13	252.08163	8.31	-1.41	252.08157	8.34
O2	$C_{16}H_{10}FNO_2$	Deamidation+ Hydroxylation（ N-phenyl side chain）	-(C6 H13 N O)	-0.99	268.07657	7.15			

O3	$C_{15}H_{16}N_2O_3$	Dehydrated N-phenyl side chains+Dehydrogenation	-(C7 H7 F)	-0.06	273.12335	5.76			
O4	$C_{15}H_{18}N_2O_3$	Dehydrated N-phenyl side chains	-(C7 H5 F)	-1.15	275.1387	7.13	-0.16	275.13897	4.47
O5	$C_{15}H_{18}N_2O_4$	Dehydrated N-phenyl side chains+Hydroxylation（Indole ring）	-(C7 H5 F) +(O)	-0.39	291.13382	6.02	-1.6	291.13347	5.02
O6	$C_{15}H_{18}N_2O_4$	Dehydrated N-phenyl side chains+Hydroxylation（Tert-butyl）	-(C7 H5 F) +(O)	-0.08	291.13391	5.76			
O7	$C_{21}H_{19}FN_2O_3$	Dehydrogenation+Ester hydrolysis	-(C H4)	-0.62	367.14502	8.20			
O8	$C_{21}H_{21}FN_2O_3$	Ester hydrolysis	-(C H2)	-0.6	369.16068	8.31	-1.46	369.16036	8.34
O9	$C_{22}H_{24}N_2O_4$	Oxidative defluoridation	-(F) +(H O)				-1.04	381.18049	5.74
O10	$C_{21}H_{21}FN_2O_4$	Ester hydrolysis+Hydroxylation（N- phenyl side chain）	-(C H2) +(O)	-1.24	385.15533	7.31			
O11	$C_{21}H_{21}FN_2O_4$	Ester hydrolysis+Hydroxylation（Tert-butyl）	-(C H2) +(O)	-1.08	385.1554	7.54			
O12	$C_{20}H_{28}N_4O_4$	Dehydrated N-phenyl side chains+Ornithine binding	-(C2 F) +(H5 N2 O)				-0.86	389.218	5.29
O13	$C_{22}H_{23}FN_2O_4$	Hydroxylation	+(O)	-0.5	399.17126	8.08			
O14	$C_{20}H_{28}N_4O_5$	Dehydrated N-phenyl side chains+Hydroxylation+Ornithine binding	-(C2 F) +(H5 N2 O2)				-0.63	405.21299	5.11

O15	$C_{22}H_{21}FN_2O_5$	Dehydrogenation+Dihydroxylation（Tert-butyl）	-(H2) +(O2)	-0.96	413.15033	7.95			
O16	$C_{22}H_{23}FN_2O_5$	Dihydroxylation（N- phenyl side chain+Tert-butyl）	+(O2)	-0.8	415.16605	6.84			
O17	$C_{22}H_{25}FN_2O_6$	Dihydrodiol+Hydroxylation	+(H2 O3)				1.13	433.17743	10.38
O18	$C_{21}H_{28}N_2O_8$	Dehydrated N-phenyl side chains+Ester hydrolysis+Glucuronidation	-(C F) +(H5 O5)	3.13	437.19321	8.80	1.87	437.19266	8.85
O19	$C_{22}H_{23}FN_2O_6S$	Sulfation	+(O3 S)				-1.92	463.13247	0.91
O20	$C_{22}H_{23}FN_2O_8S$	Sulfation+Dihydroxylation	+(O5 S)				4.96	495.12564	8.80
O21	$C_{27}H_{31}FN_4O_4$	Dehydrogenation+Ornithine binding	+(C5 H8 N2 O)				-4.58	495.23795	4.82
O22	$C_{22}H_{25}FN_2O_8S$	Dihydrodiol+Sulfation	+(H2 O5 S)				1.33	497.1395	0.90
O23	$C_{28}H_{36}N_6O_5$	Oxidative defluoridation+Arginine binding	-(F) +(C6 H13 N4 O2)				-0.82	537.28156	11.34
O24	$C_{28}H_{35}FN_6O_4$	Arginine binding	+(C6 H12 N4 O)				-2.96	539.27607	10.80
O25	$C_{27}H_{29}FN_2O_9$	Ester hydrolysis+Glucuronidation	+(C5 H6 O6)	-0.99	545.19244	7.38			

Table16 Information of ADB-P7AICA and it's metabolites

Name	Formula	Transformations	Composition	*In vitro* metabolites	*In vivo* metabolites

			Change	Annot. DeltaMass [ppm]	RT [min]	Annot. DeltaMass [ppm]	m/z	RT [min]	RT [min]
P0	$C_{19}H_{28}N_4O_2$	ADB-P7AICA		-0.41	7.78	-1.77	345.22789	7.84	7.78
P1	$C_{13}H_{14}N_2O$	Deamidation	-(C6 H14 N2 O)	-0.43	7.78	-2.85	215.11728	7.82	7.78
P2	$C_{13}H_{12}N_2O_2$	N- alkyl side chain removal+Decarbonyl	-(C6 H16 N2)	-0.21	5.94	-1.19	229.09688	4.19	5.94
P3	$C_{13}H_{14}N_2O_2$	Amide hydrolysis+Dehydrogenation	-(C6 H14 N2)	-0.51	5.73	-1.97	231.11235	3.85	5.73
P4	$C_{13}H_{16}N_2O_2$	Amide hydrolysis	-(C6 H12 N2)	-0.22	7.65				7.65
P5	$C_{13}H_{14}N_2O_3$	Deamidation+Dihydroxylation	-(C6 H14 N2) +(O)			-1.86	247.10726	3.98	
P6	$C_{14}H_{18}N_4O_2$	N- alkyl side chain removal	-(C5 H10)	-0.39	4.89				4.89
P7	$C_{19}H_{25}N_3O_2$	Deamination	-(H3 N)	-0.43	7.78	-2.87	328.20101	7.82	7.78
P8	$C_{19}H_{23}N_3O_3$	Deamination+Ketone formation	-(H5 N) +(O)	-0.37	5.94	-2.7	342.1803	6.01	5.94
P9	$C_{19}H_{26}N_4O_2$	Dehydrogenation	-(H2)	-0.25	7.32	-1.59	343.21231	7.39	7.32
P10	$C_{19}H_{25}N_3O_3$	Deamination+ Hydroxylation（Tert-butyl）	-(H3 N) +(O)	-0.53	6.12				6.12
P11	$C_{19}H_{25}N_3O_3$	Deamination+ Hydroxylation（N-alkyl side chains）	-(H3 N) +(O)	-0.35	5.73	-2.13	344.19614	5.74	5.73
P12	$C_{21}H_{30}N_4O$	N- alkyl side chain removal+Dihydrodiol+Glucuronidation	-(O) +(C2 H2)	-4.91	6.93				6.93
P13	$C_{19}H_{26}N_4O_3$	Ketone formation	-(H2) +(O)	-0.86	6.29	-2.57	359.20685	6.33	6.29
P14	$C_{19}H_{26}N_4O_3$	Hydroxylation（N- Indole ring）+Dehydrogenation	-(H2) +(O)	-0.61	5.94	-2.79	359.20677	5.98	5.94

P15	$C_{19}H_{26}N_4O_3$	Hydroxylation（N-alkyl side chains）+Dehydrogenation	-(H2) +(O)	-0.61	6.83				6.83
P16	$C_{19}H_{25}N_3O_4$	Deamination+Dihydroxylation	-(H3 N) +(O2)			2.01	360.1925	4.46	
P17	$C_{19}H_{28}N_4O_3$	Hydroxylation（N-alkyl side chains）	+(O)	-1.01	6.74	-2.96	361.22235	6.86	6.74
P18	$C_{19}H_{28}N_4O_3$	Hydroxylation（Tert-butyl）	+(O)	-0.59	5.73	-2.57	361.22249	5.76	5.73
P19	$C_{19}H_{28}N_4O_3$	Hydroxylation（N- Indole ring）	+(O)	-0.59	6.25	-2.31	361.22259	6.15	6.25
P20	$C_{19}H_{26}N_4O_4$	Acidification	-(H2) +(O2)			-1.51	375.20212	5.22	
P21	$C_{19}H_{26}N_4O_4$	Ketone formation+Hydroxylation	-(H2) +(O2)			-1.24	375.20222	4.68	
P22	$C_{19}H_{25}N_3O_5$	Hydrolysis+Acidification	-(H3 N) +(O3)			-1.76	376.18604	4.92	
P23	$C_{19}H_{28}N_4O_4$	Dihydroxylation（N- Indole ring+N-alkyl side chains）	+(O2)	-0.67	5.23	-1.73	377.21768	5.26	5.23
P24	$C_{19}H_{28}N_4O_4$	Dihydroxylation（N-alkyl side chains）	+(O2)	-0.51	4.90				4.90
P25	$C_{19}H_{27}N_3O_5$	Hydrolysis+Dihydroxylation	-(H N) +(O3)			-2.25	378.2015	3.69	
P26	$C_{19}H_{29}N_3O_5$	Hydrolysis+Dihydrodiol	-(N) +(H O3)			-2.43	380.21708	5.04	
P27	$C_{19}H_{28}N_4O_5$	Trihydroxylation	+(O3)			-2.95	393.21209	4.29	
P28	$C_{19}H_{27}N_3O_6$	Trihydroxylation+Hydrolysis	-(H N) +(O4)			-1.54	394.19666	5.00	
P29	$C_{19}H_{23}N_3O_5S$	Deamination+Dehydrogenation+Sulfation	-(H5 N) +(O3 S)			3.71	406.14462	0.96	
P30	$C_{21}H_{30}N_4O_5$	Dihydroxylation+Acetylization	+(C2 H2 O3)			-2.16	419.22799	5.50	
P31	$C_{19}H_{27}N_3O_6S$	Hydrolysis+Sulfation	-(H N) +(O4 S)			-2.13	426.16843	5.24	

P32	$C_{24}H_{36}N_6O_3$	Dehydrogenation+Ornithine binding	+(C5 H8 N2 O)			-1.83	457.29133	10.93	
P33	$C_{19}H_{27}N_3O_8S$	Hydrolysis+Dihydroxylation+Sulfation	-(H N) +(O6 S)			-1.63	458.15842	3.44	
P34	$C_{24}H_{38}N_6O_3$	Ornithine binding	+(C5 H10 N2 O)			2.45	459.30894	7.98	
P35	$C_{24}H_{36}N_6O_4$	Glutamine binding	+(C5 H8 N2 O2)			-1.49	473.28638	8.99	
P36	$C_{24}H_{38}N_6O_4$	Hydroxylation+Ornithine binding	+(C5 H10 N2 O2)			3.42	475.30435	6.62	
P37	$C_{24}H_{36}N_6O_5$	Dihydroxylation+Glutamine binding	+(C5 H8 N2 O3)			-0.5	489.28175	10.72	
P38	$C_{24}H_{38}N_6O_5$	Dihydroxylation+Ornithine binding	+(C5 H10 N2 O3)			1.04	491.29816	15.20	
P39	$C_{25}H_{38}N_4O_7$	Glycoside	+(C6 H10 O5)			-2.38	507.28012	5.55	
P40	$C_{25}H_{40}N_8O_4$	Hydroxylation+Arginine binding	+(C6 H12 N4 O2)			3.71	517.32644	6.62	

Table17 Information of JWH-019 and it's metabolites

Name	Formula	Transformations	Composition Change	*In vitro* metabolites			*In vivo* metabolites		
				Annot. DeltaMass [ppm]	m/z	RT [min]	Annot. DeltaMass [ppm]	m/z	RT [min]
Q0	$C_{25}H_{25}NO$	JWH-019		-1.01	356.20053	11.68	-1.54	356.20034	11.94
Q1	$C_{19}H_{13}NO$	N- alkyl side chain removal	-(C6 H12)	-0.89	272.10675	8.65			

Q2	$C_{19}H_{15}NO_3$	N- alkyl side chain removal+Dihydrodiol	-(C6 H10) +(O2)	-0.44	306.11234	6.08			
Q3	$C_{25}H_{23}NO$	Dehydrogenation	-(H2)	-1.03	354.18488	11.12			
Q4	$C_{25}H_{23}NO_2$	Ketone formation	-(H2) +(O)	-0.77	370.17987	9.82			
Q5	$C_{25}H_{25}NO_2$	Hydroxylation（Naphthyl）	+(O)	-1	372.19543	8.32			
Q6	$C_{25}H_{25}NO_2$	Hydroxylation（N-alkyl side chains）	+(O)	-0.67	372.19556	10.23			
Q7	$C_{25}H_{25}NO_2$	Hydroxylation（Indole ring）	+(O)	-0.34	372.19568	9.16			
Q8	$C_{25}H_{23}NO_3$	Acidification	-(H2) +(O2)	-1.4	386.17453	9.00			
Q9	$C_{25}H_{25}NO_3$	Dihydroxylation（Naphthyl+N-alkyl side chains）	+(O2)	-1.7	388.19006	8.01			
Q10	$C_{25}H_{25}NO_3$	Dihydroxylation（N-alkyl side chains）	+(O2)	-1.22	388.19025	10.82			
Q11	$C_{25}H_{25}NO_3$	Dihydroxylation（Naphthyl）	+(O2)	-1.07	388.19031	7.88			
Q12	$C_{25}H_{27}NO_3$	Dihydrodiol	+(H2 O2)	-1.12	390.20593	9.46			
Q13	$C_{25}H_{25}NO_4$	Ketone formation+Dihydrodiol	+(O3)	-1.35	404.18509	7.38			
Q14	$C_{25}H_{27}NO_4$	Dihydrodiol+Hydroxylation（N-alkyl side chains）	+(H2 O3)	-0.88	406.20093	6.89			
Q15	$C_{25}H_{27}NO_4$	Dihydrodiol+Hydroxylation（Naphthyl）	+(H2 O3)	-0.5	406.20108	9.14			
Q16	$C_{25}H_{27}NO_5$	Dihydrodiol+Dihydroxylation（Naphthyl+N-alkyl side chains）	+(H2 O4)	-0.87	422.19583	6.03			

Q17	$C_{27}H_{30}N_2O_4$	Dihydrodiol+Glycine binding	+(C2 H5 N O3)				-2.71	447.22662	3.92
Q18	$C_{31}H_{35}N_5O_2$	Dehydrogenation+Arginine binding	+(C6 H10 N4 O)				2.09	510.28741	7.01
Q19	$C_{31}H_{37}N_5O_2$	Arginine binding	+(C6 H12 N4 O)				2.51	512.30328	6.00
Q20	$C_{31}H_{37}N_5O_2$	Arginine binding	+(C6 H12 N4 O)				3.34	512.30371	6.95
Q21	$C_{31}H_{37}N_5O_3$	Hydroxylation+Arginine binding	+(C6 H12 N4 O2)				2.94	528.29846	5.56
Q22	$C_{31}H_{33}NO_8$	Glucuronidation+Hydroxylation（N-alkyl side chains）	+(C6 H8 O7)	-0.87	548.22742	8.85			
Q23	$C_{31}H_{33}NO_8$	Glucuronidation+Hydroxylation（Naphthyl）	+(C6 H8 O7)	-0.76	548.22748	8.42			
Q24	$C_{31}H_{33}NO_9$	Glucuronidation+Dihydroxylation（Naphthyl+N-alkyl side chains）	+(C6 H8 O8)	-1.03	564.22223	8.38			
Q25	$C_{31}H_{33}NO_9$	Glucuronidation+Dihydroxylation（Naphthyl）	+(C6 H8 O8)	-0.59	564.22247	9.05			
Q26	$C_{31}H_{35}NO_9$	Glucuronidation+Dihydrodiol	+(C6 H10 O8)	-0.85	566.23798	8.42			
Q27	$C_{31}H_{35}NO_{10}$	Glucuronidation+Dihydrodiol+Hydroxylation（Naphthyl）	+(C6 H10 O9)	-0.9	582.23285	6.60			

Table18 Information of JWH-200 and it's metabolites

Name	Formula	Transformations	Composition Change	*In vitro* metabolites			*In vivo* metabolites		
				Annot. DeltaMass [ppm]	m/z	RT [min]	Annot. DeltaMass [ppm]	m/z	RT [min]
R0	$C_{25}H_{24}N_2O_2$	JWH-200		-0.36	385.19092	6.66			
R1	$C_{19}H_{13}NO$	N- alkyl side chain removal	-(C6 H11 N O)	-0.89	272.10675	8.62			
R2	$C_{21}H_{17}NO$	Diethylmorpholine	-(C4 H7 N O)	-0.77	300.13806	7.92			
R3	$C_{21}H_{17}NO_2$	Diethylmorpholine+Oxidation	-(C4 H7 N)	-0.47	316.13306	8.19			
R4	$C_{21}H_{15}NO_3$	Diethylmorpholine+Oxidation+Ketone formation	-(C4 H9 N) +(O)	-0.87	330.11218	8.12			
R5	$C_{21}H_{17}NO_3$	Diethylmorpholine+Oxidation+Hydroxylation（Indole ring）	-(C4 H7 N) +(O)	-0.67	332.1279	7.14			
R6	$C_{21}H_{17}NO_3$	Diethylmorpholine+Oxidation+Hydroxylation（Naphthyl）	-(C4 H7 N) +(O)	-0.48	332.12796	6.90			
R7	$C_{25}H_{22}N_2O_2$	Dehydrogenation	-(H2)	-1.01	383.17502	6.61			
R8	$C_{25}H_{24}N_2O_3$	Hydroxylation（N-alkyl side chains）	+(O)	-0.9	401.18561	5.72			
R9	$C_{25}H_{24}N_2O_3$	Hydroxylation（ Naphthyl）	+(O)	-0.52	401.18576	6.09			
R10	$C_{25}H_{26}N_2O_4$	Dihydrodiol	+(H2 O2)	-0.44	419.19635	5.24			
R11	$C_{25}H_{24}N_2O_5$	Trihydroxylation	+(O3)				2.76	433.17699	10.31
R12	$C_{25}H_{22}N_2O_5S$	Dehydrogenation+Sulfation	-(H2) +(O3 S)				-1.35	463.13159	1.07

R13	$C_{25}H_{24}N_2O_7S$	Dihydroxylation+Sulfation	+(O5 S)				3.21	497.13929	0.91
R14	$C_{31}H_{34}N_6O_3$	Dehydrogenation+Arginine binding	+(C6 H10 N4 O)				-0.81	539.27608	10.75
R15	$C_{31}H_{36}N_6O_4$	Hydroxylation+Arginine binding	+(C6 H12 N4 O2)				-1.16	557.28643	10.49
R16	$C_{31}H_{34}N_2O_{10}$	Glucuronidation+Dihydrodiol	+(C6 H10 O8)	-0.18	595.22852	4.95	-3.36	595.22662	0.93

Table19 Information of BIM-2201 and it's metabolites

Name	Formula	Transformations	Composition Change	*In vitro* metabolites			*In vivo* metabolites		
				Annot. DeltaMass [ppm]	m/z	RT [min]	Annot. DeltaMass [ppm]	m/z	RT [min]
S0	$C_{23}H_{21}FN_2O$	FUBIMINA		-0.89	361.17075	10.24	1.56	361.17163	10.00
S1	$C_{18}H_{12}N_2O$	N- alkyl side chain removal	-(C5 H9 F)	-1.03	273.10196	8.36			
S2	$C_{18}H_{12}N_2O_2$	N- alkyl side chain removal+Hydroxylation(Benzimidazole ring)	-(C5 H9 F) +(O)	-0.69	289.09695	7.14			
S3	$C_{23}H_{22}N_2O$	Defluorination	-(F) +(H)	-0.29	343.18039	5.66			
S4	$C_{23}H_{22}N_2O_2$	Oxidative defluoridation	-(F) +(H O)	-0.99	359.17505	8.59			
S5	$C_{23}H_{22}N_2O_3$	Oxidative defluoridation+Hydroxylation （Naphthyl）	-(F) +(H O2)	-1.14	375.16989	7.60			
S6	$C_{23}H_{22}N_2O_3$	Oxidative defluoridation+Hydroxylation(Benzimidazole ring)	-(F) +(H O2)	-0.33	375.1702	5.68			

S7	$C_{23}H_{21}FN_2O_2$	Hydroxylation(Benzimidazole ring)	+(O)	-1.53	377.16541	8.60			
S8	$C_{23}H_{21}FN_2O_2$	Hydroxylation(N-alkyl side chains)	+(O)	-0.72	377.16571	7.67			
S9	$C_{23}H_{19}FN_2O_3$	Ketone formation+Hydroxylation（Naphthyl）	-(H2) +(O2)	-0.9	391.1449	6.50			
S10	$C_{23}H_{22}N_2O_4$	Oxidative defluoridation+Dihydroxylation(Benzimidazole ring+Naphthyl)	-(F) +(H O3)	-0.89	391.16489	6.46			
S11	$C_{23}H_{21}FN_2O_3$	Dihydroxylation（Naphthyl+N-alkyl side chains）	+(O2)	-1.66	393.16025	7.51			
S12	$C_{23}H_{21}FN_2O_3$	Dihydroxylation（Benzimidazole ring）	+(O2)	-1.19	393.16043	5.49			
S13	$C_{23}H_{24}N_2O_4$	Oxidative defluoridation+Dihydrodiol（Naphthyl）	-(F) +(H3 O3)	-1.49	393.1803	6.17			
S14	$C_{23}H_{23}FN_2O_3$	Dihydrodiol（Benzimidazole ring）	+(H2 O2)	-0.86	395.17621	7.59			
S15	$C_{23}H_{23}FN_2O_3$	Dihydrodiol（Naphthyl）	+(H2 O2)	-0.47	395.17636	5.62			
S16	$C_{23}H_{23}FN_2O_4$	Hydroxylation（Naphthyl）+Dihydrodiol(Benzimidazole ring)	+(H2 O3)	-1.3	411.17093	7.50			
S17	$C_{23}H_{23}FN_2O_4$	Hydroxylation+Dihydrodiol（Naphthyl）	+(H2 O3)	-0.56	411.17123	6.72			

S18	$C_{25}H_{24}FN_3O_2$	Glycine binding	+(C2 H3 N O)				0.2	418.19262	3.47
S19	$C_{25}H_{22}FN_3O_3$	Ketone formation+Glycine binding	+(C2 H N O2)				-3.56	432.17026	0.87
S20	$C_{25}H_{24}FN_3O_3$	Hydroxylation+Glycine binding	+(C2 H3 N O2)				2.48	434.18852	1.34
S21	$C_{28}H_{31}FN_4O_2$	Ornithine binding	+(C5 H10 N2 O)				0.16	475.25046	3.29
S22	$C_{29}H_{34}N_6O_3$	Oxidative defluoridation+Arginine binding	-(F) +(C6 H13 N4 O2)				-0.42	515.2763	10.99
S23	$C_{29}H_{34}N_6O_4$	Hydroxylation+Oxidative defluoridation+Arginine binding	-(F) +(C6 H13 N4 O3)				-4.96	531.2688	11.09
S24	$C_{29}H_{30}N_2O_8$	Oxidative defluoridation+Glucuronidation	-(F) +(C6 H9 O7)	-0.52	535.20721	7.27			
S25	$C_{29}H_{30}N_2O_9$	Oxidative defluoridation+Hydroxylation（Naphthyl）＋Glucuronidation	-(F) +(C6 H9 O8)	-0.8	551.20197	6.19			
S26	$C_{29}H_{30}N_2O_9$	Oxidative defluoridation+Hydroxylation(Benzimidazole ring)+Glucuronidation	-(F) +(C6 H9 O8)	-0.36	551.20221	6.69			
S27	$C_{29}H_{29}FN_2O_8$	Hydroxylation(Benzimidazole ring)+Glucuronidation	+(C6 H8 O7)	-1.02	553.19751	7.66			
S28	$C_{29}H_{29}FN_2O_8$	Hydroxylation（Naphthyl）+Glucuronidation	+(C6 H8 O7)	-0.79	553.19763	7.27			

S29	$C_{29}H_{29}FN_2O_9$	Dihydroxylation（Naphthyl+N-alkyl side chains）+Glucuronidation	+(C6 H8 O8)	-1.27	569.19226	7.49			

Table20 Information of BIM-018 and it's metabolites

Name	Formula	Transformations	Composition Change	*In vitro* metabolites			*In vivo* metabolites		
				Annot. DeltaMass [ppm]	m/z	RT [min]	Annot. DeltaMass [ppm]	m/z	RT [min]
T0	$C_{23}H_{22}N_2O$	BIM-018		-0.2	343.18042	11.51	-1.63	343.17993	11.33
T1	$C_{18}H_{12}N_2O_2$	N- alkyl side chain removal+Hydroxylation(Benzimidazole ring)	-(C5 H10) +(O)	-1.01	289.09686	7.34			
T2	$C_{23}H_{20}N_2O$	Dehydrogenation	-(H2)	-0.04	341.16483	10.99			
T3	$C_{23}H_{20}N_2O_2$	Ketone formation	-(H2) +(O)	-0.93	357.15942	9.37			
T4	$C_{23}H_{22}N_2O_2$	Hydroxylation(Benzimidazole ring)	+(O)	-1.08	359.17502	9.72			
T5	$C_{23}H_{22}N_2O_2$	Hydroxylation（Naphthyl）	+(O)	-0.23	359.17532	6.29			
T6	$C_{23}H_{20}N_2O_3$	Ketone formation+Hydroxylation(Benzimidazole ring)	-(H2) +(O2)	-1	373.1543	7.31			
T7	$C_{23}H_{20}N_2O_3$	Ketone formation+Hydroxylation（Naphthyl）	-(H2) +(O2)	-1	373.1543	8.22			
T8	$C_{23}H_{22}N_2O_3$	Dihydroxylation(Benzimidazole ring+N-alkyl side chains)	+(O2)	-1.06	375.16992	8.89			

T9	$C_{23}H_{22}N_2O_3$	Dihydroxylation（Naphthyl+N-alkyl side chains）	+(O2)	-0.25	375.17023	5.51			
T10	$C_{23}H_{24}N_2O_3$	Dihydrodiol	+(H2 O2)	-0.96	377.18561	9.01			
T11	$C_{23}H_{22}N_2O_4$	Trihydroxylation（Naphthyl+N-alkyl side chains×2）	+(O3)	-0.89	391.16489	6.73			
T12	$C_{23}H_{24}N_2O_4$	Hydroxylation+Dihydrodiol（Naphthyl）	+(H2 O3)	-1.57	393.18027	7.49			
T13	$C_{23}H_{24}N_2O_4$	Hydroxylation（Naphthyl）+Dihydrodiol(Benzimidazole ring)	+(H2 O3)	-1.1	393.18045	8.56			
T14	$C_{25}H_{26}N_2O_4$	Dihydrodiol+Acetylization	+(C2 H4 O3)				-3.07	419.19525	5.18
T15	$C_{25}H_{24}N_2O_5$	Hydroxylation+Dihydrodiol+Acetylization	+(C2 H2 O4)				2.5	433.17688	10.25
T16	$C_{28}H_{36}N_4O_4$	Dihydrodiol+Ornithine binding	+(C5 H14 N2 O3)				-4.63	493.27865	7.60
T17	$C_{29}H_{32}N_6O_2$	Dehydrogenation+Arginine binding	+(C6 H10 N4 O)				-0.86	497.26552	11.77
T18	$C_{28}H_{36}N_4O_5$	Hydroxylation+Dihydrodiol+Ornithine binding	+(C5 H14 N2 O4)				-2.08	509.27479	7.87
T19	$C_{29}H_{34}N_6O_3$	Hydroxylation+Arginine binding	+(C6 H12 N4 O2)				-0.81	515.2761	10.91
T20	$C_{28}H_{34}N_4O_6$	Dihydroxylation+Dihydrodiol+Ornithine binding	+(C5 H12 N2 O5)				0.95	523.25561	7.64

T21	$C_{29}H_{34}N_6O_4$	Dihydroxylation+Arginine binding	+(C6 H12 N4 O3)				-4.9	531.26883	11.02
T22	$C_{29}H_{36}N_6O_4$	Dihydrodiol+Arginine binding	+(C6 H14 N4 O3)				-4.76	533.28455	16.97
T23	$C_{29}H_{30}N_2O_8$	Hydroxylation(Benzimidazole ring)+Glucuronidation	+(C6 H8 O7)	-0.98	535.20697	8.50			
T24	$C_{29}H_{30}N_2O_8$	Hydroxylation（Naphthyl）+Glucuronidation	+(C6 H8 O7)	-0.63	535.20715	7.60			
T25	$C_{29}H_{30}N_2O_9$	Dihydroxylation+Glucuronidation（Naphthyl）	+(C6 H8 O8)	-0.58	551.20209	8.38			
T26	$C_{29}H_{30}N_2O_9$	Dihydroxylation+Glucuronidation(Benzimidazole ring+Naphthyl)	+(C6 H8 O8)	-0.03	551.20239	6.39			
T27	$C_{29}H_{32}N_2O_{10}$	Hydroxylation（N-alkyl side chains）+Dihydrodiol（Naphthyl）＋Glucuronidation	+(C6 H10 O9)	0.07	569.21301	4.96			

Table21 Information ofUR-144 and it's metabolites

Name	Formula	Transformations	Composition Change	*In vitro* metabolites			*In vivo* metabolites		
				Annot. DeltaMass [ppm]	m/z	RT [min]	Annot. DeltaMass [ppm]	m/z	RT [min]
U0	$C_{21}H_{29}NO$	UR-144		-0.63	312.23199	12.49	-2.04	312.23156	11.81
U1	$C_{16}H_{19}NO$	N- alkyl side chain removal	-(C5 H10)	-0.61	242.15379	5.72			

U2	$C_{16}H_{19}NO_2$	N- alkyl side chain removal+Hydroxylation （Cyclopropyl）	-(C5 H10) +(O)	-0.68	258.14868	8.74			
U3	$C_{21}H_{27}NO$	Dehydrogenation	-(H2)	-0.75	310.21631	8.46			
U4	$C_{21}H_{25}NO_2$	Dehydrogenation+Ketone formation	-(H4) +(O)	-0.3	324.19571	10.62			
U5	$C_{21}H_{27}NO_2$	Ketone formation	-(H2) +(O)	-0.65	326.21124	11.47	-2.16	326.21075	10.31
U6	$C_{21}H_{27}NO_2$	Dehydrogenation+Hydroxylation （N-alkyl side chains）	-(H2) +(O)	-0.37	326.21133	6.78			
U7	$C_{21}H_{27}NO_2$	Dehydrogenation+Hydroxylation （Indole ring）	-(H2) +(O)	-0.19	326.2114	10.64			
U8	$C_{21}H_{29}NO_2$	Hydroxylation（ Cyclopropyl）	+(O)	-0.45	328.22696	11.00	-2.31	328.22635	7.29
U9	$C_{21}H_{29}NO_2$	Hydroxylation（ Indole ring）	+(O)	-0.26	328.22702	10.18			
U10	$C_{21}H_{27}NO_3$	Acidification	-(H2) +(O2)	-0.75	342.20612	8.71	-1.77	342.20577	9.92
U11	$C_{21}H_{27}NO_3$	Ketone formation+Hydroxylation （N-alkyl side chains）	-(H2) +(O2)	-0.48	342.20621	8.34	-0.14	342.20632	10.13
U12	$C_{21}H_{27}NO_3$	Dehydrogenation+Dihydroxylation	-(H2) +(O2)	-0.48	342.20621	8.54			
U13	$C_{21}H_{27}NO_3$	Ketone formation+Hydroxylation （Cyclopropyl）	-(H2) +(O2)	-0.21	342.2063	11.52			
U14	$C_{21}H_{29}NO_3$	Dihydroxylation（ N - alkyl side chains+ Cyclopropyl）	+(O2)	-2.5	344.22116	9.24			

U15	$C_{21}H_{29}NO_3$	Dihydroxylation（Indole ring+ Cyclopropyl）	+(O2)	-1.26	344.22159	8.51			
U16	$C_{23}H_{31}NO_3$	Acetylization	+(C2 H2 O2)				-3.67	370.23632	7.05
U17	$C_{26}H_{39}N_3O_2$	Ornithine binding	+(C5 H10 N2 O)				0.79	426.31184	11.99
U18	$C_{26}H_{39}N_3O_2$	Ornithine binding	+(C5 H10 N2 O)				0.86	426.31187	12.04
U19	$C_{26}H_{37}N_3O_3$	Ornithine binding+Ketone formation	+(C5 H8 N2 O2)				1.17	440.29128	11.75
U20	$C_{26}H_{39}N_3O_3$	Ornithine binding+Hydroxylation	+(C5 H10 N2 O2)				0.59	442.30668	11.85
U21	$C_{27}H_{39}N_5O_2$	Dehydrogenation+Arginine binding	+(C6 H10 N4 O)				-3.11	466.3162	7.22
U22	$C_{27}H_{39}N_5O_2$	Arginine binding+Dehydrogenation	+(C6 H10 N4 O)				-2.35	466.31656	9.24
U23	$C_{27}H_{37}NO_8$	Hydroxylation（ Cyclopropyl）+Glucuronidation	+(C6 H8 O7)	-0.93	504.25873	9.00			
U24	$C_{27}H_{37}NO_8$	Hydroxylation（ Indole ring）+Glucuronidation	+(C6 H8 O7)	-0.68	504.25885	9.23			

Table22 Information of AB-005 and it's metabolites

Name	Formula	Transformations	Composition	*In vitro* metabolites	*In vivo* metabolites

			Change	Annot. DeltaMass [ppm]	m/z	RT [min]	Annot. DeltaMass [ppm]	m/z	RT [min]
V0	$C_{23}H_{32}N_2O$	AB-005		-0.21	353.25867	7.30	-0.58	353.25854	7.60
V1	$C_{16}H_{19}NO$	N- alkyl side chain removal	-(C7 H13 N)	-0.36	242.15385	5.05			
V2	$C_{22}H_{30}N_2O$	Demethylation	-(C H2)	0.22	339.24316	7.18			
V3	$C_{23}H_{30}N_2O$	Dehydrogenation	-(H2)	-0.14	351.24304	6.96			
V4	$C_{22}H_{28}N_2O_2$	Demethylation+Dehydrogenation+ Hydroxylation（Cyclopropyl）	-(C H4) +(O)	-0.88	353.22205	6.06			
V5	$C_{22}H_{30}N_2O_2$	Demethylation+Hydroxylation （Cyclopropyl）	-(C H2) +(O)	-0.51	355.23782	5.58			
V6	$C_{23}H_{28}N_2O_2$	Dehydrogenation+Ketone formation	-(H4) +(O)	0.07	365.22238	6.96			
V7	$C_{23}H_{30}N_2O_2$	Dehydrogenation+Hydroxylation （Cyclopropyl）	-(H2) +(O)	-0.83	367.2377	6.60			
V8	$C_{23}H_{32}N_2O_2$	Hydroxylation（Cyclopropyl）	+(O)	-0.81	369.25336	6.34	-1.98	369.25293	5.85
V9	$C_{22}H_{30}N_2O_3$	Demethylation+Dihydroxylation	-(C H2) +(O2)	-0.19	371.23285	5.43			
V10	$C_{23}H_{30}N_2O_3$	Dehydrogenation+Dihydroxylatio n	-(H2) +(O2)	-0.9	383.23257	6.25	-2.11	383.23211	6.31
V11	$C_{23}H_{32}N_2O_3$	Dihydroxylation	+(O2)	-0.8	385.24826	4.73			
V12	$C_{23}H_{34}N_2O_5$	Dihydroxylation+Dihydrodiol	+(H2 O4)				3.02	419.25531	10.72
V13	$C_{28}H_{42}N_4O_3$	Hydroxylation+Ornithine binding	+(C5 H10 N2 O2)				-4.98	483.33057	7.44

V14	$C_{29}H_{42}N_2O_6$	Glycoside	+(C6 H10 O5)				1.58	515.31238	12.30
V15	$C_{29}H_{44}N_6O_3$	Hydroxylation+Arginine binding	+(C6 H12 N4 O2)				-4.45	525.35243	6.58
V16	$C_{29}H_{42}N_2O_7$	Hydroxylation+Glycoside	+(C6 H10 O6)				4.1	531.30865	6.48
V17	$C_{29}H_{40}N_2O_9$	Dihydroxylation+Glucuronidation	+(C6 H8 O8)				-0.53	561.28036	10.01

Table23 Information ofFUB-144 and it's metabolites

Name	Formula	Transformations	Composition Change	*In vitro* metabolites			*In vivo* metabolites		
				Annot. DeltaMass [ppm]	m/z	RT [min]	Annot. DeltaMass [ppm]	m/z	RT [min]
W0	$C_{23}H_{24}FNO$	FUB-144		-0.53	350.19128	11.06			
W1	$C_{16}H_{19}NO$	Dehydrated N-phenyl side chains	-(C7 H5 F)	-0.42	242.15384	5.02			
W2	$C_{16}H_{19}NO_2$	Dehydrated N-phenyl side chains+Hydroxylation （Cyclopropyl）	-(C7 H5 F) +(O)	-0.56	258.14871	8.06			
W3	$C_{23}H_{22}FNO$	Dehydrogenation	-(H2)	-1.16	348.17542	10.60	1.12	348.17621	3.51
W4	$C_{23}H_{20}FNO_2$	Dehydrogenation+Ketone formation	-(H4) +(O)	-1.59	362.15451	9.20			
W5	$C_{23}H_{22}FNO_2$	Ketone formation	-(H2) +(O)	-1.14	364.17032	9.77			
W6	$C_{23}H_{24}FNO_2$	Hydroxylation（ Cyclopropyl）	+(O)	-1.21	366.18594	9.64			
W7	$C_{23}H_{24}FNO_2$	Hydroxylation（N- phenyl side chain）	+(O)	-1.04	366.186	9.99			

W8	$C_{23}H_{24}FNO_2$	Hydroxylation（Indole ring）	+(O)	-0.79	366.1861	9.05	0.47	366.18655	9.11
W9	$C_{23}H_{22}FNO_3$	Ketone formation+Hydroxylation	-(H2) +(O2)	-0.88	380.16531	10.13	-1.2	380.16519	9.37
W10	$C_{23}H_{24}FNO_3$	Dihydroxylation（N- phenyl side chain+ Cyclopropyl）	+(O2)	-1.42	382.18076	7.60			
W11	$C_{23}H_{24}FNO_3$	Dihydroxylation（Cyclopropyl）	+(O2)	-1.26	382.18082	8.06			
W12	$C_{25}H_{24}FNO_2$	Dehydrogenation+Acetylization	+(C2 O)				0.99	390.18677	3.18
W13	$C_{22}H_{29}NO_6$	Dehydrated N-phenyl side chains+Glycoside	-(C F) +(H5 O5)				-2.29	404.20584	8.66
W14	$C_{29}H_{36}FN_5O_2$	Arginine binding	+(C6 H12 N4 O)				-3.16	506.29099	8.50
W15	$C_{29}H_{36}FN_5O_3$	Arginine binding+Hydroxylation	+(C6 H12 N4 O2)				-4.78	522.285	7.29
W16	$C_{29}H_{36}FN_5O_4$	Arginine binding+Dihydroxylation	+(C6 H12 N4 O3)				-4.46	538.28001	6.58
W17	$C_{29}H_{32}FNO_8$	Glucuronidation+Hydroxylation	+(C6 H8 O7)	-0.61	542.21814	7.80			
W18	$C_{29}H_{32}FNO_9$	Dihydroxylation+Glucuronidation	+(C6 H8 O8)				-1.62	558.21249	17.06

Table24 Information of JWH-030 and it's metabolites

Name	Formula	Transformations	Composition Change	*In vitro* metabolites			*In vivo* metabolites		
				Annot. DeltaMass [ppm]	m/z	RT [min]	Annot. DeltaMass [ppm]	m/z	RT [min]

X0	$C_{20}H_{21}NO$	JWH-030		-0.64	292.1694	7.62			
X1	$C_{20}H_{19}NO$	Dehydrogenation	-(H2)	-0.35	290.15384	9.86			
X2	$C_{20}H_{19}NO_2$	Dehydrogenation+Hydroxylation(N-alkyl side chains)	-(H2) +(O)	-0.57	306.14868	8.21			
X3	$C_{20}H_{21}NO_2$	Hydroxylation(N-alkyl side chains)	+(O)	-1.04	308.16418	7.44	1.87	308.16508	6.86
X4	$C_{20}H_{19}NO_3$	Ketone formation+Hydroxylation(Naphthyl)	-(H2) +(O2)	-0.86	322.14349	7.25			
X5	$C_{20}H_{21}NO_3$	Dihydroxylation(Naphthyl+N-alkyl side chains)	+(O2)	-0.65	324.15921	7.12			
X6	$C_{20}H_{23}NO_3$	Dihydrodiol	+(H2 O2)	-0.82	326.1748	7.79			
X7	$C_{22}H_{23}NO_2$	Acetylization	+(C2 H2 O)				-3.69	334.17892	4.37
X8	$C_{20}H_{23}NO_4$	Hydroxylation(Naphthyl)+Dihydrodiol	+(H2 O3)	-0.81	342.16971	7.66			
X9	$C_{22}H_{23}NO_3$	Hydroxylation+Acetylization	+(C2 H2 O2)				-3.41	350.17388	3.56
X10	$C_{20}H_{23}NO_6S$	Dihydrodiol+Sulfation	+(H2 O5 S)				-0.91	406.13152	0.88
X11	$C_{25}H_{29}N_3O_3$	Glutamine binding	+(C5 H8 N2 O2)				-2.77	420.22701	7.63
X12	$C_{25}H_{31}N_3O_3$	Hydroxylation+Ornithine binding	+(C5 H10 N2 O2)				-2.78	422.24265	7.63
X13	$C_{25}H_{29}N_3O_4$	Hydroxylation+Glutamine binding	+(C5 H8 N2 O3)				-0.75	436.22276	4.80
X14	$C_{25}H_{31}N_3O_4$	Dihydroxylation+Ornithine	+(C5 H10 N2				-1.06	438.23827	4.72

		binding	O3)						
X15	$C_{26}H_{31}N_5O_2$	Dehydrogenation+Arginine binding	+(C6 H10 N4 O)				-4.95	446.25285	8.88
X16	$C_{25}H_{31}N_3O_5$	Dihydrodiol+Glutamine binding	+(C5 H10 N2 O4)				-0.62	454.23337	4.46
X17	$C_{26}H_{33}N_5O_3$	Hydroxylation+Arginine binding	+(C6 H12 N4 O2)				-3.55	464.26397	6.55
X18	$C_{26}H_{29}NO_8$	Hydroxylation(N-alkyl side chains)+Glucuronidation	+(C6 H8 O7)	-0.5	484.19635	6.80			
X19	$C_{26}H_{29}NO_9$	Dihydroxylation+Glucuronidation (Naphthyl+N-alkyl side chains)	+(C6 H8 O8)	-0.75	500.19113	7.48			
X20	$C_{26}H_{31}NO_9$	Dihydrodiol+Glucuronidation	+(C6 H10 O8)	-1.84	502.20624	6.62			

Table25 Information of JWH-307 and it's metabolites

Name	Formula	Transformations	Composition Change	*In vitro* metabolites			*In vivo* metabolites		
				Annot. DeltaMass [ppm]	m/z	RT [min]	Annot. DeltaMass [ppm]	m/z	RT [min]
Y0	$C_{26}H_{24}FNO$	JWH-307		-0.64	386.19122	11.70			
Y1	$C_{21}H_{14}FNO$	N- alkyl side chain removal	-(C5 H10)	-0.58	316.11304	9.58			
Y2	$C_{21}H_{14}FNO_2$	N- alkyl side chain removal+Hydroxylation（Pyrrole ring）	-(C5 H10) +(O)	-0.67	332.10791	8.47			

Y3	$C_{26}H_{25}NO$	Defluorination	-(F) +(H)	-0.73	368.20062	11.82			
Y4	$C_{26}H_{23}NO_2$	Oxidative defluoridation+Dehydrogenation	-(H F) +(O)				-3.68	382.17875	2.15
Y5	$C_{26}H_{22}FNO$	Dehydrogenation（N-alkyl side chains）	-(H2)	-1.29	384.17532	11.24			
Y6	$C_{26}H_{22}FNO_2$	Dehydrogenation+Hydroxylation（N-alkyl side chains）	-(H2) +(O)	-1.27	400.17023	9.85			
Y7	$C_{26}H_{24}FNO_2$	Hydroxylation（N-alkyl side chains）	+(O)	-1.02	402.18597	9.43			
Y8	$C_{26}H_{24}FNO_2$	Hydroxylation(Naphthyl)	+(O)	-0.87	402.18604	10.70			
Y9	$C_{26}H_{22}FNO_3$	Ketone formation+Hydroxylation(Naphthyl)	-(H2) +(O2)	-1.25	416.16513	8.80			
Y10	$C_{26}H_{24}FNO_3$	Dihydroxylation（Naphthyl+Phenyl）	+(O2)	-1.3	418.18076	8.56			
Y11	$C_{26}H_{26}FNO_3$	Dihydrodiol	+(H2 O2)	-1.28	420.19641	9.09			
Y12	$C_{26}H_{26}FNO_4$	Hydroxylation+Dihydrodiol(Naphthyl)	+(H2 O3)	-1.12	436.19138	9.14			
Y13	$C_{28}H_{26}FNO_4$	Dihydroxylation+Acetylization	+(C2 H2 O3)				2.56	460.19304	3.03
Y14	$C_{26}H_{25}NO_5S$	Oxidative defluoridation+Sulfation	-(F) +(H O4 S)				-0.18	464.15254	0.82
Y15	$C_{26}H_{22}FNO_5S$	Ketone formation+Sulfation	-(H2) +(O4 S)				-2.35	480.12642	0.82
Y16	$C_{31}H_{36}FN_3O_3$	Hydroxylation+Ornithine binding	+(C5 H12 N2 O2)				0.1	518.2814	4.91

Y17	$C_{32}H_{35}N_5O_2$	Defluorination+Arginine binding	-(F) +(C6 H11 N4 O)				-2.67	522.28496	7.28
Y18	$C_{32}H_{32}FNO_8$	Hydroxylation(Naphthyl)+Glucuronidation	+(C6 H8 O7)	-1.1	578.21783	8.05			
Y19	$C_{32}H_{32}FNO_9$	Dihydroxylation(Naphthyl)+Glucuronidation	+(C6 H8 O8)	-1.14	594.21271	7.30			
Y20	$C_{32}H_{34}FNO_9$	Dihydrodiol+Glucuronidation	+(C6 H10 O8)	-0.98	596.22845	8.32			

Table26 Information of JWH-370 and it's metabolites

Name	Formula	Transformations	Composition Change	*In vitro* metabolites			*In vivo* metabolites		
				Annot. DeltaMass [ppm]	m/z	RT [min]	Annot. DeltaMass [ppm]	m/z	RT [min]
Z0	$C_{27}H_{27}NO$	JWH-370		-0.37	382.2164	12.81	-1.87	382.21583	12.01
Z1	$C_{22}H_{17}NO$	N- alkyl side chain removal	-(C5 H10)	-0.74	312.13806	10.43			
Z2	$C_{22}H_{17}NO_2$	N- alkyl side chain removal+Hydroxylation（Pyrrole ring）	-(C5 H10) +(O)	-1.11	328.13284	9.11			
Z3	$C_{22}H_{17}NO_4S$	N- alkyl side chain removal+Sulfation	-(C5 H10) +(O3 S)				-0.98	392.09472	0.89
Z4	$C_{27}H_{25}NO_2$	Dehydrogenation+Hydroxylation	-(H2) +(O)	-0.78	396.1955	10.89			
Z5	$C_{27}H_{27}NO_2$	Hydroxylation（ Pyrrole ring）	+(O)	-0.84	398.21112	11.81			
Z6	$C_{27}H_{27}NO_2$	Hydroxylation（ Naphthyl）	+(O)	-0.77	398.21115	10.48			

Z7	$C_{27} H_{25} N O_3$	Dehydrogenation+Dihydroxylation	-(H2) +(O2)				-1.98	412.1899	9.47
Z8	$C_{27} H_{27} N O_3$	Dihydroxylation（Naphthyl）	+(O2)	-1.28	414.20584	8.99			
Z9	$C_{27} H_{29} N O_3$	Dihydrodiol	+(H2 O2)	-0.82	416.22168	10.43			
Z10	$C_{33} H_{37} N O_6$	Glycoside binding	+(C6 H10 O5)				-4.75	544.26678	8.18
Z11	$C_{33} H_{35} N O_8$	Hydroxylation（Naphthyl）+Glucuronidation	+(C6 H8 O7)	-0.77	574.2431	9.07			
Z12	$C_{33} H_{35} N O_9$	Dihydroxylation（Naphthyl）+Glucuronidation	+(C6 H8 O8)	-0.82	590.23798	10.03			
Z13	$C_{33} H_{37} N O_9$	Dihydrodiol+Glucuronidation	+(C6 H10 O8)	-0.96	592.25354	9.34			
Z14	$C_{33} H_{35} N O_8$	Hydroxylation+Glucuronidation	+(C6 H8 O7)				4.74	596.2282	7.85
