# Supplementary figures and images for: Systematic Characterization of In Vitro and In Vivo Metabolic Pathways and Identification of Novel Biomarkers of 26 Synthetic Cannabinoids
Source: Molecules. 2025 Jun 21;30(13):2682. doi: 10.3390/molecules30132682 (PMC12250893; doi:10.3390/molecules30132682)

# 5F-EMB-PINACA

Indazole amides

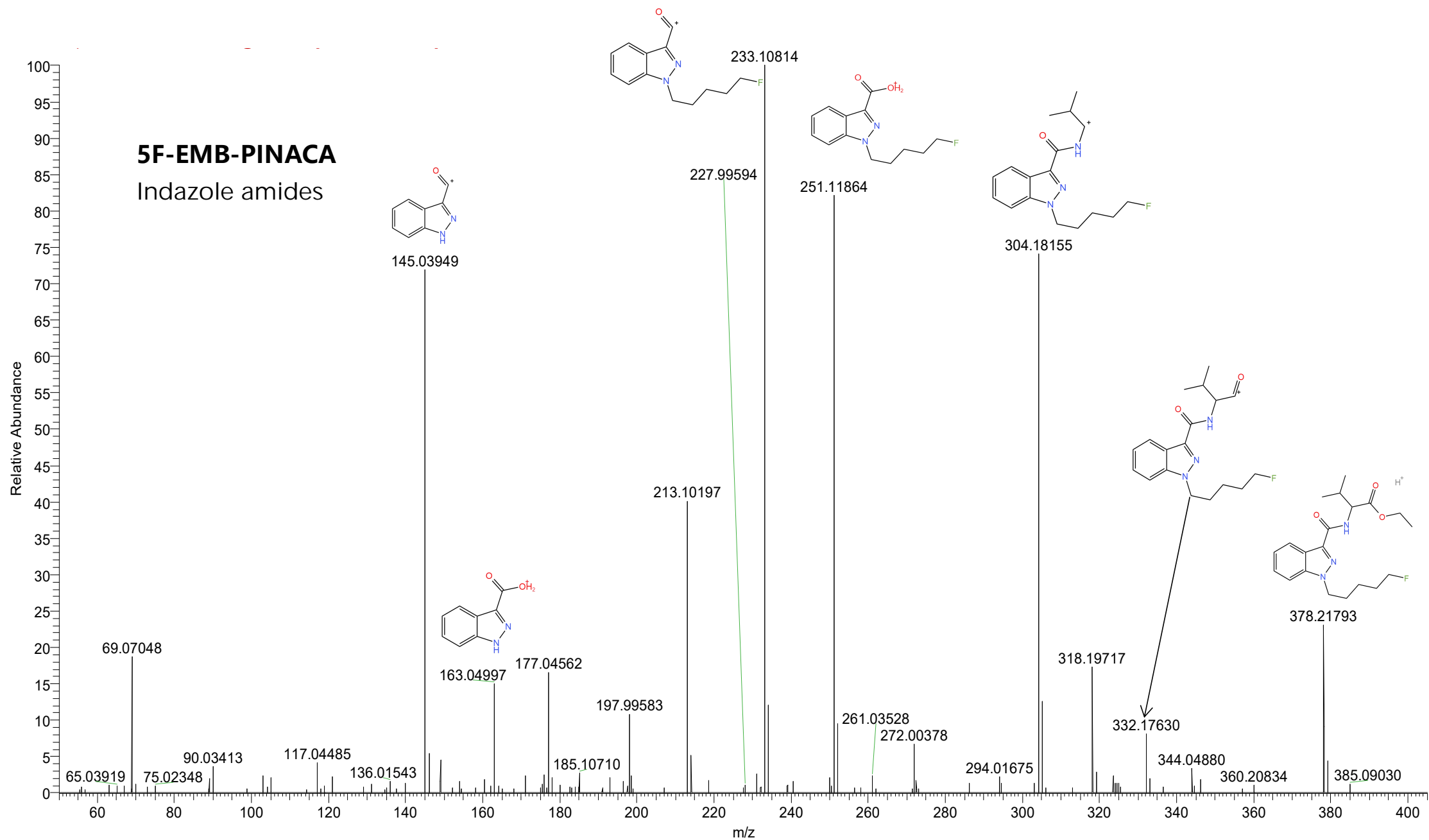

# B.5F-MDMB-PICA

Indole amides

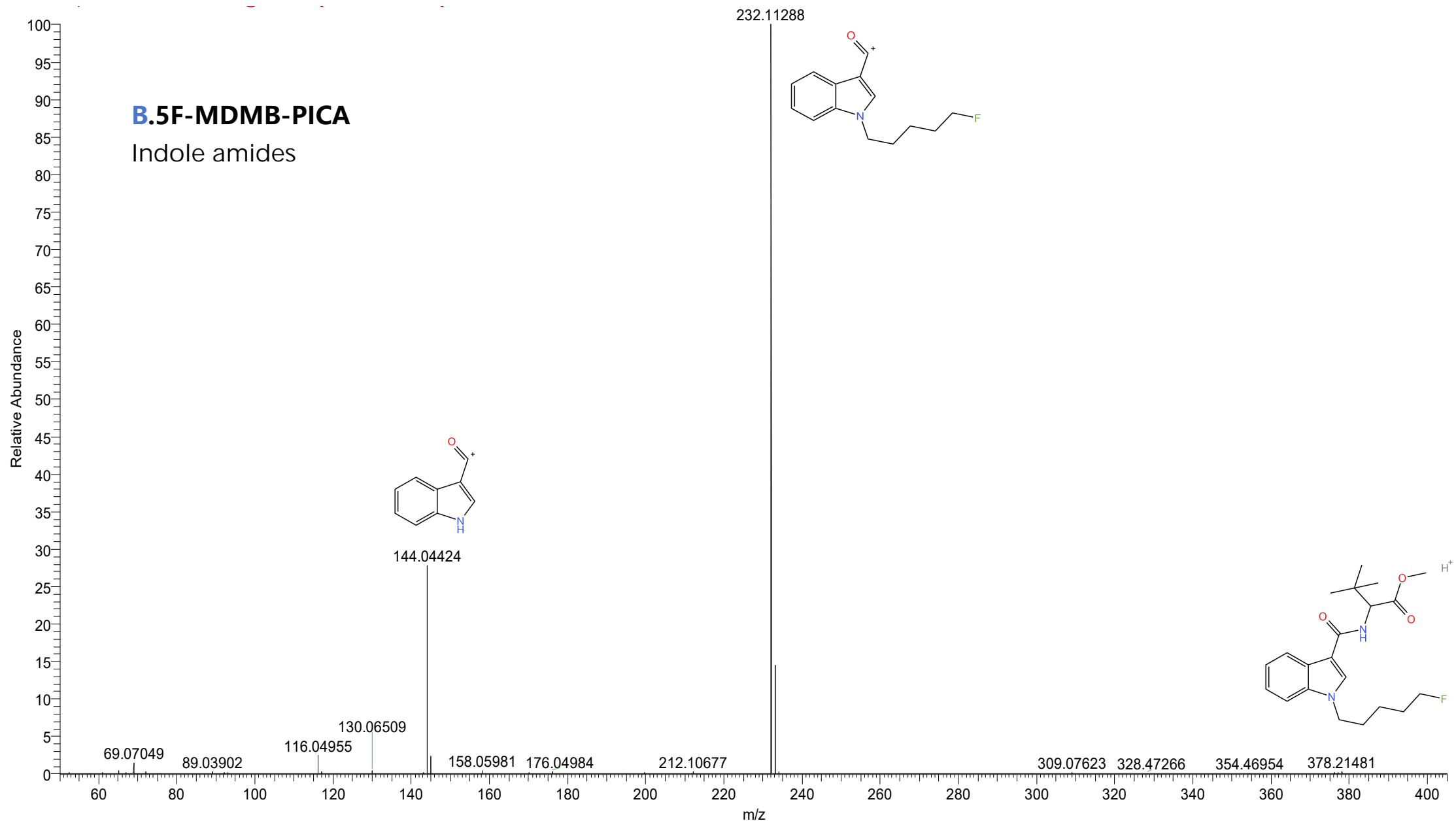

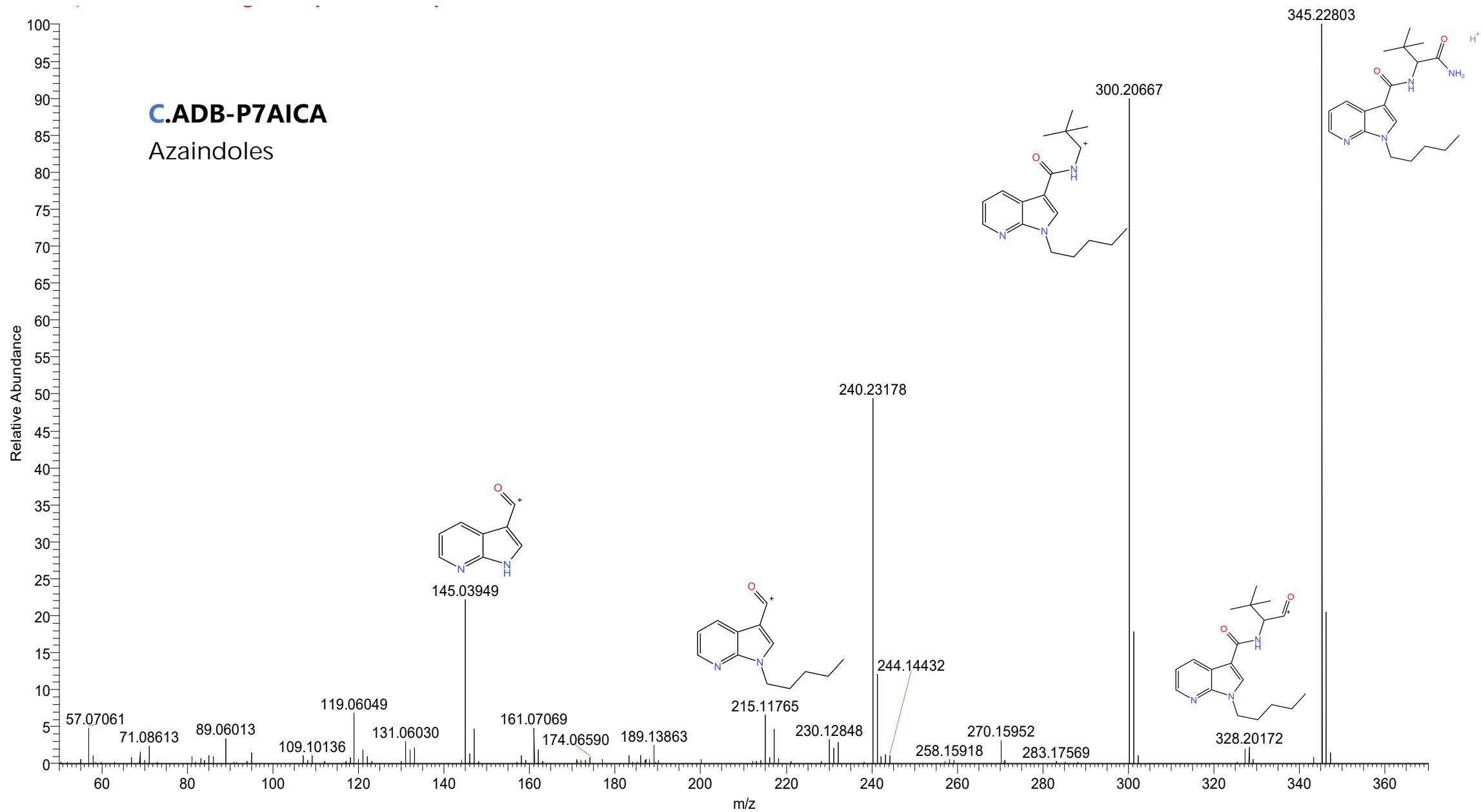

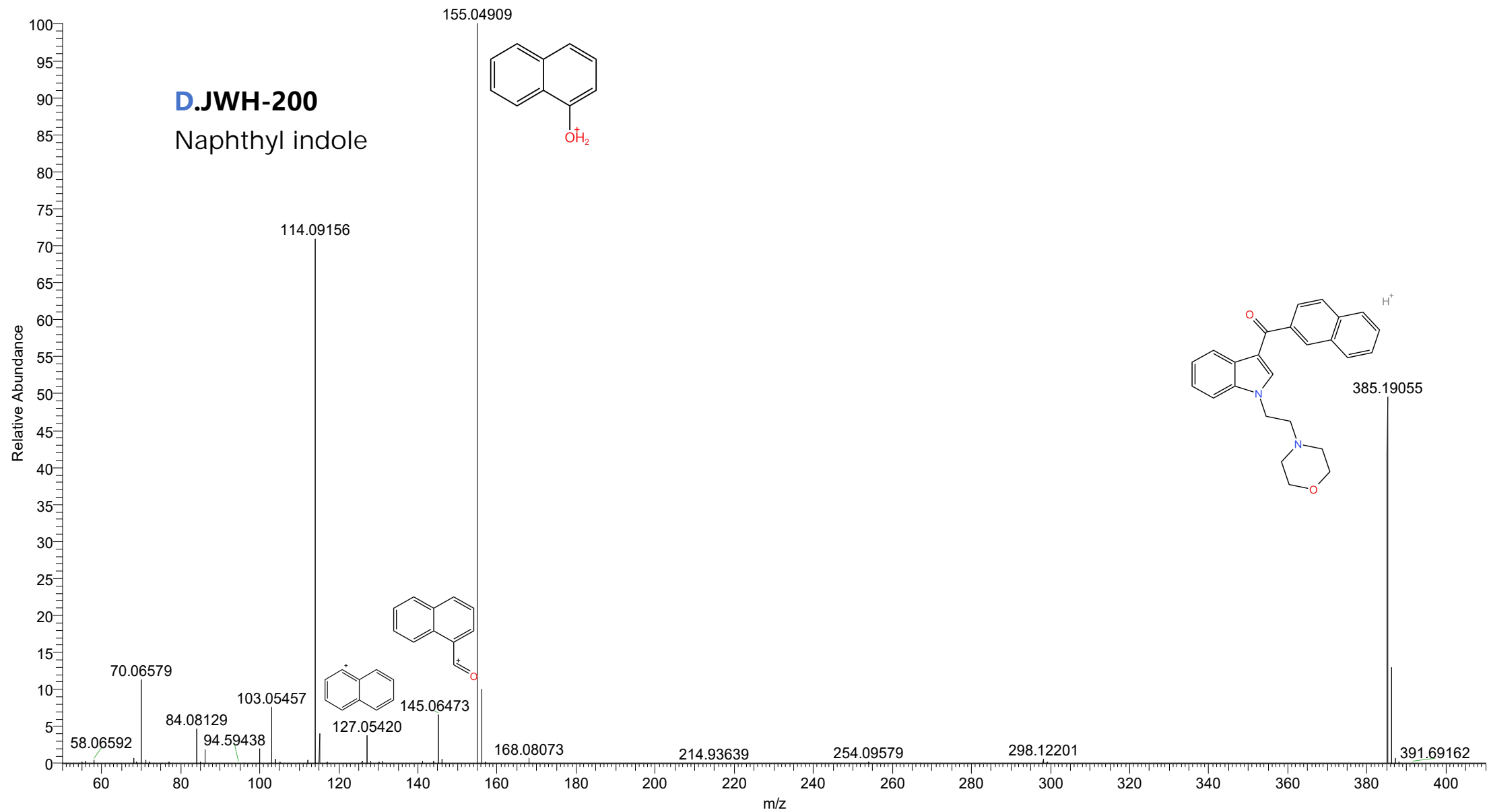

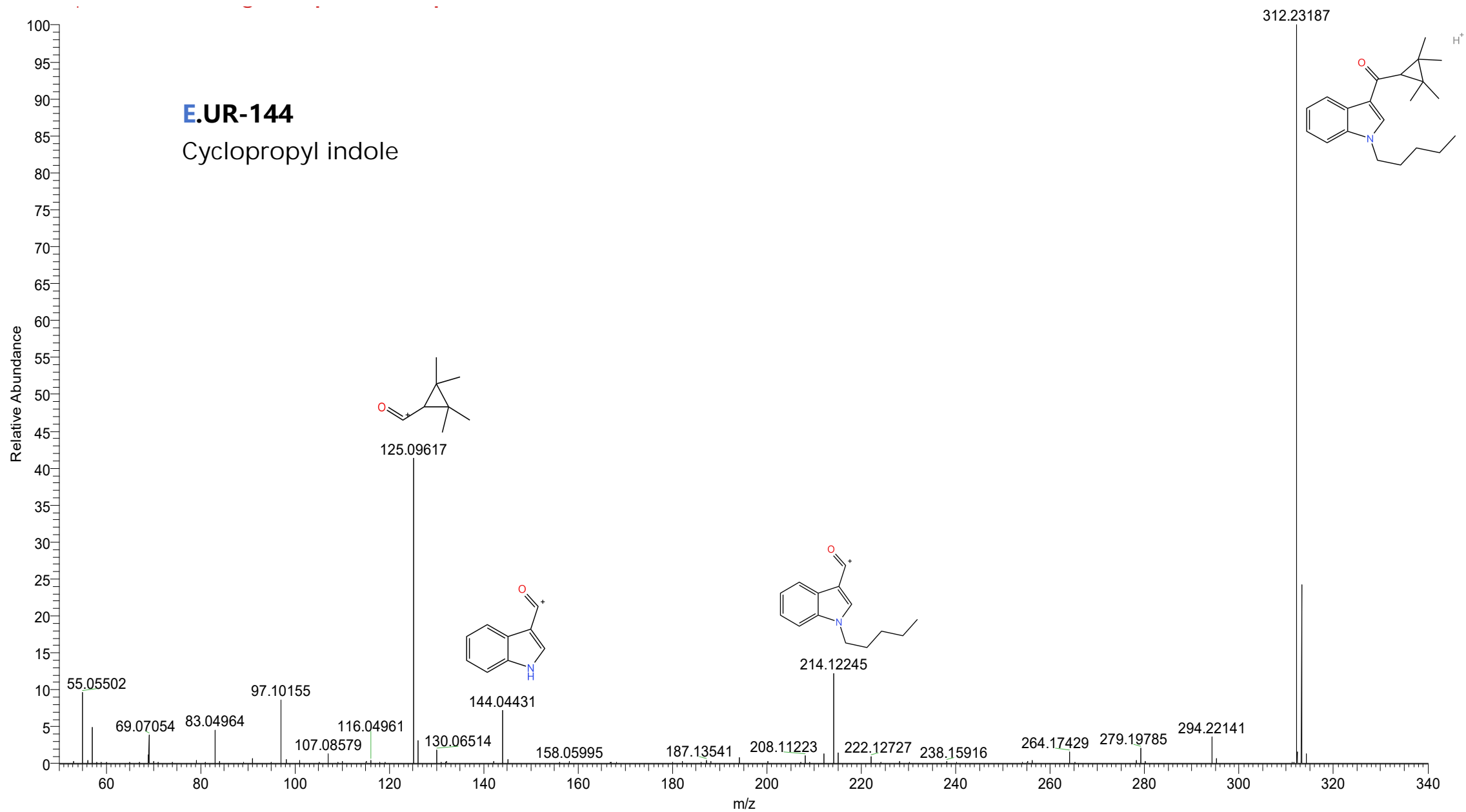

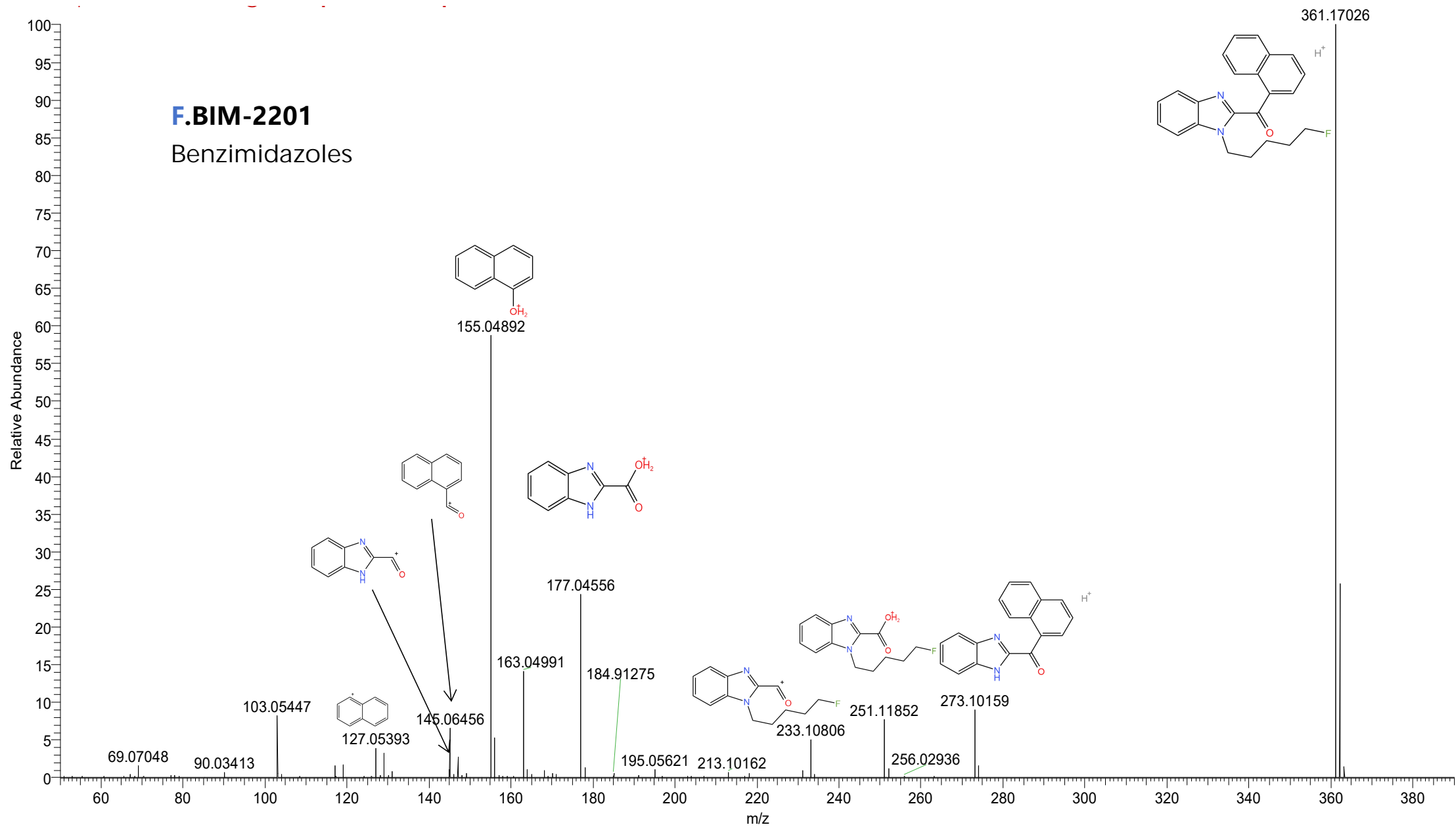

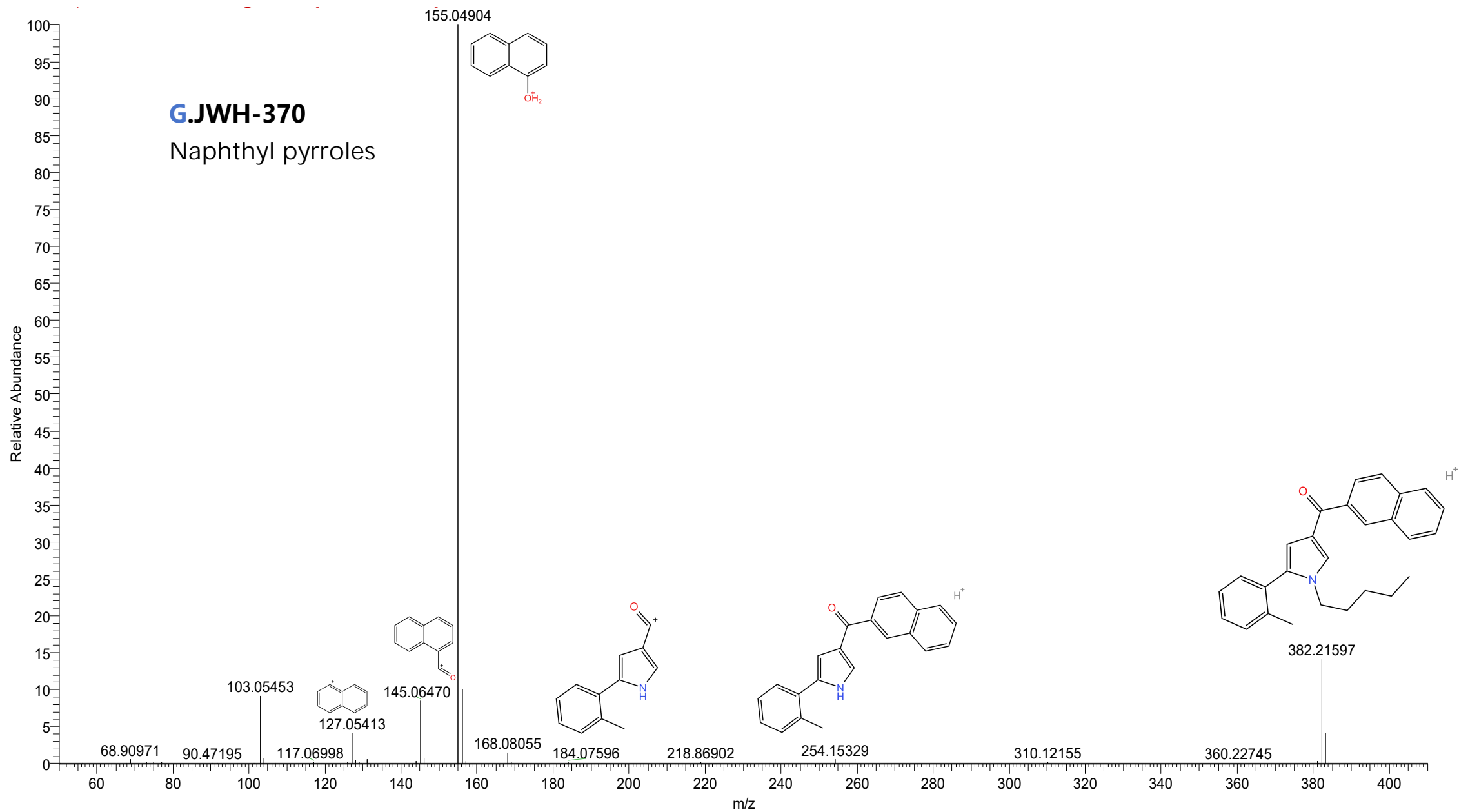

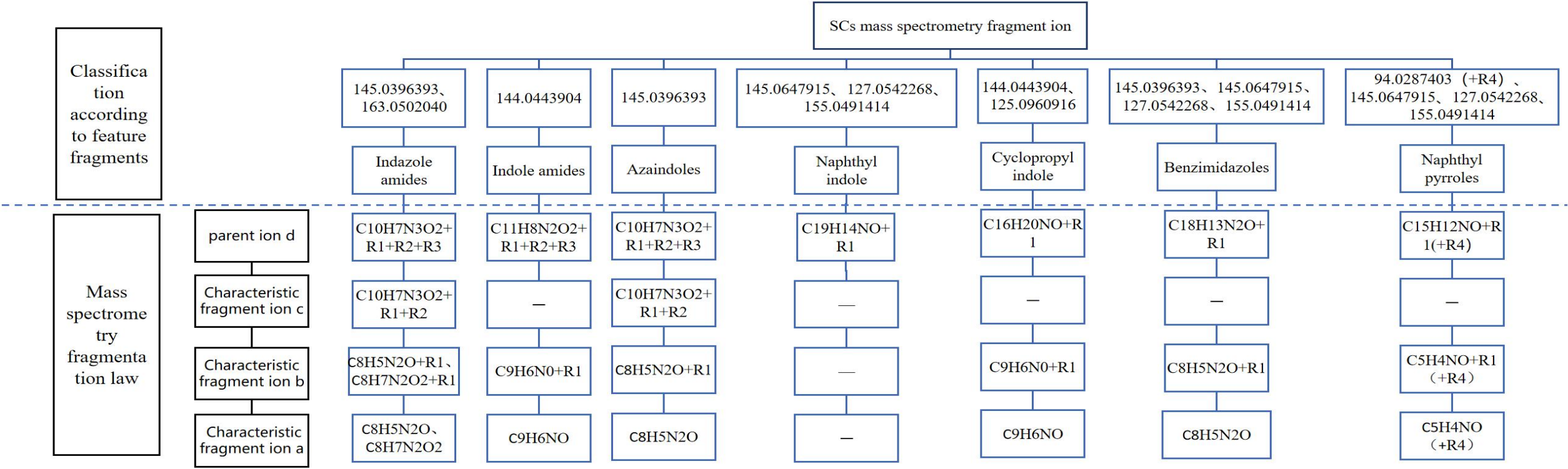

Supplement: Supplementary file 1 [file molecules-30-02682-s001.zip › Supplementary Materials-Figure S1.pdf]
